# Supplementary figures and images for: ONC201/TIC10 enhances durability of mTOR inhibitor everolimus in metastatic ER+ breast cancer
Source: eLife. 2023 Sep 29;12:e85898. doi: 10.7554/eLife.85898 (PMC10541180; doi:10.7554/eLife.85898)

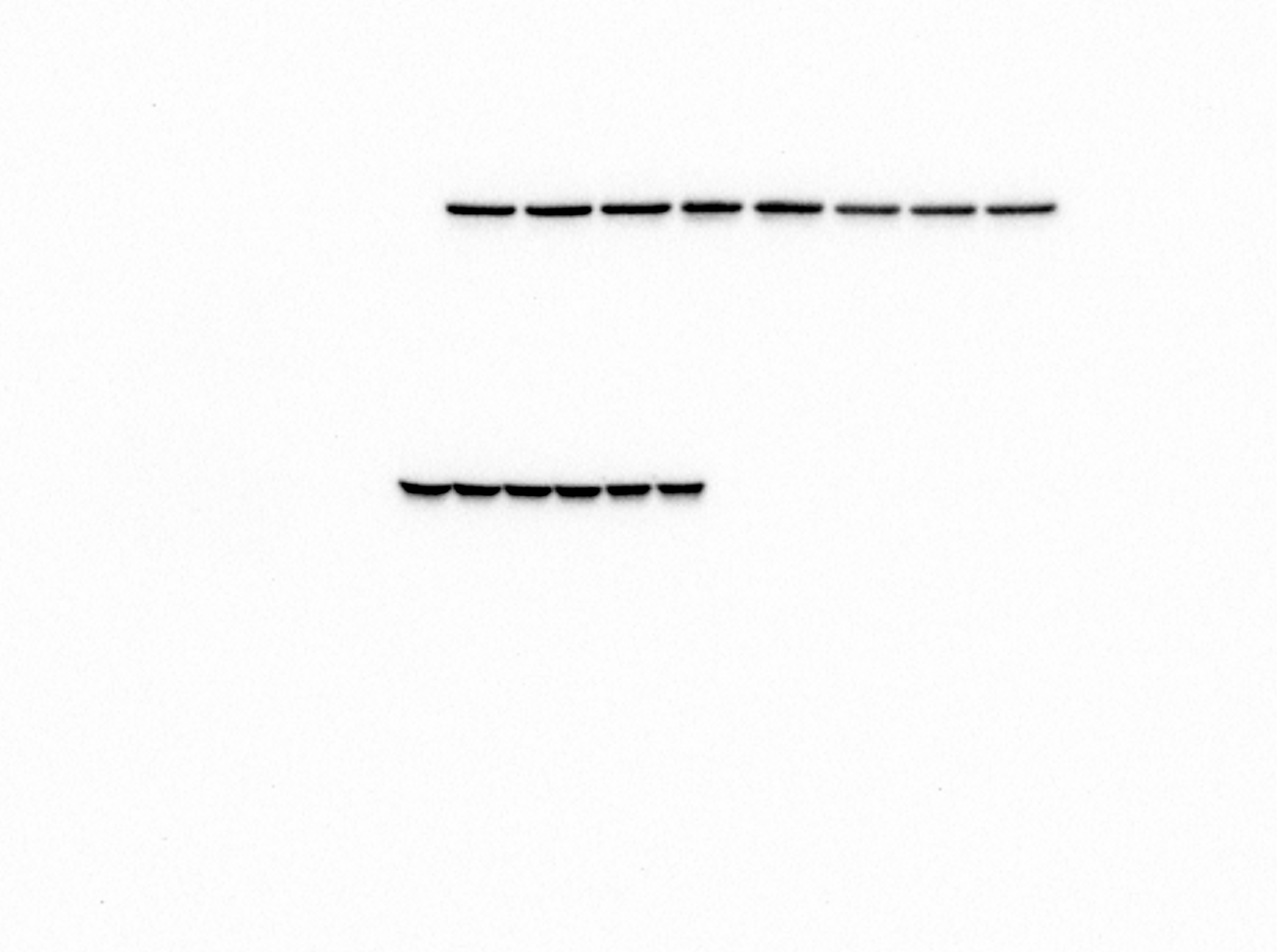

Supplement: Figure 4—source data 1. [file elife-85898-fig4-data1.zip › Figure 4-source data 1/CAMA actin lower part.tif]

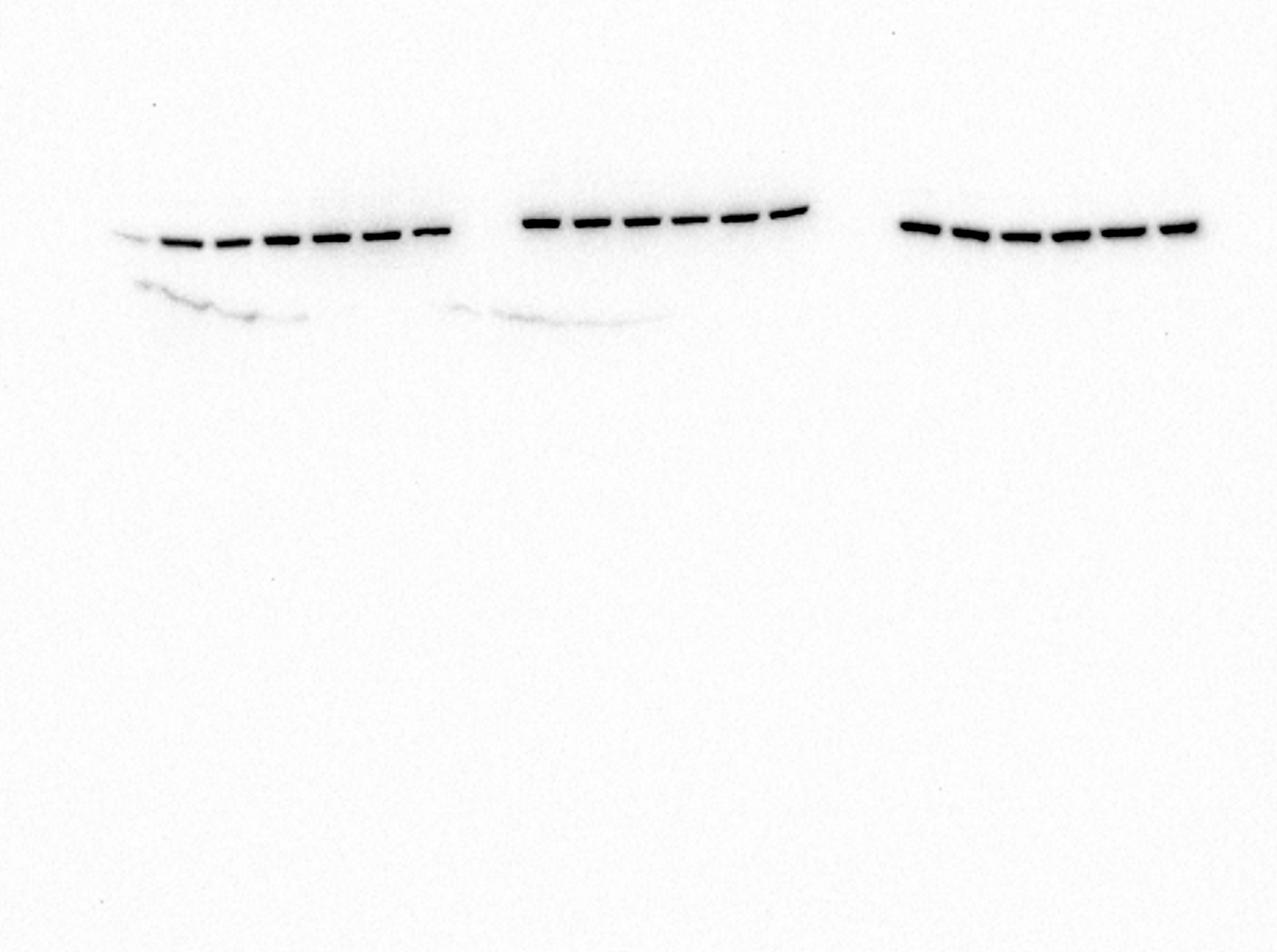

Supplement: Figure 4—source data 1. [file elife-85898-fig4-data1.zip › Figure 4-source data 1/CAMA actin OXPHOS left part.tif]

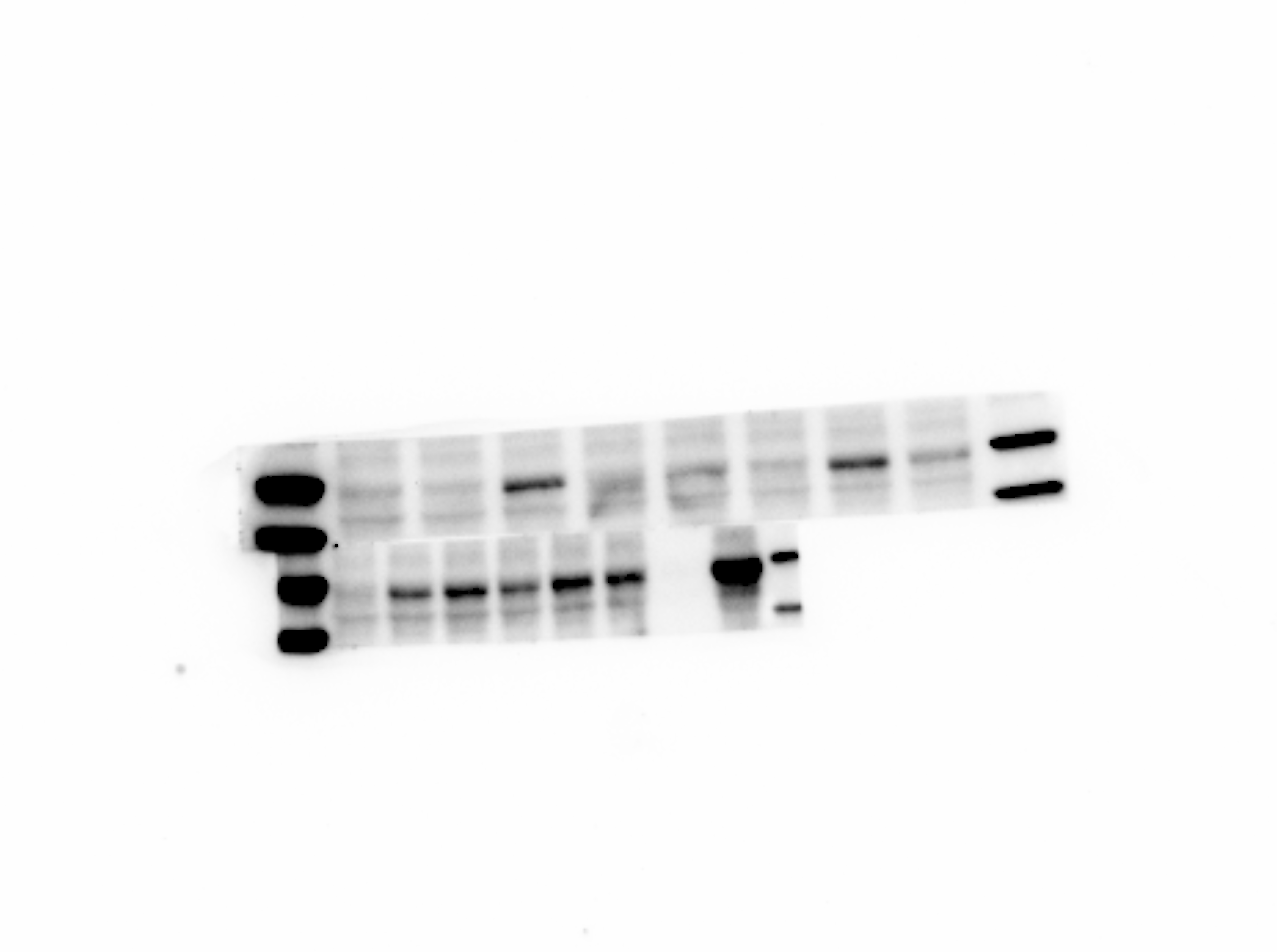

Supplement: Figure 4—source data 1. [file elife-85898-fig4-data1.zip › Figure 4-source data 1/CAMA ATF4 lower part.tif]

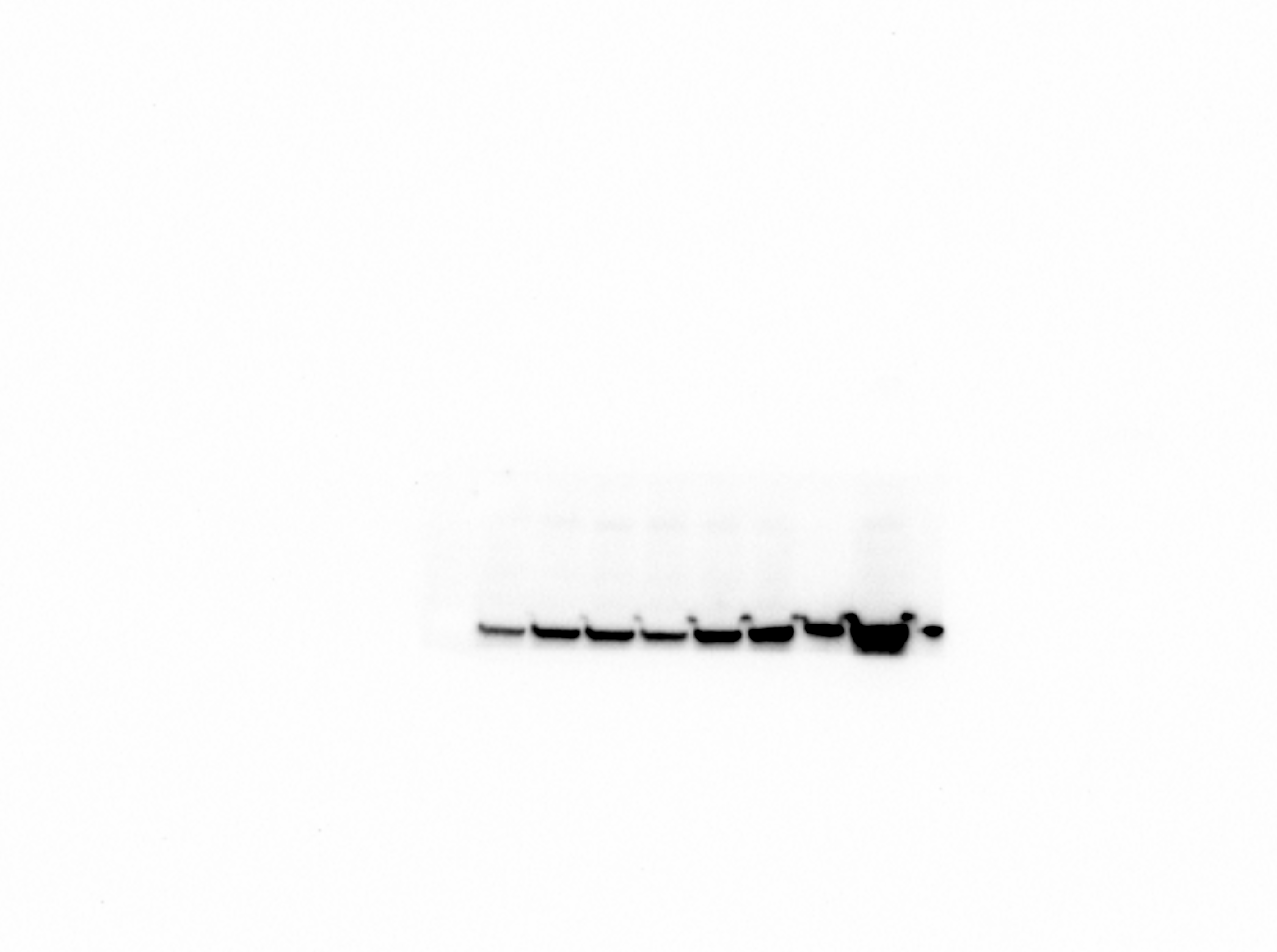

Supplement: Figure 4—source data 1. [file elife-85898-fig4-data1.zip › Figure 4-source data 1/CAMA BiP.tif]

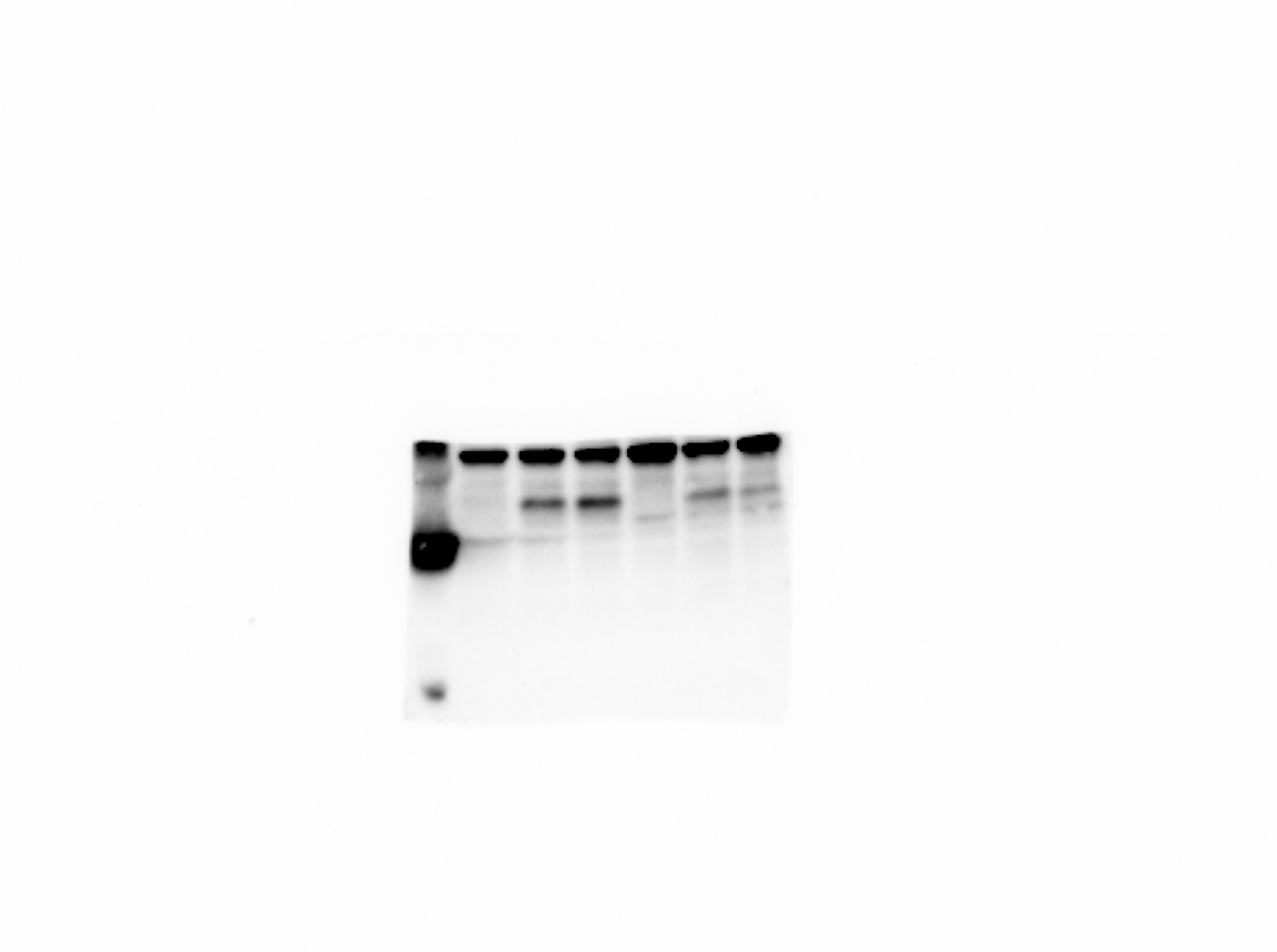

Supplement: Figure 4—source data 1. [file elife-85898-fig4-data1.zip › Figure 4-source data 1/CAMA CHOP.tif]

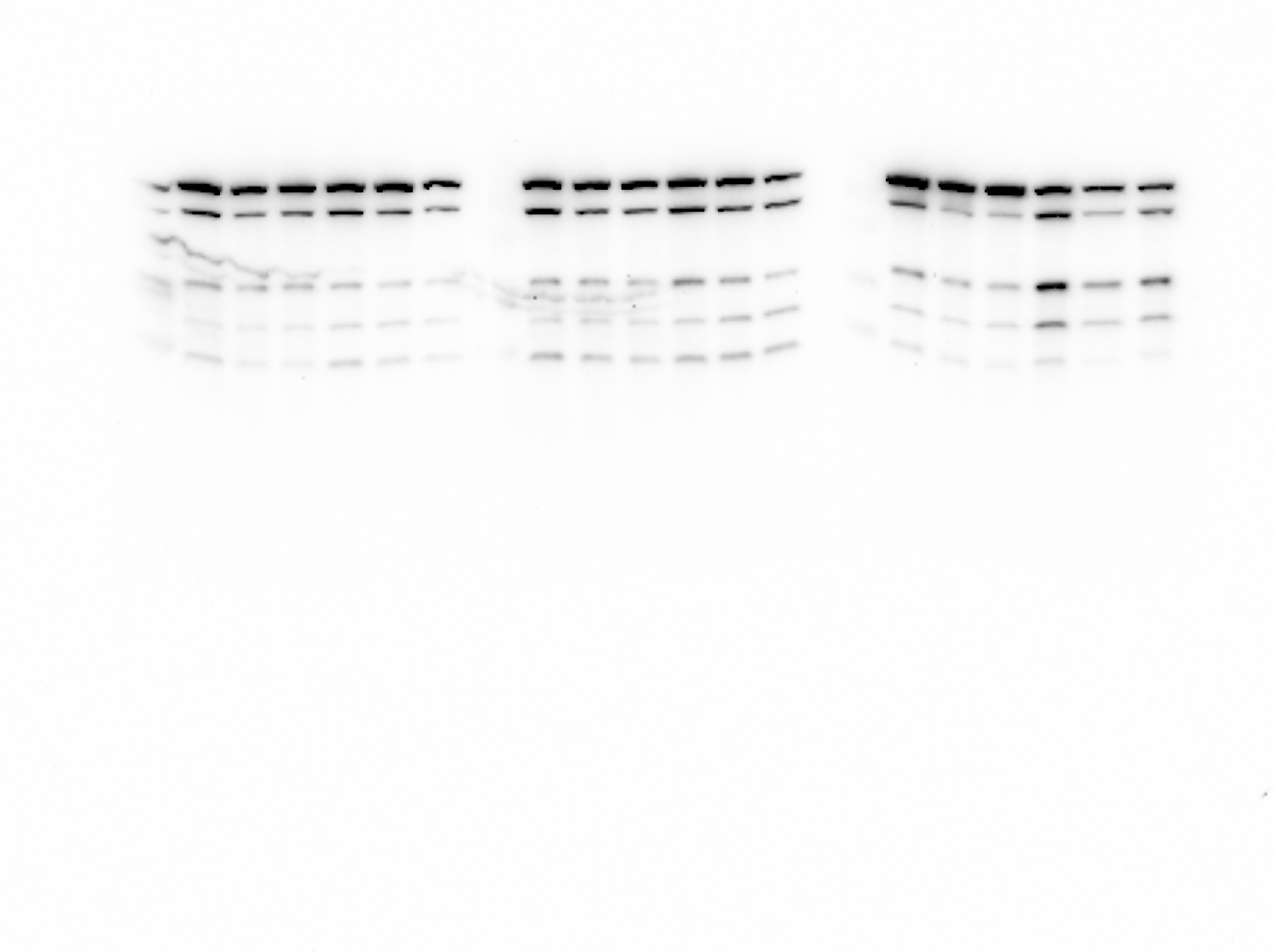

Supplement: Figure 4—source data 1. [file elife-85898-fig4-data1.zip › Figure 4-source data 1/CAMA OXPHOS left part 1.tif]

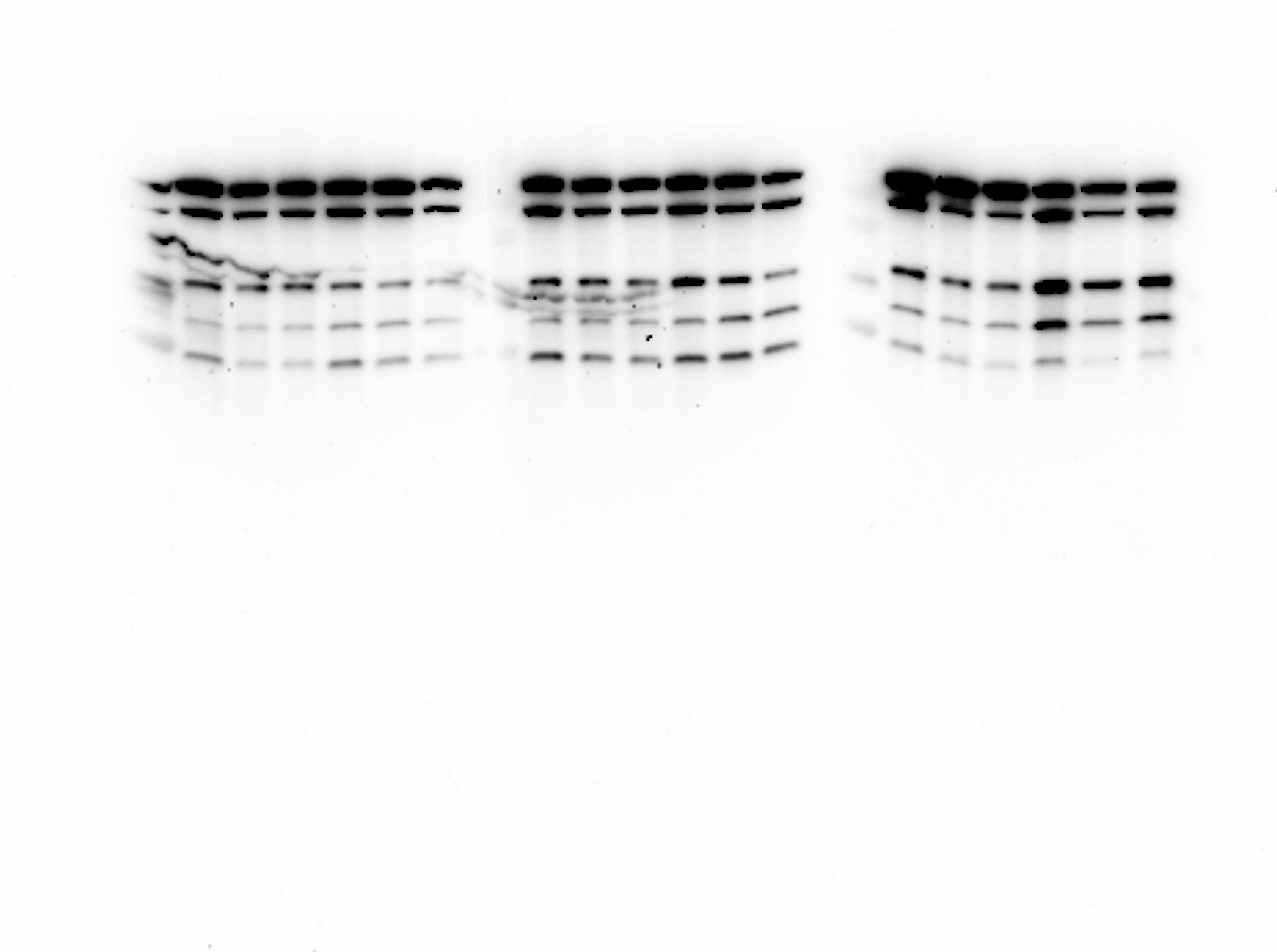

Supplement: Figure 4—source data 1. [file elife-85898-fig4-data1.zip › Figure 4-source data 1/CAMA OXPHOS left part 2.tif]

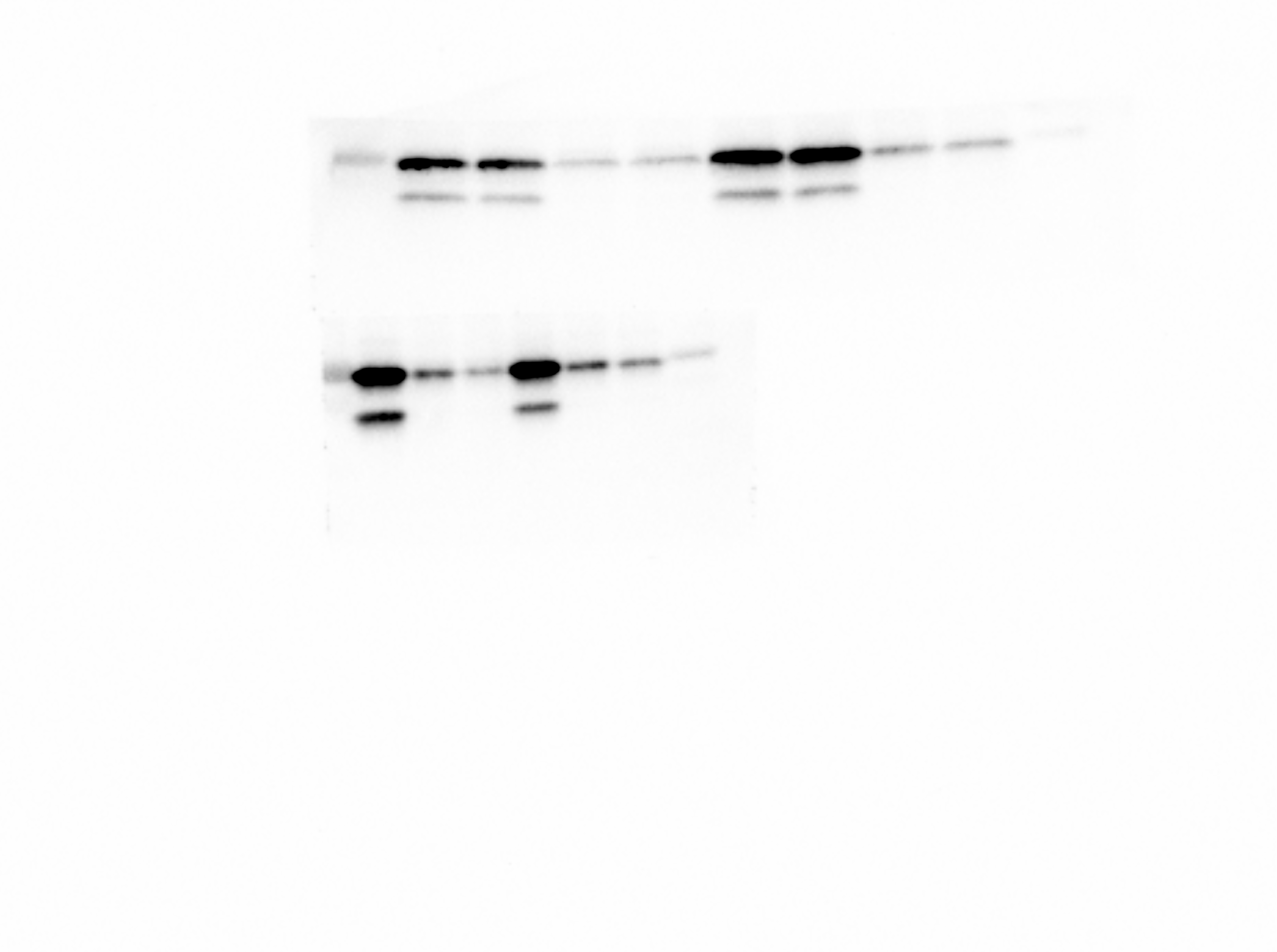

Supplement: Figure 4—source data 1. [file elife-85898-fig4-data1.zip › Figure 4-source data 1/CAMA TFAM lower part.tif]

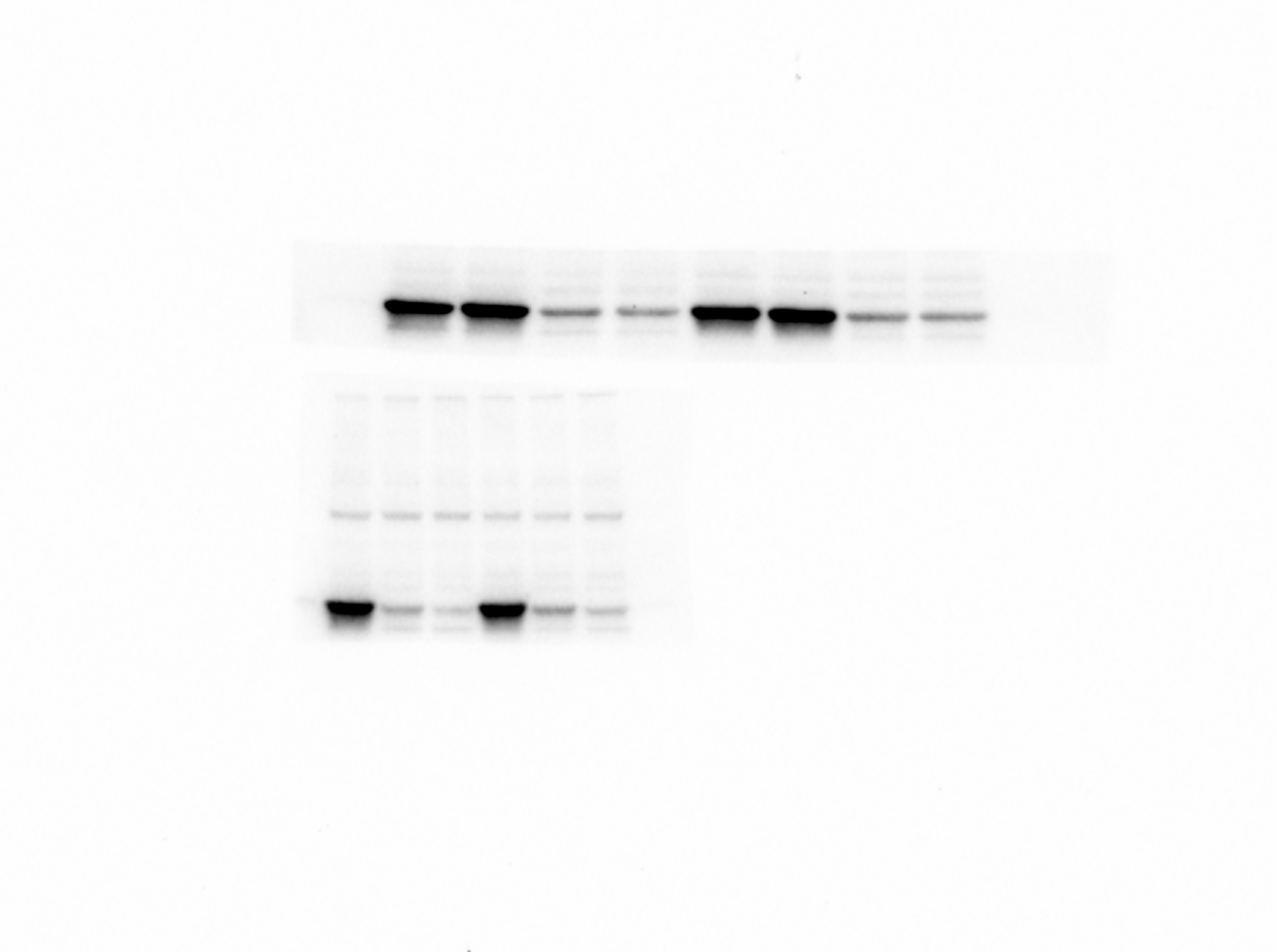

Supplement: Figure 4—source data 1. [file elife-85898-fig4-data1.zip › Figure 4-source data 1/CAMA TUFM lower part.tif]

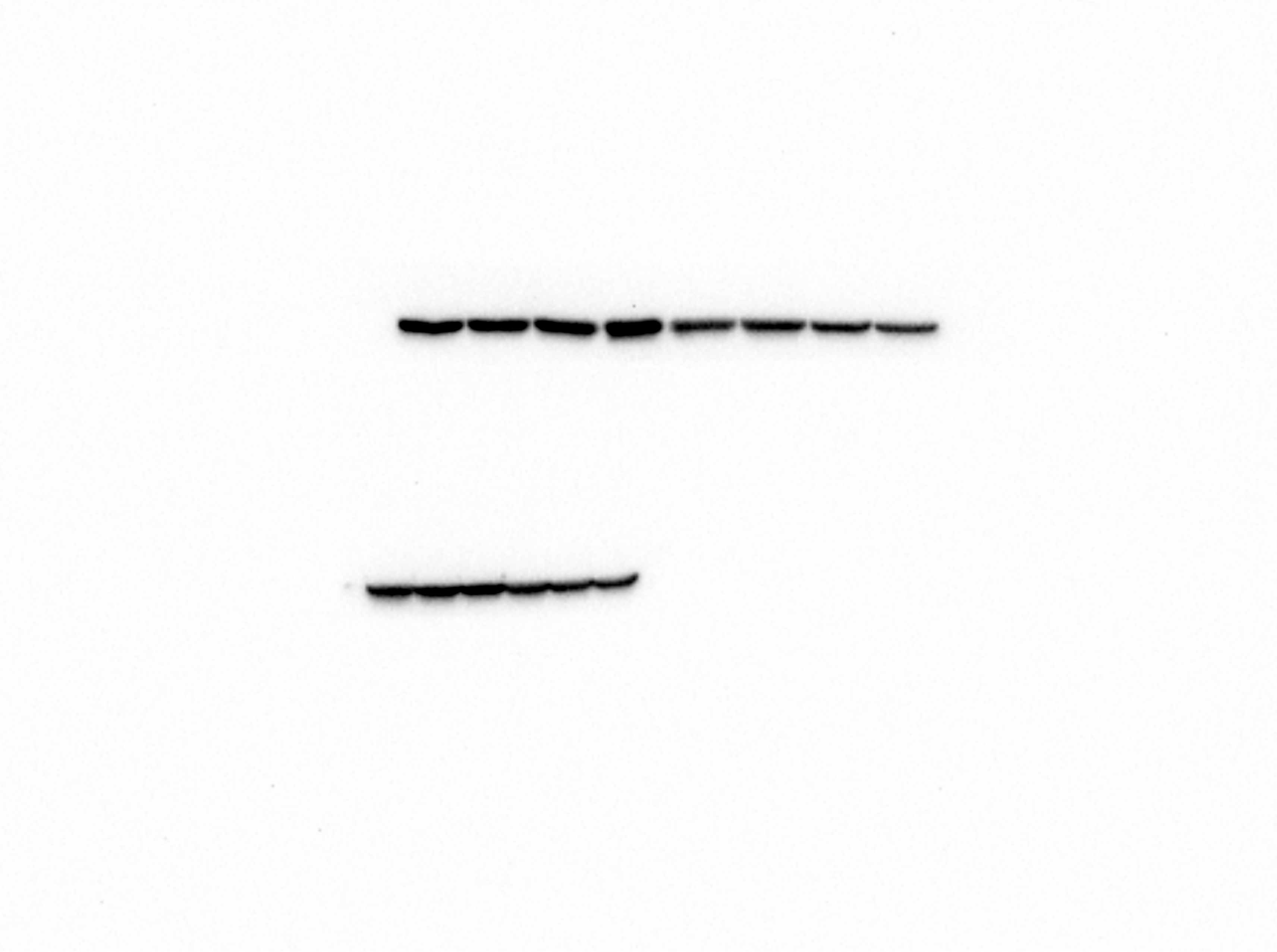

Supplement: Figure 4—source data 1. [file elife-85898-fig4-data1.zip › Figure 4-source data 1/MCF7 actin lower part.tif]

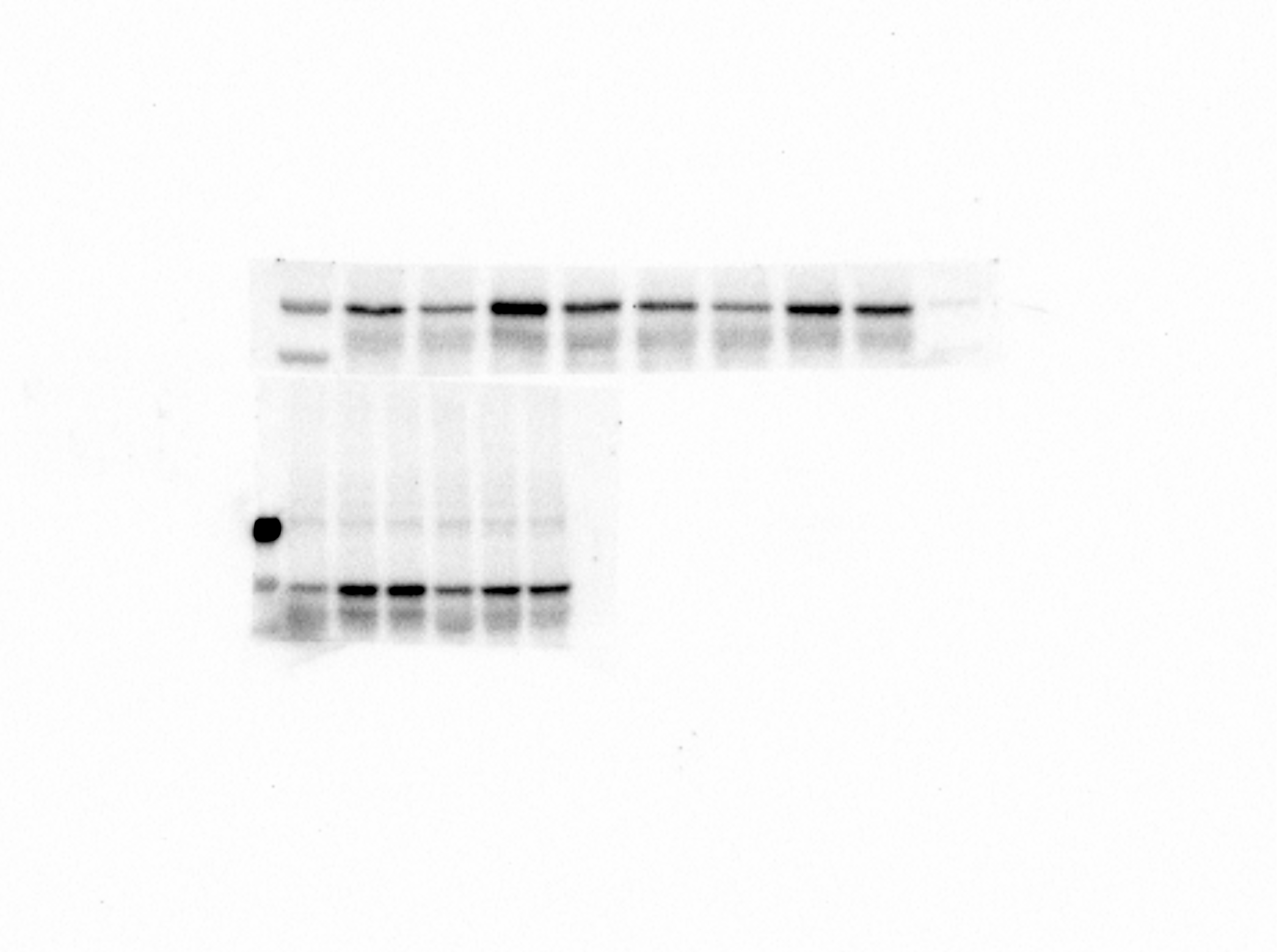

Supplement: Figure 4—source data 1. [file elife-85898-fig4-data1.zip › Figure 4-source data 1/MCF7 ATF4 lower part.tif]

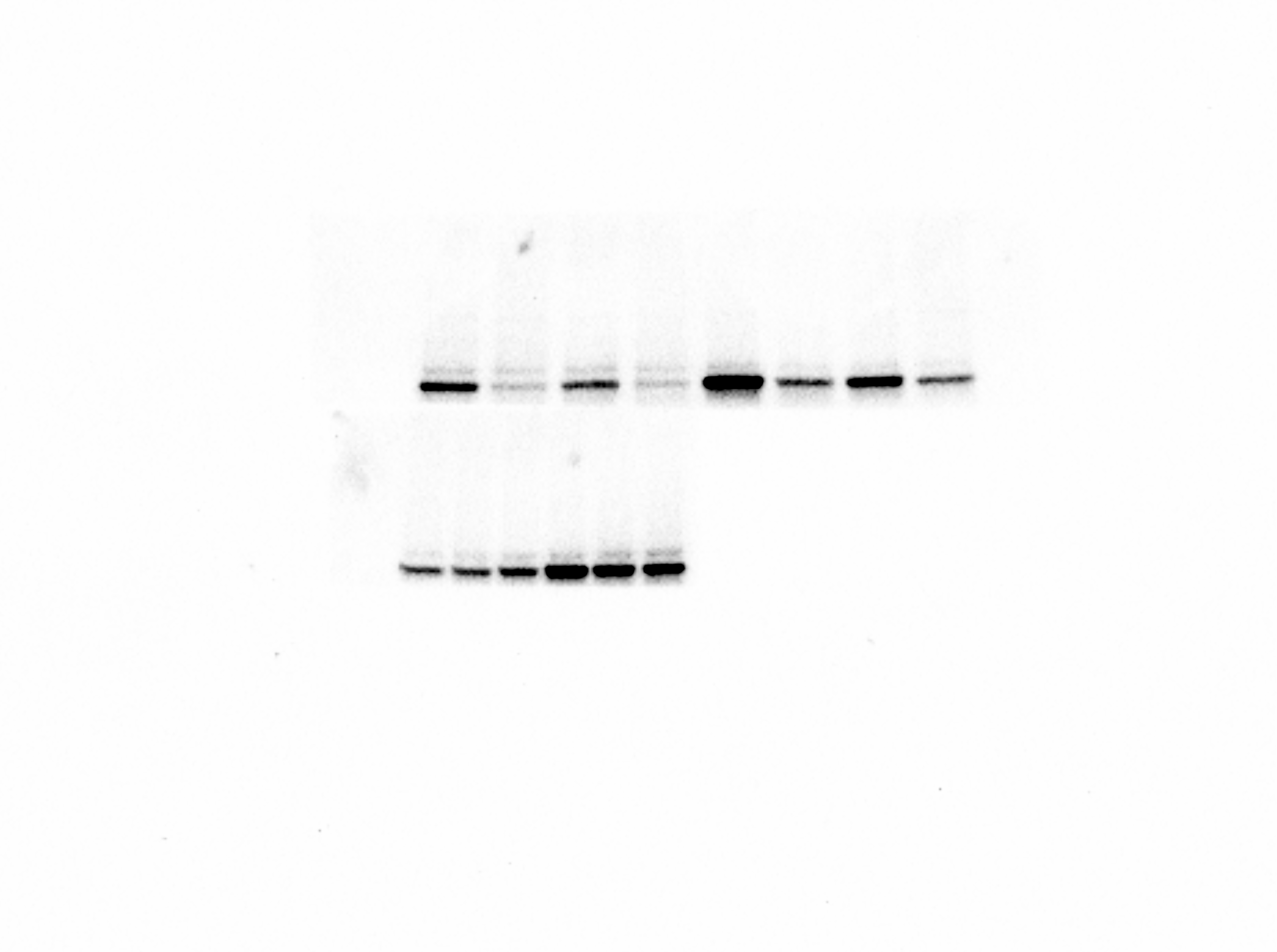

Supplement: Figure 4—source data 1. [file elife-85898-fig4-data1.zip › Figure 4-source data 1/MCF7 BiP lower part.tif]

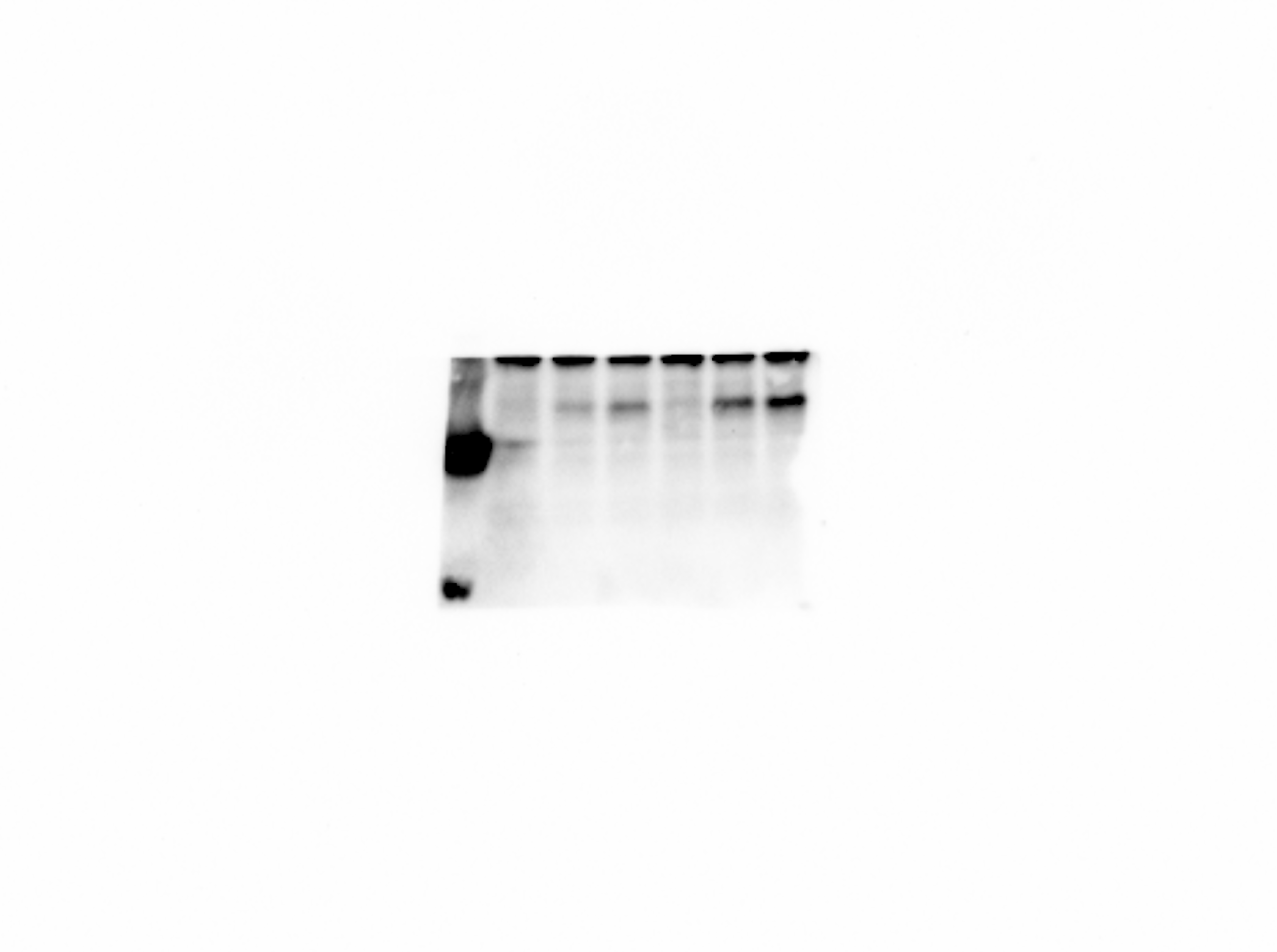

Supplement: Figure 4—source data 1. [file elife-85898-fig4-data1.zip › Figure 4-source data 1/MCF7 CHOP.tif]

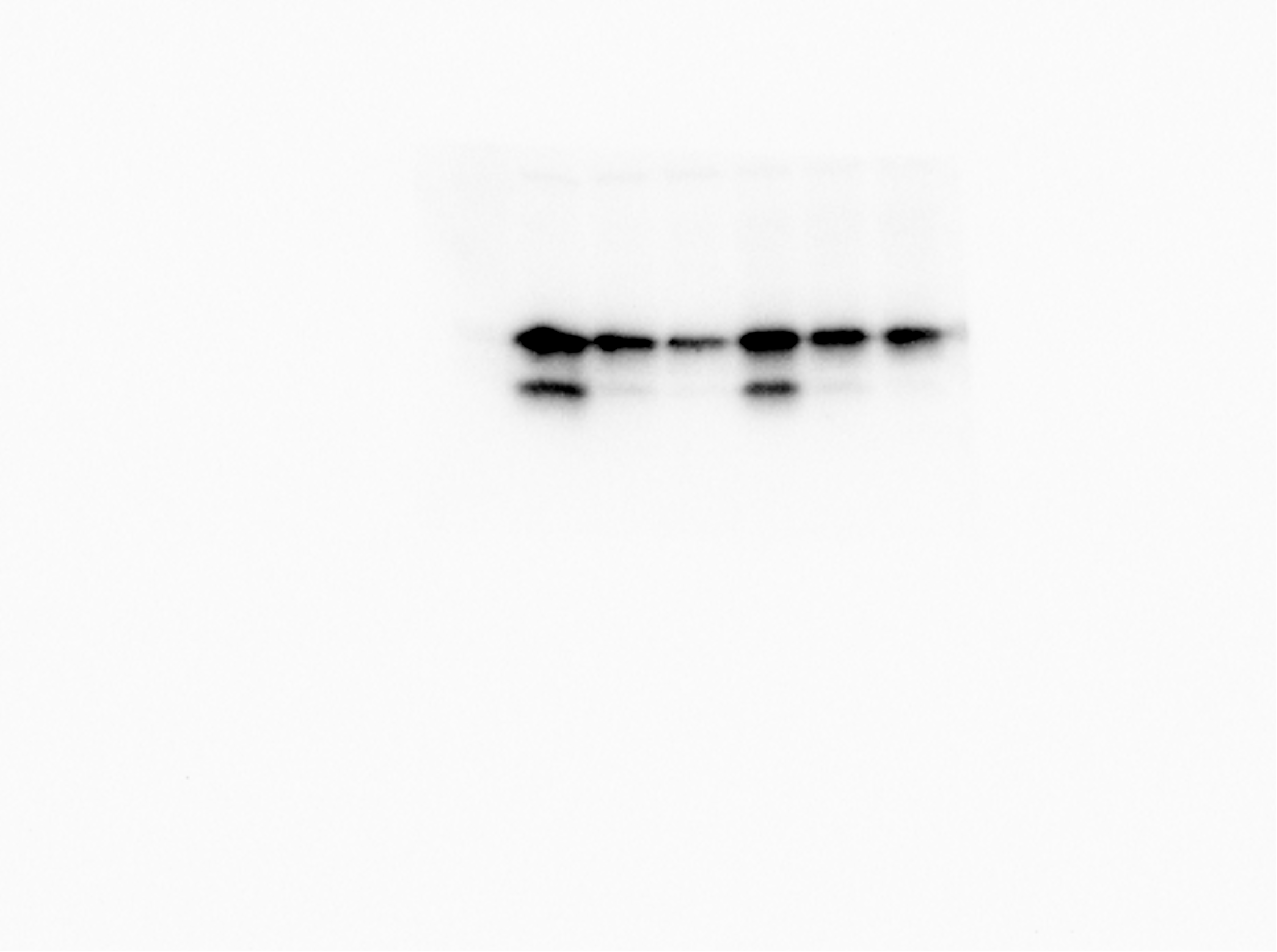

Supplement: Figure 4—source data 1. [file elife-85898-fig4-data1.zip › Figure 4-source data 1/MCF7 TFAM.tif]

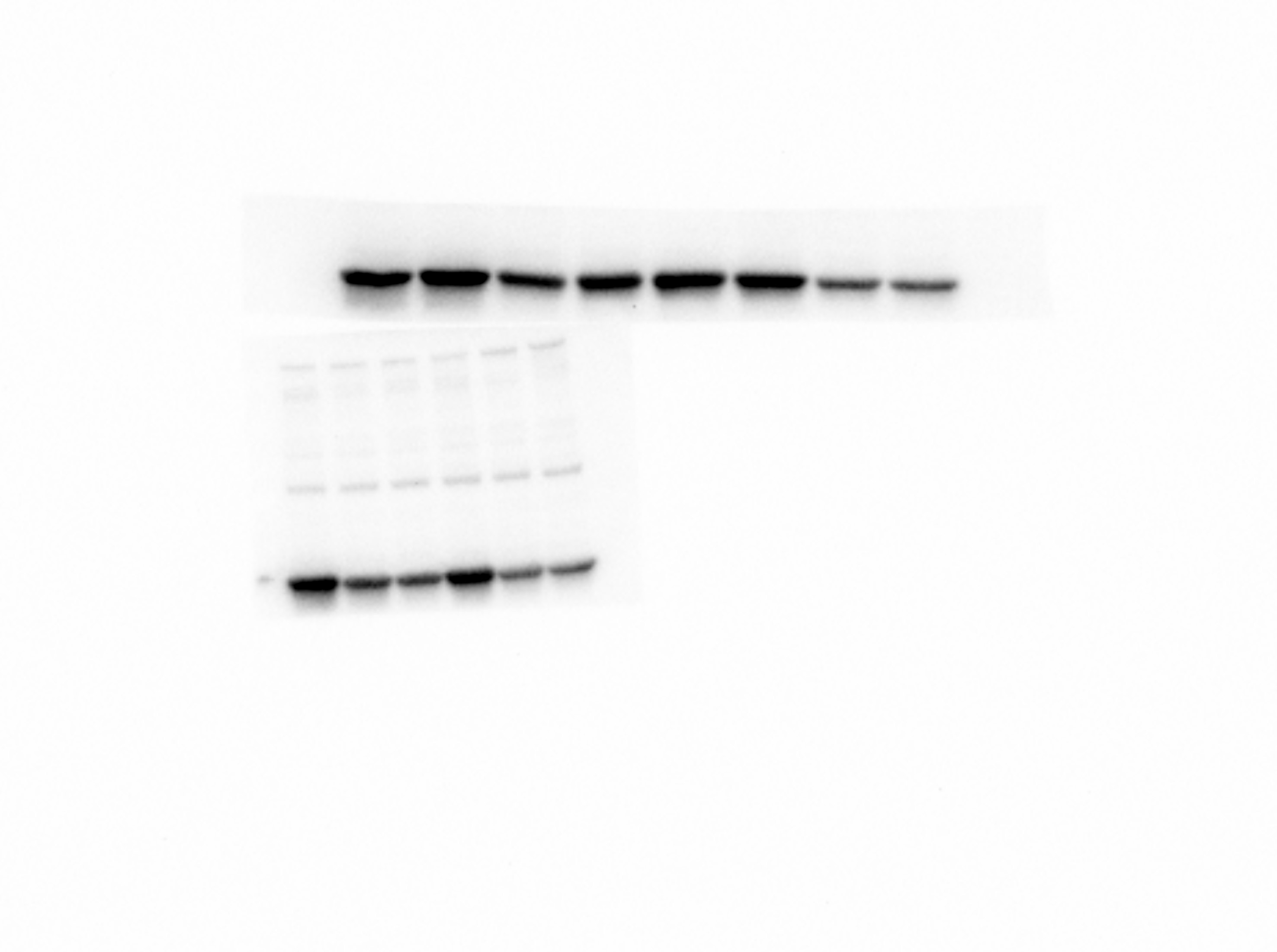

Supplement: Figure 4—source data 1. [file elife-85898-fig4-data1.zip › Figure 4-source data 1/MCF7 TUFM lower part.tif]

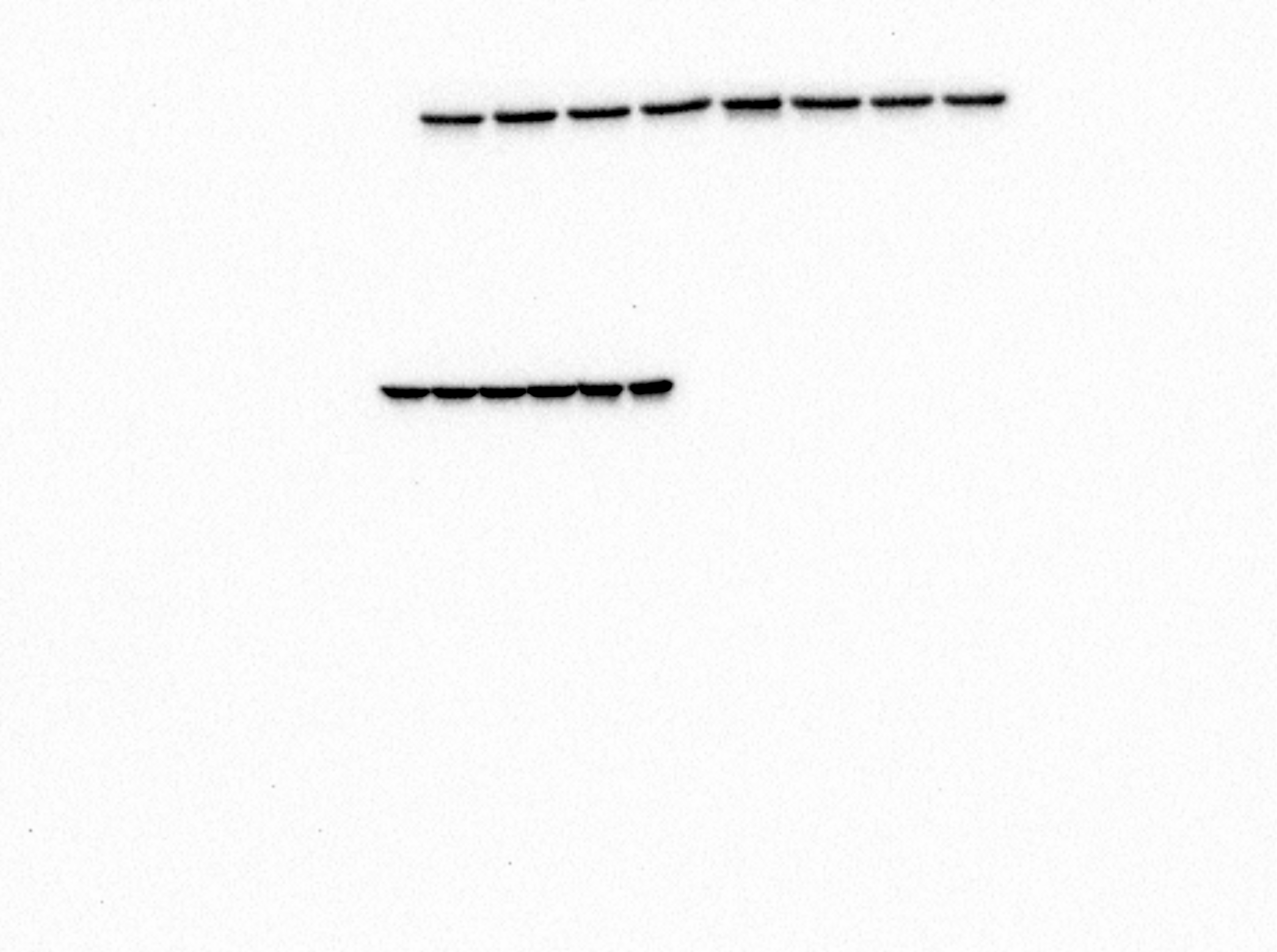

Supplement: Figure 4—source data 1. [file elife-85898-fig4-data1.zip › Figure 4-source data 1/T47D actin lower part.tif]

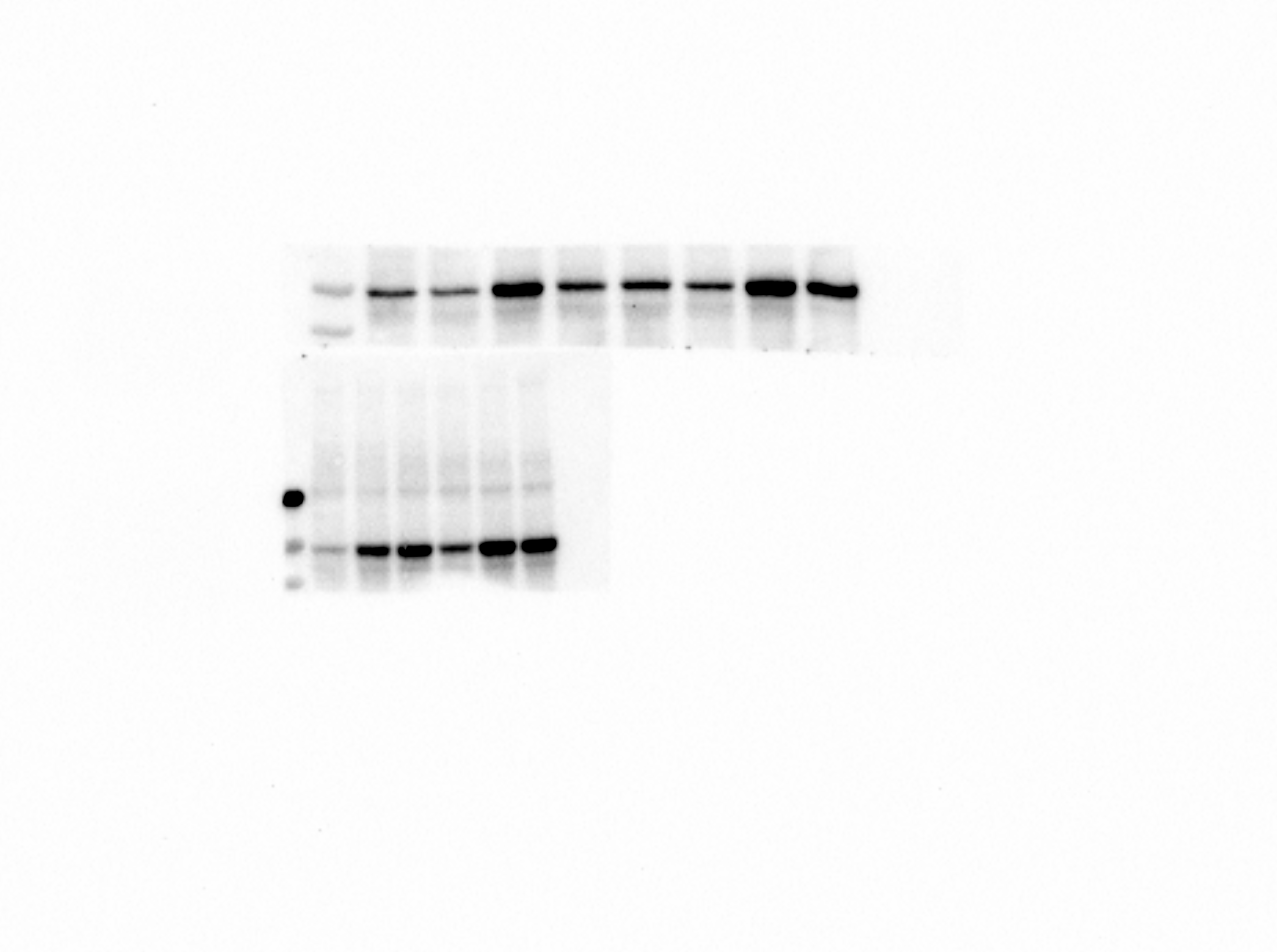

Supplement: Figure 4—source data 1. [file elife-85898-fig4-data1.zip › Figure 4-source data 1/T47D ATF4 lower part.tif]

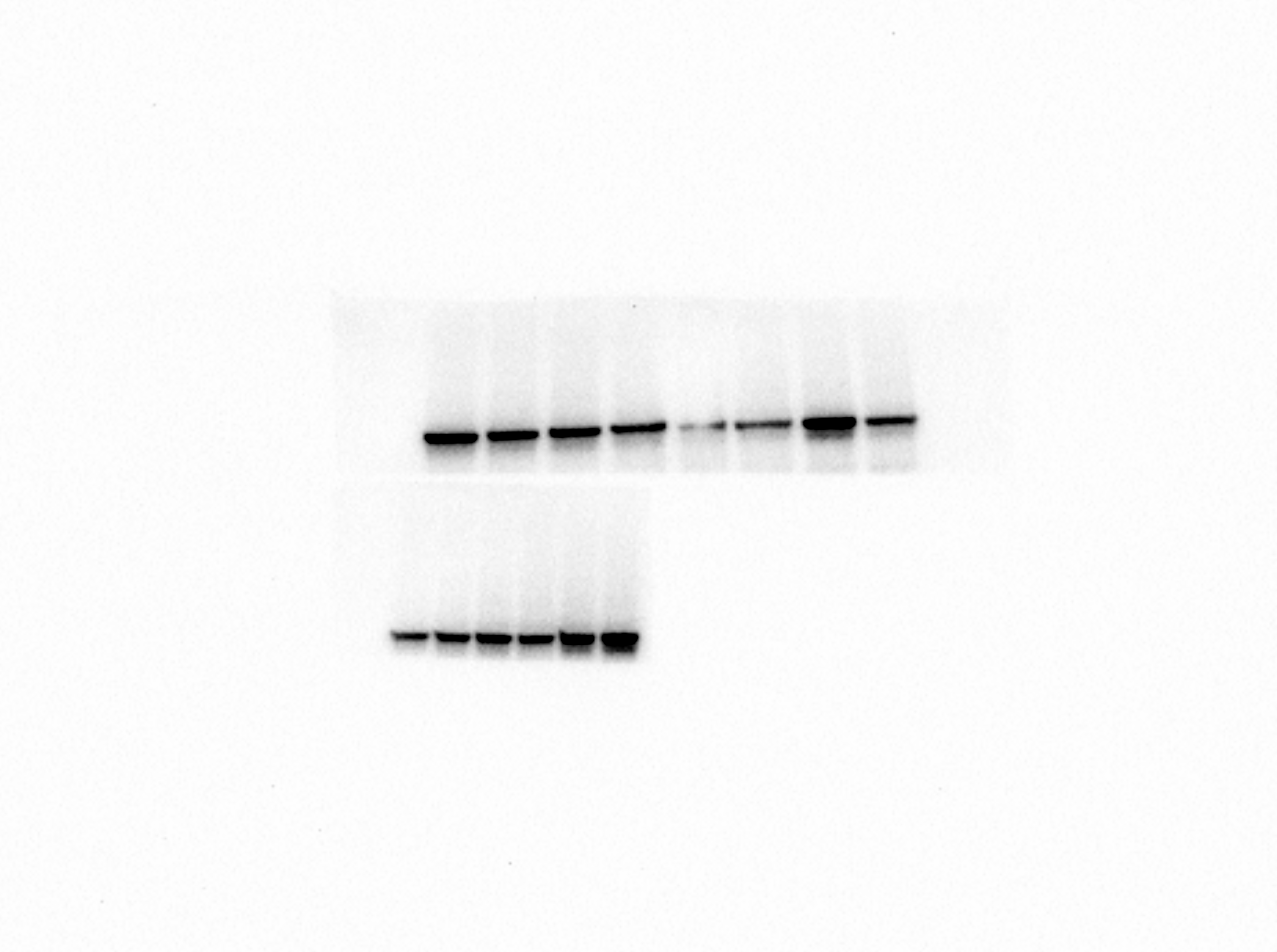

Supplement: Figure 4—source data 1. [file elife-85898-fig4-data1.zip › Figure 4-source data 1/T47D BiP lower part.tif]

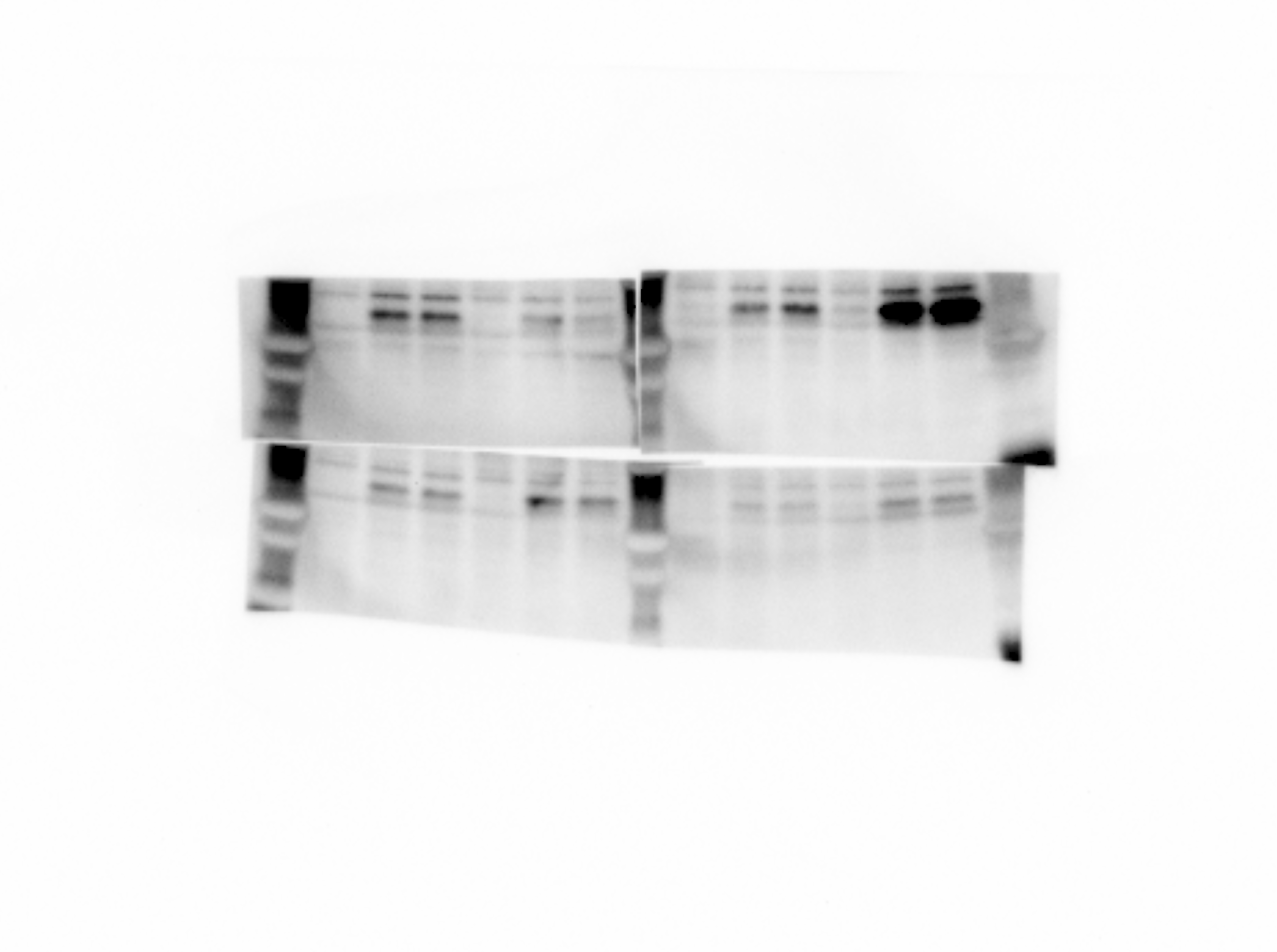

Supplement: Figure 4—source data 1. [file elife-85898-fig4-data1.zip › Figure 4-source data 1/T47D CHOP upper right.tif]

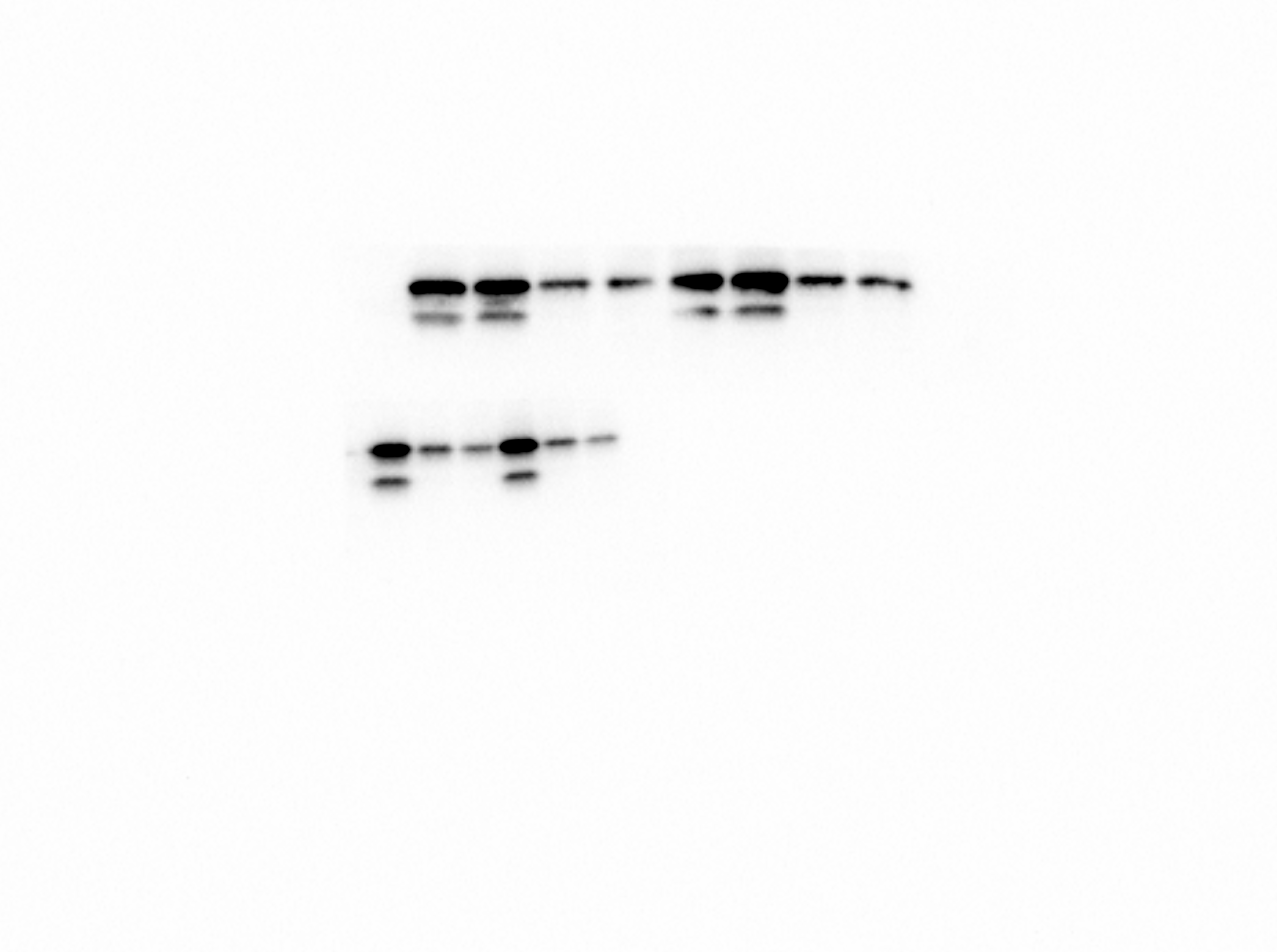

Supplement: Figure 4—source data 1. [file elife-85898-fig4-data1.zip › Figure 4-source data 1/T47D TFAM lower part.tif]

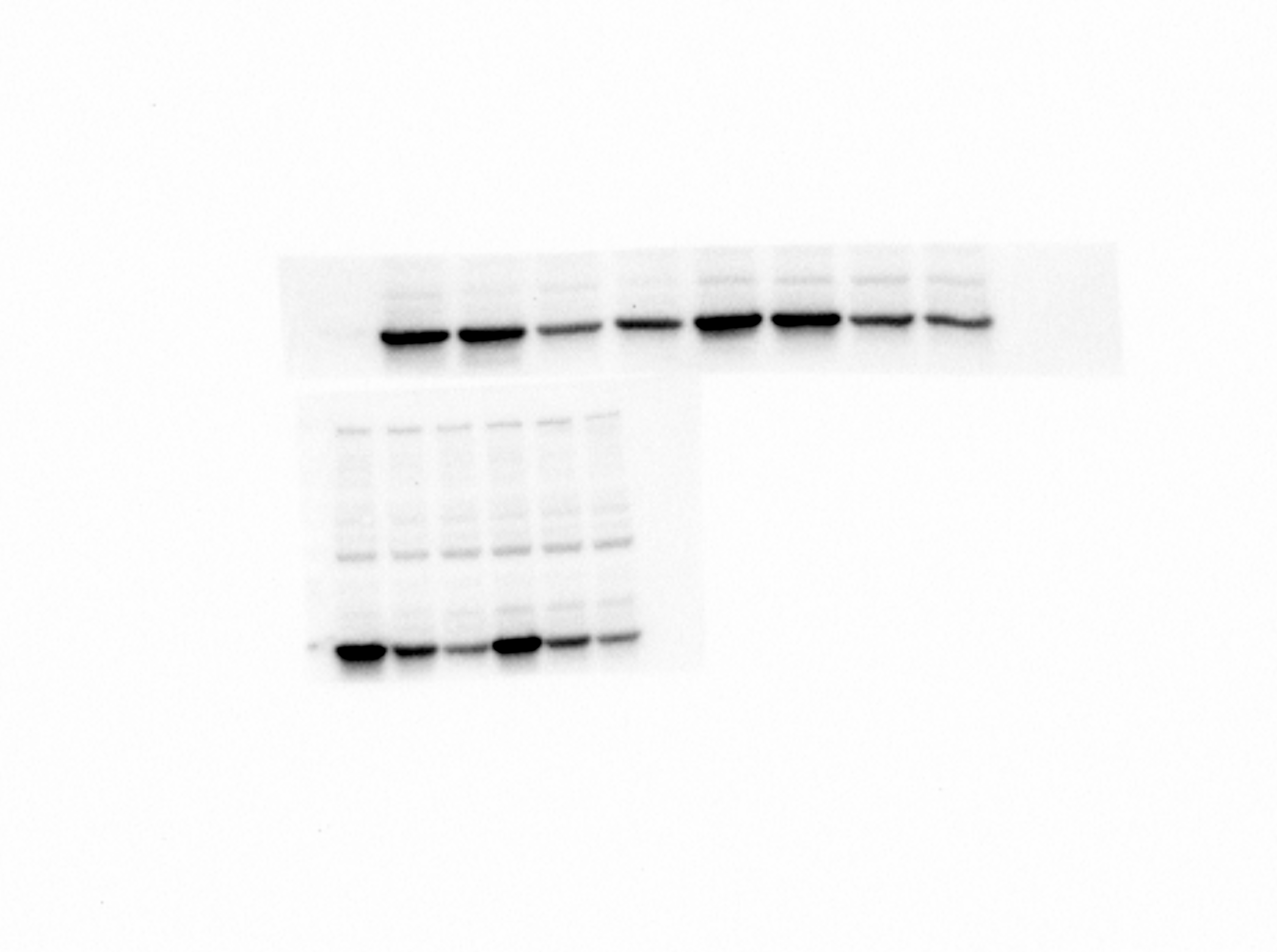

Supplement: Figure 4—source data 1. [file elife-85898-fig4-data1.zip › Figure 4-source data 1/T47D TUFM lower part.tif]

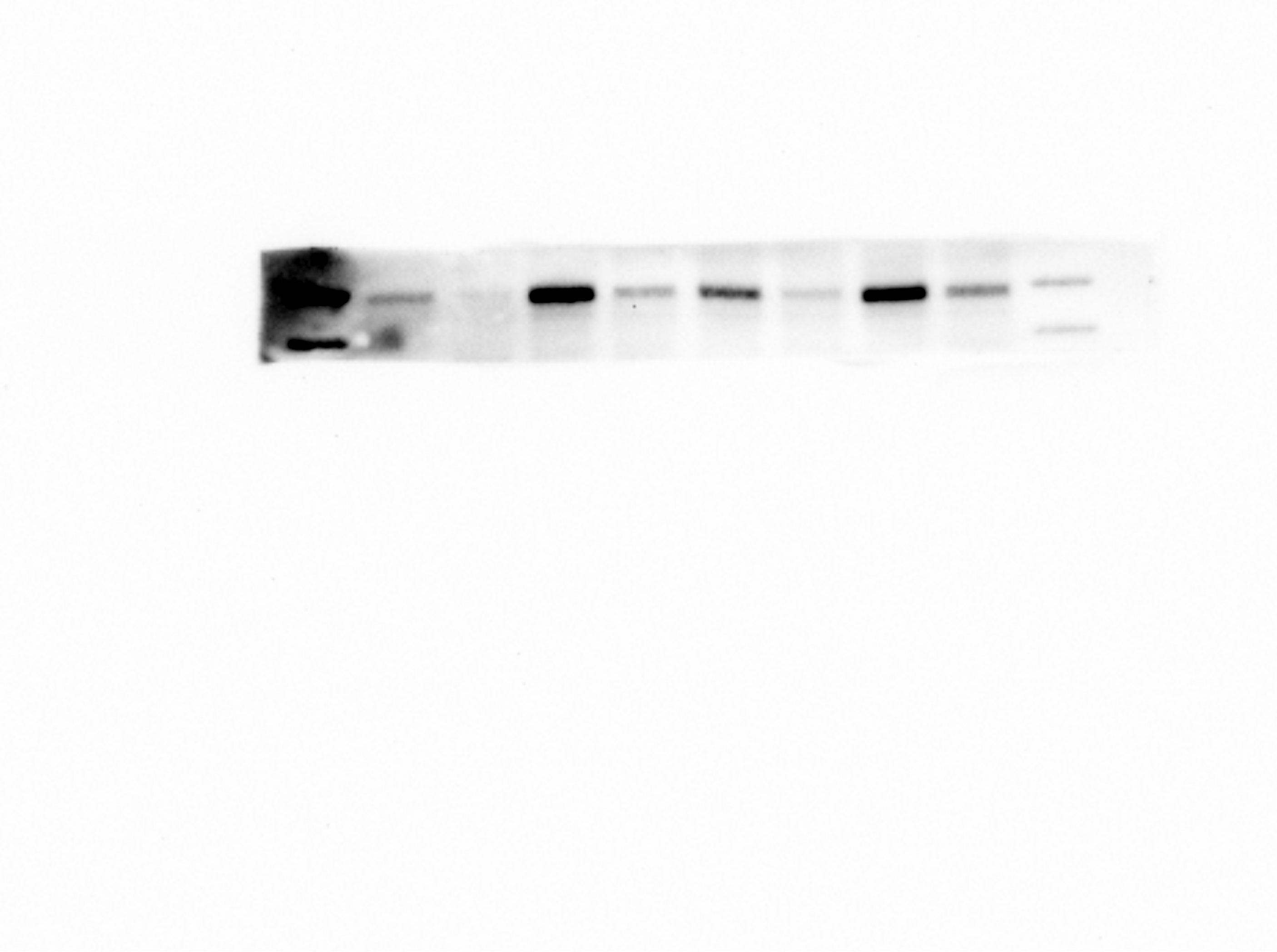

Supplement: Figure 4—source data 2. [file elife-85898-fig4-data2.zip › Figure 4-sourse data 2/CAMA ATF4.tif]

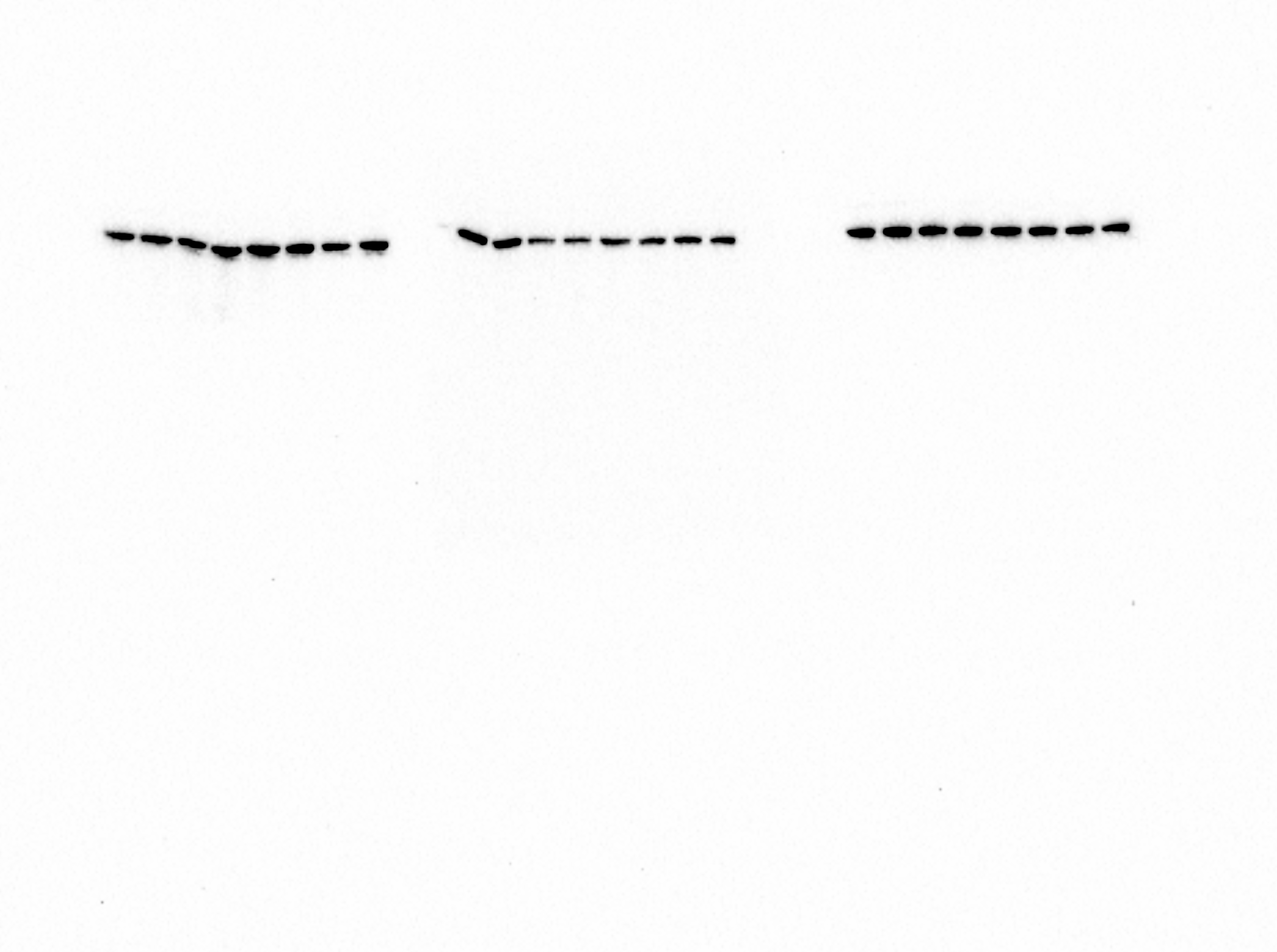

Supplement: Figure 4—source data 2. [file elife-85898-fig4-data2.zip › Figure 4-sourse data 2/CAMA actin OXPHOS left part.tif]

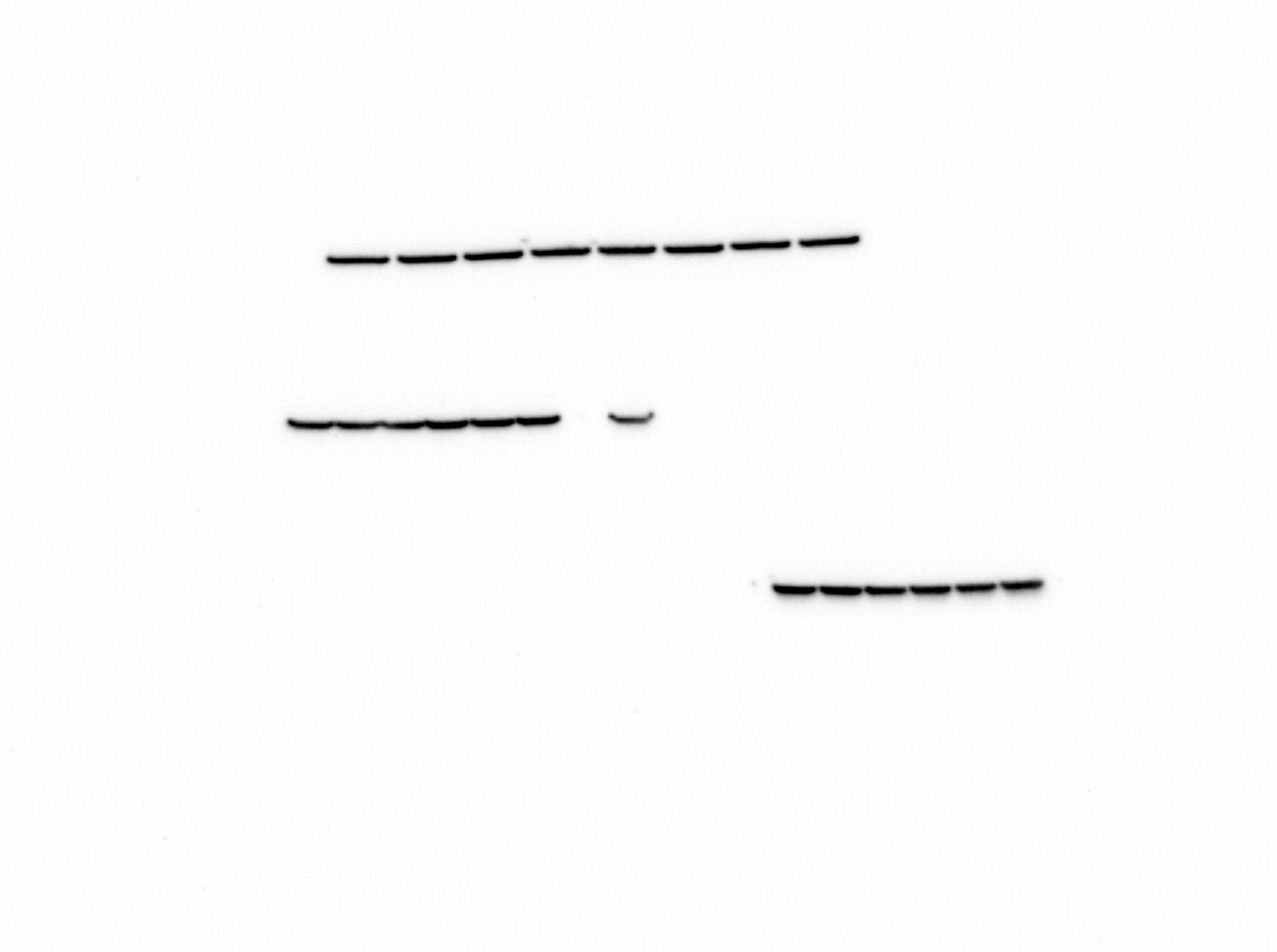

Supplement: Figure 4—source data 2. [file elife-85898-fig4-data2.zip › Figure 4-sourse data 2/CAMA actin upper part.tif]

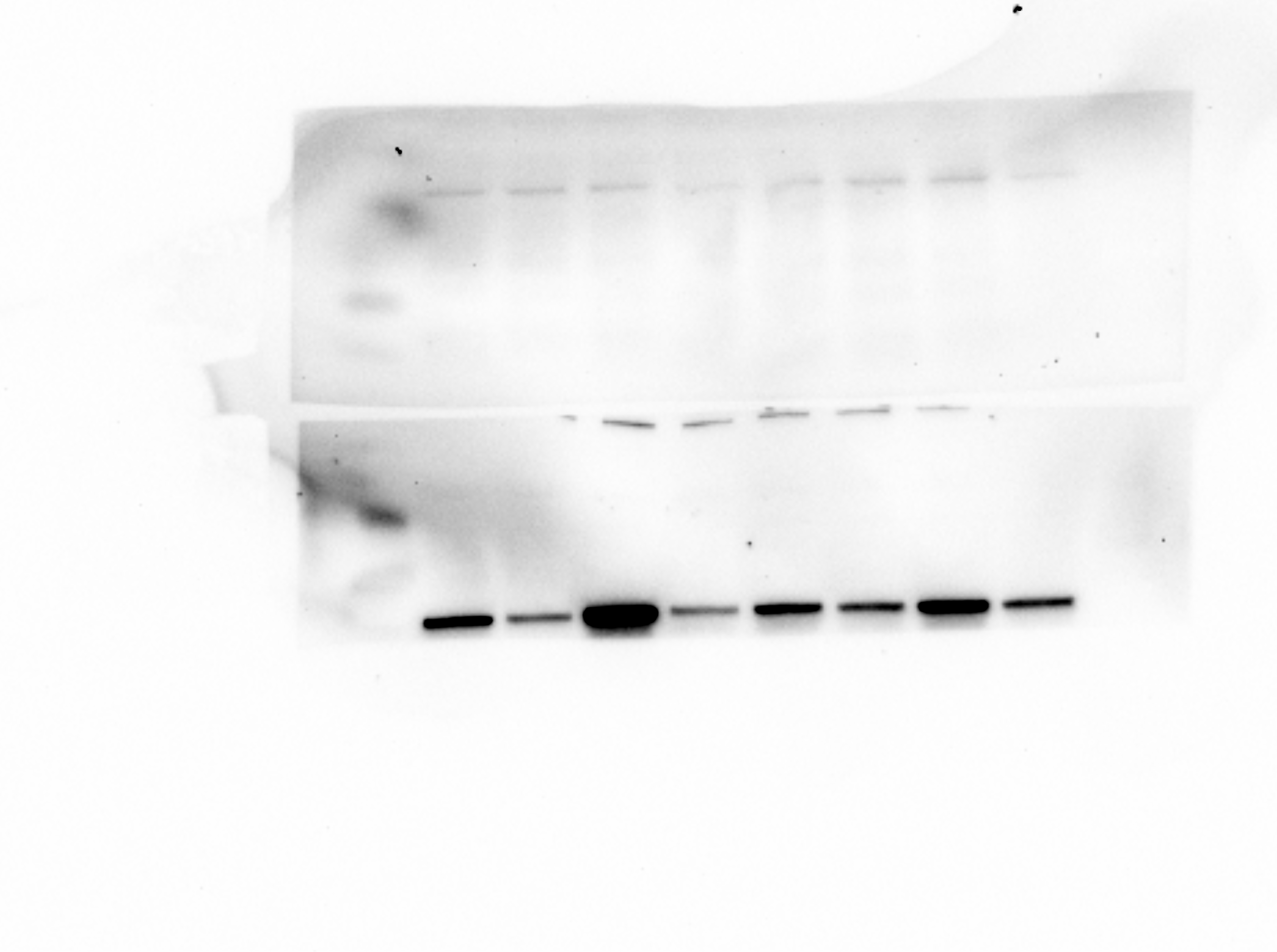

Supplement: Figure 4—source data 2. [file elife-85898-fig4-data2.zip › Figure 4-sourse data 2/CAMA BiP lower part.tif]

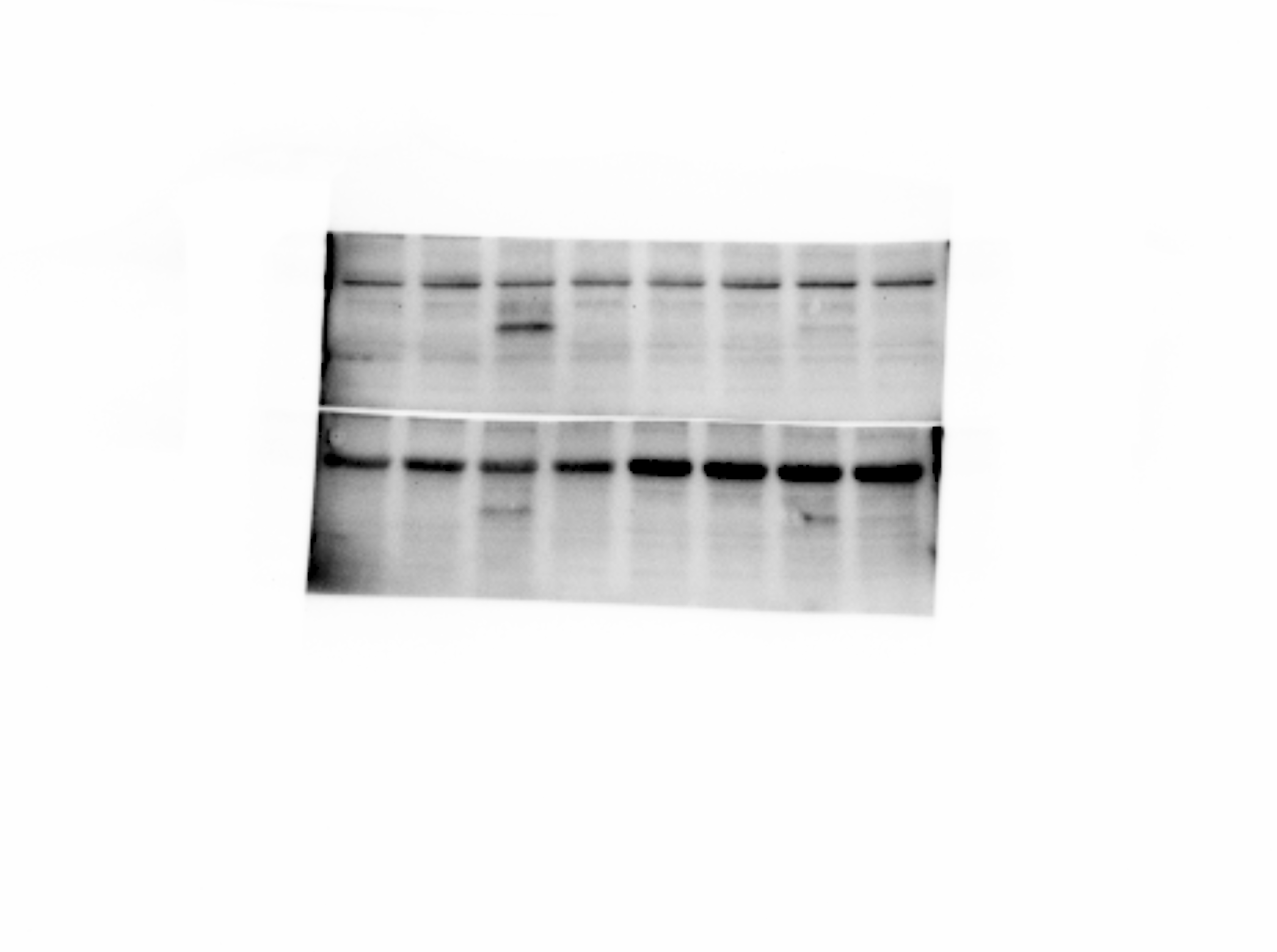

Supplement: Figure 4—source data 2. [file elife-85898-fig4-data2.zip › Figure 4-sourse data 2/CAMA CHOP upper part.tif]

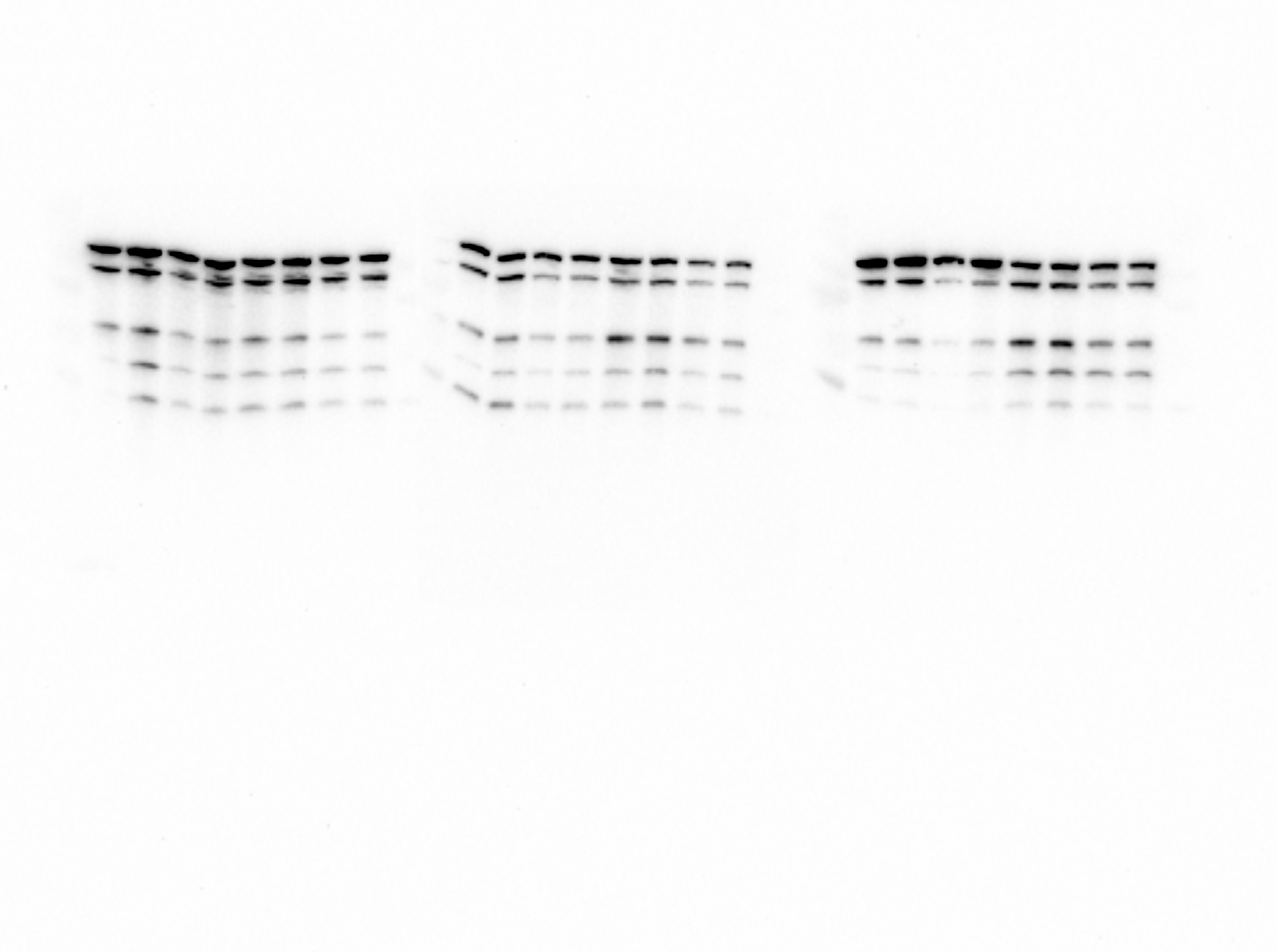

Supplement: Figure 4—source data 2. [file elife-85898-fig4-data2.zip › Figure 4-sourse data 2/CAMA OXPHOS left part.tif]

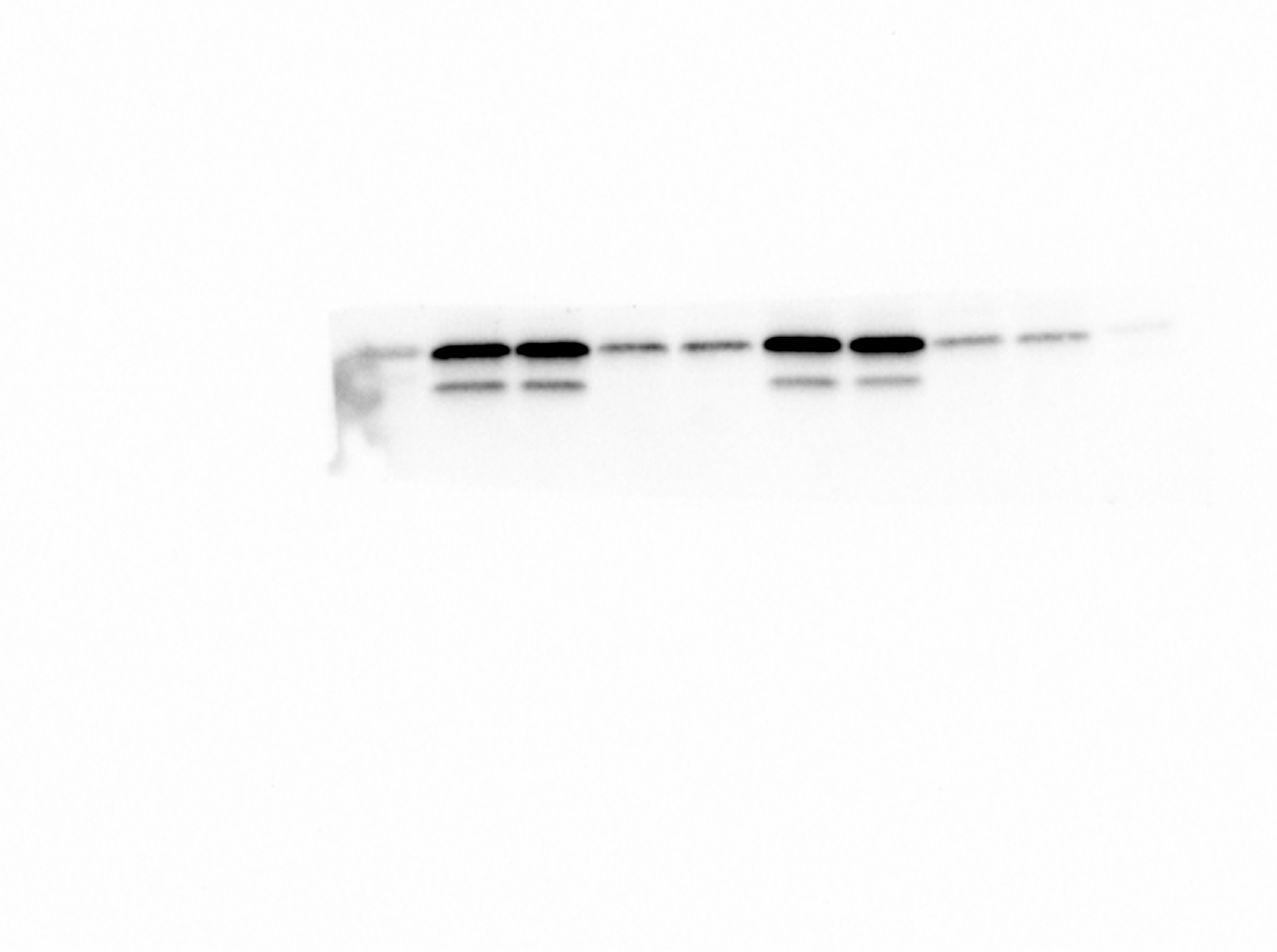

Supplement: Figure 4—source data 2. [file elife-85898-fig4-data2.zip › Figure 4-sourse data 2/CAMA TFAM.tif]

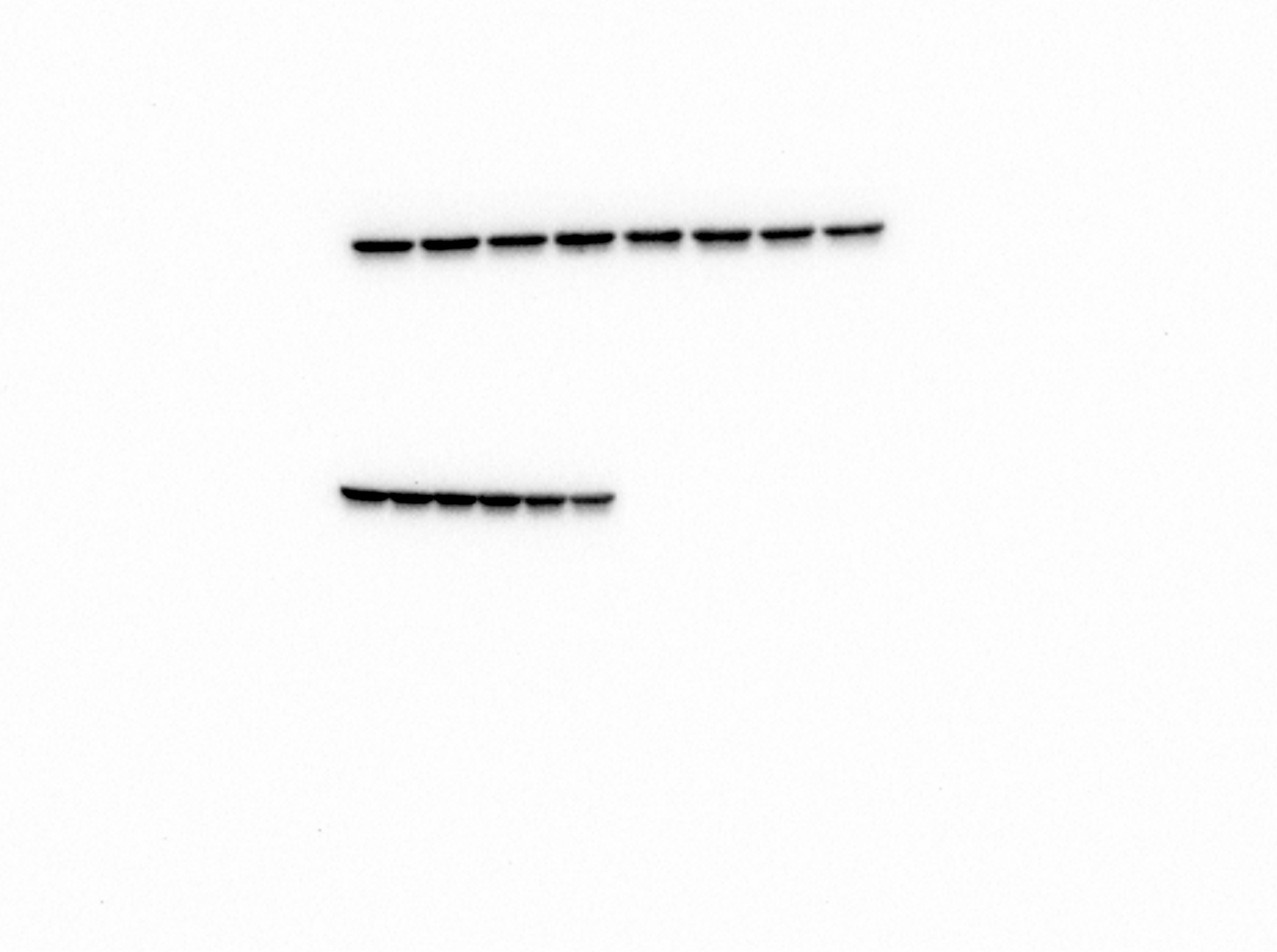

Supplement: Figure 4—source data 2. [file elife-85898-fig4-data2.zip › Figure 4-sourse data 2/MCF7 actin upper part.tif]

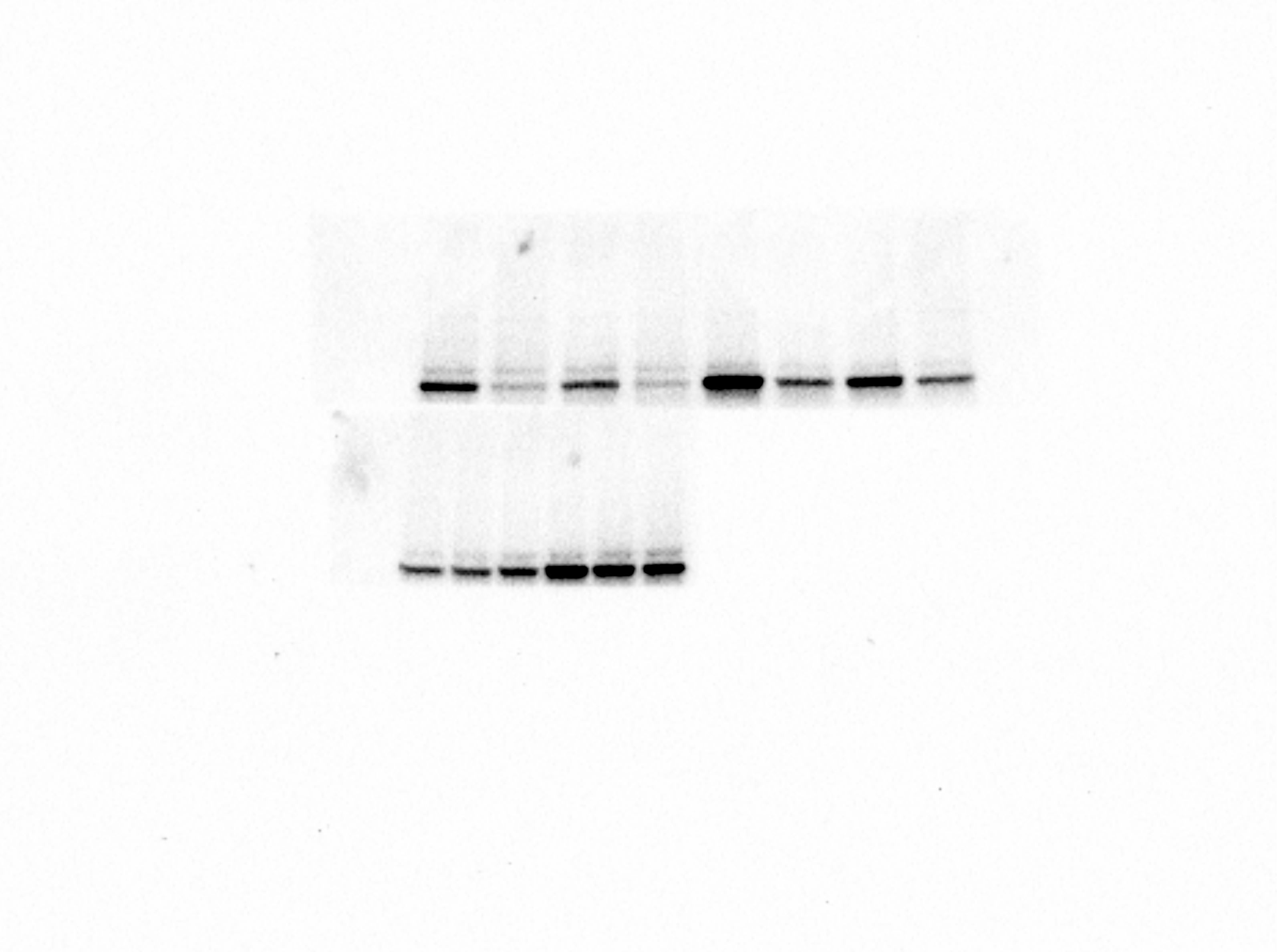

Supplement: Figure 4—source data 2. [file elife-85898-fig4-data2.zip › Figure 4-sourse data 2/MCF7 BiP upper part.tif]

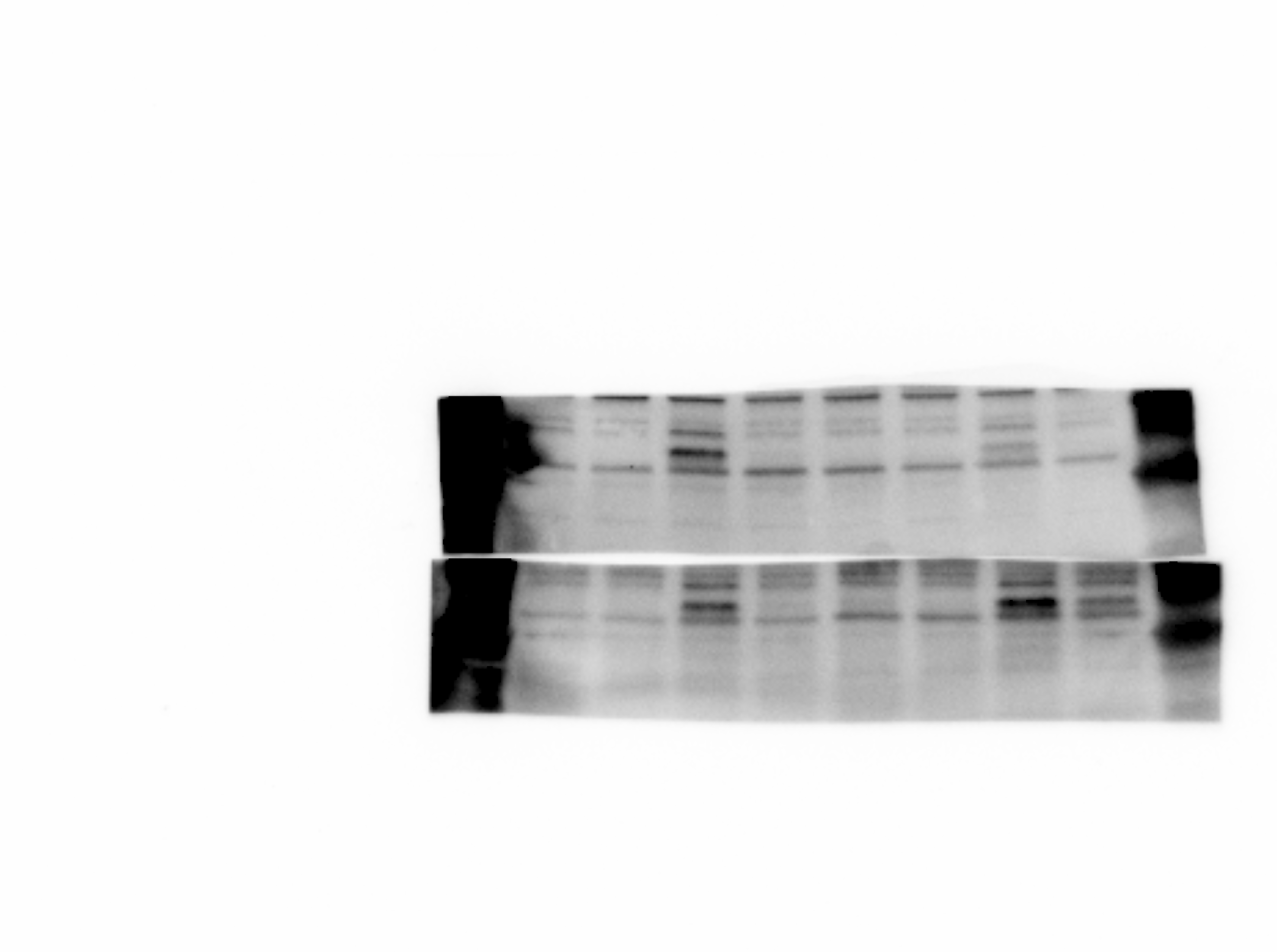

Supplement: Figure 4—source data 2. [file elife-85898-fig4-data2.zip › Figure 4-sourse data 2/MCF7 CHOP lower part.tif]

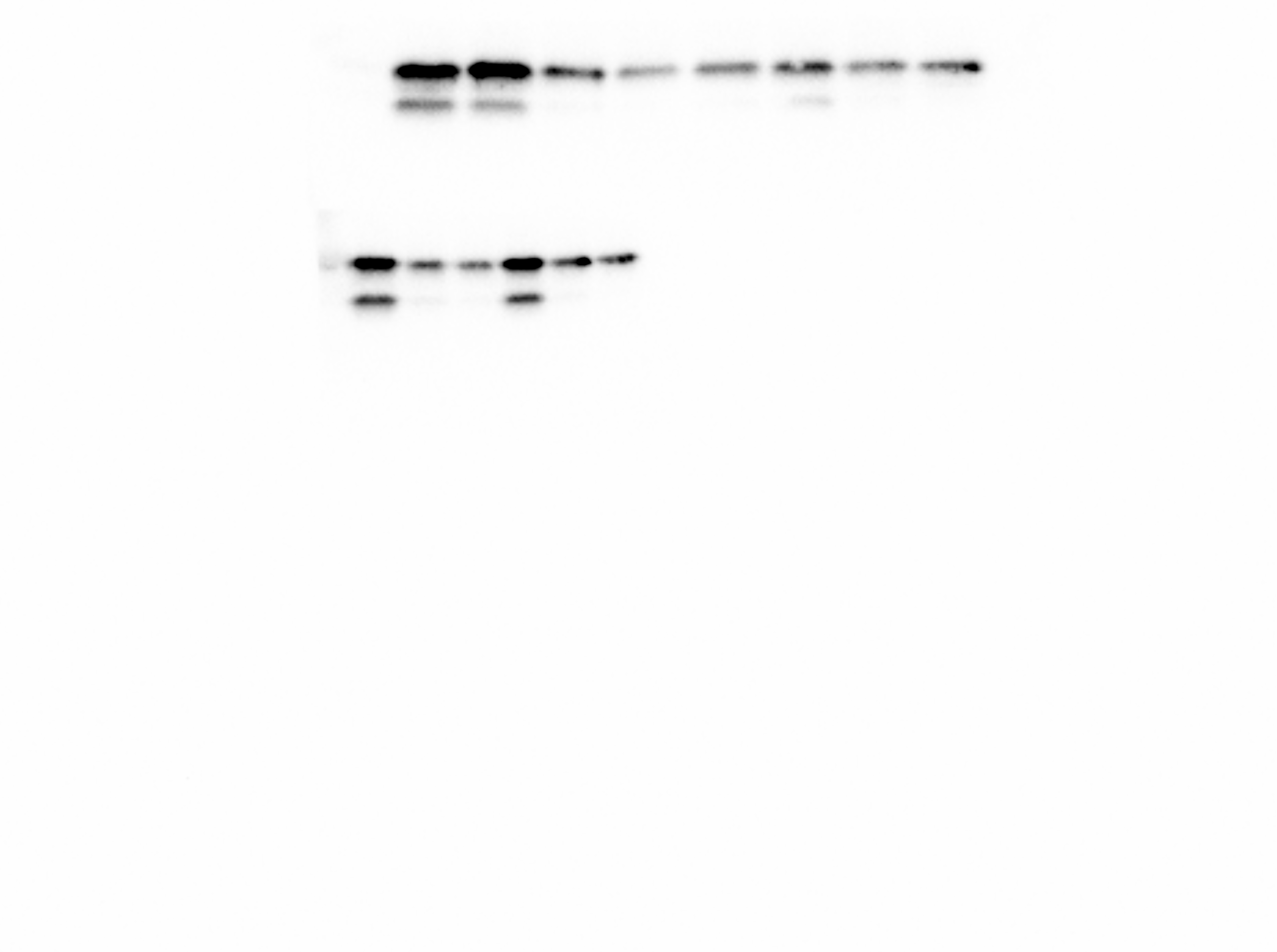

Supplement: Figure 4—source data 2. [file elife-85898-fig4-data2.zip › Figure 4-sourse data 2/MCF7 TFAM upper part.tif]

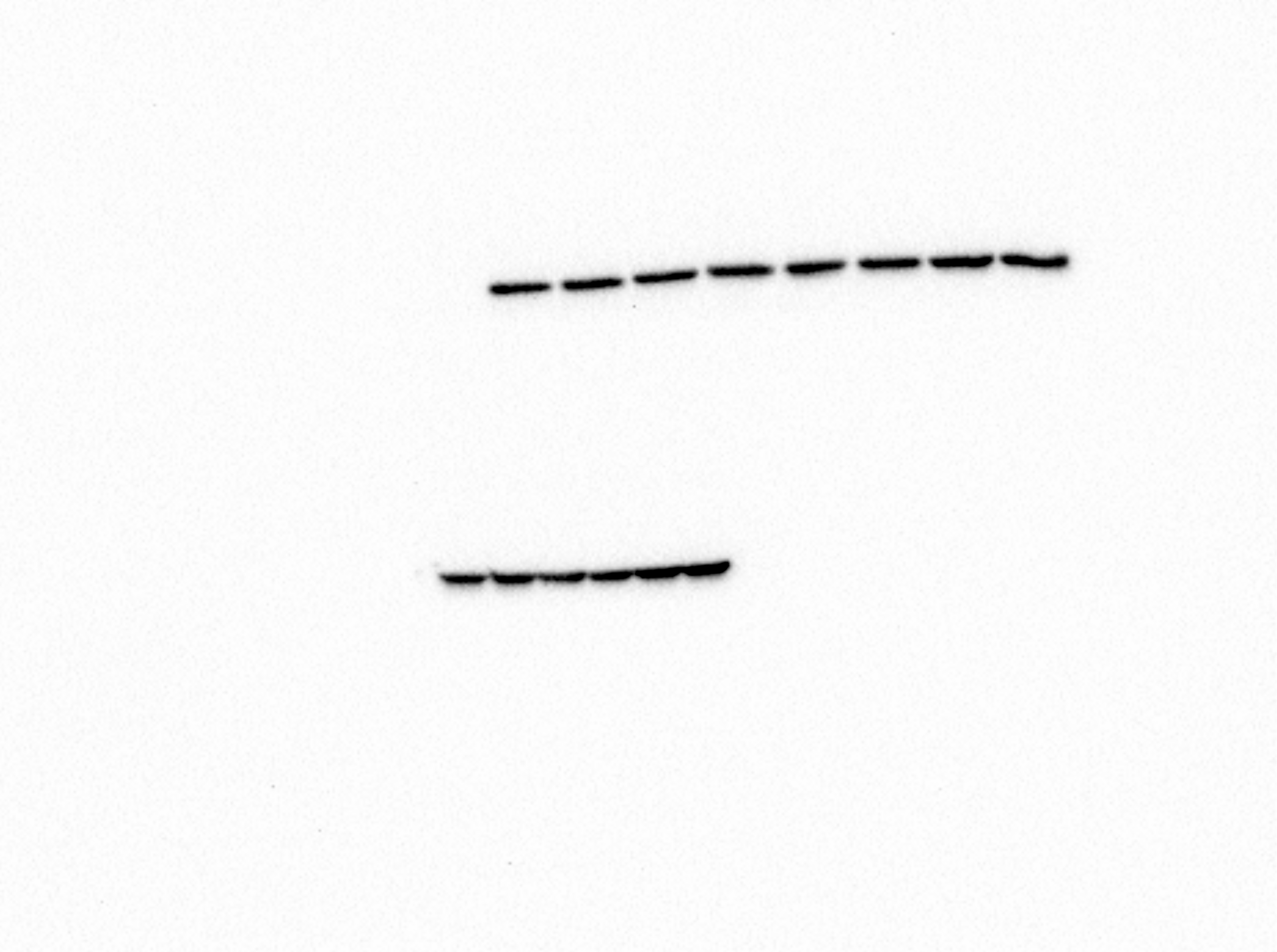

Supplement: Figure 4—source data 2. [file elife-85898-fig4-data2.zip › Figure 4-sourse data 2/T47D actin upper part.tif]

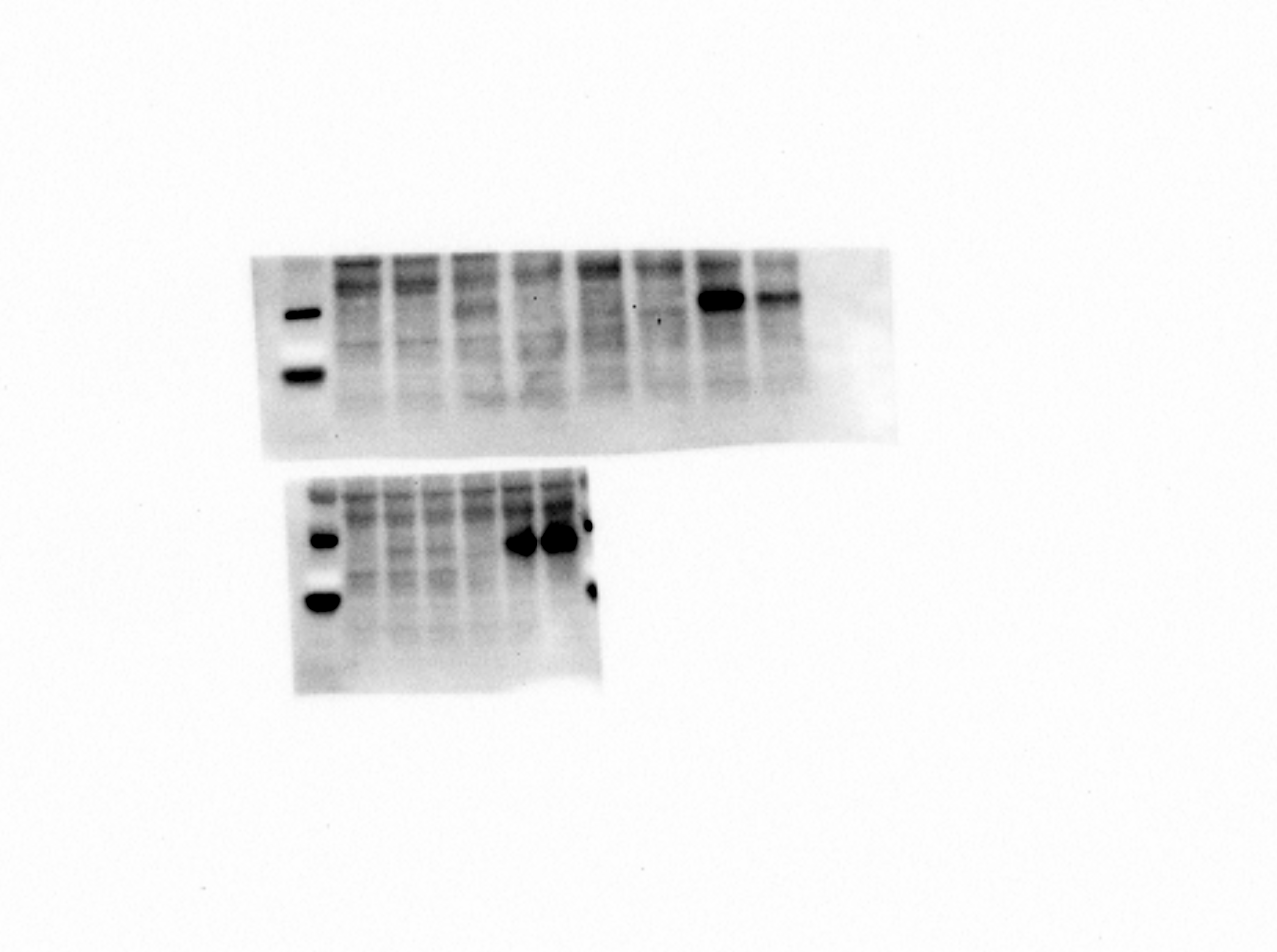

Supplement: Figure 4—source data 2. [file elife-85898-fig4-data2.zip › Figure 4-sourse data 2/T47D CHOP upper part.tif]

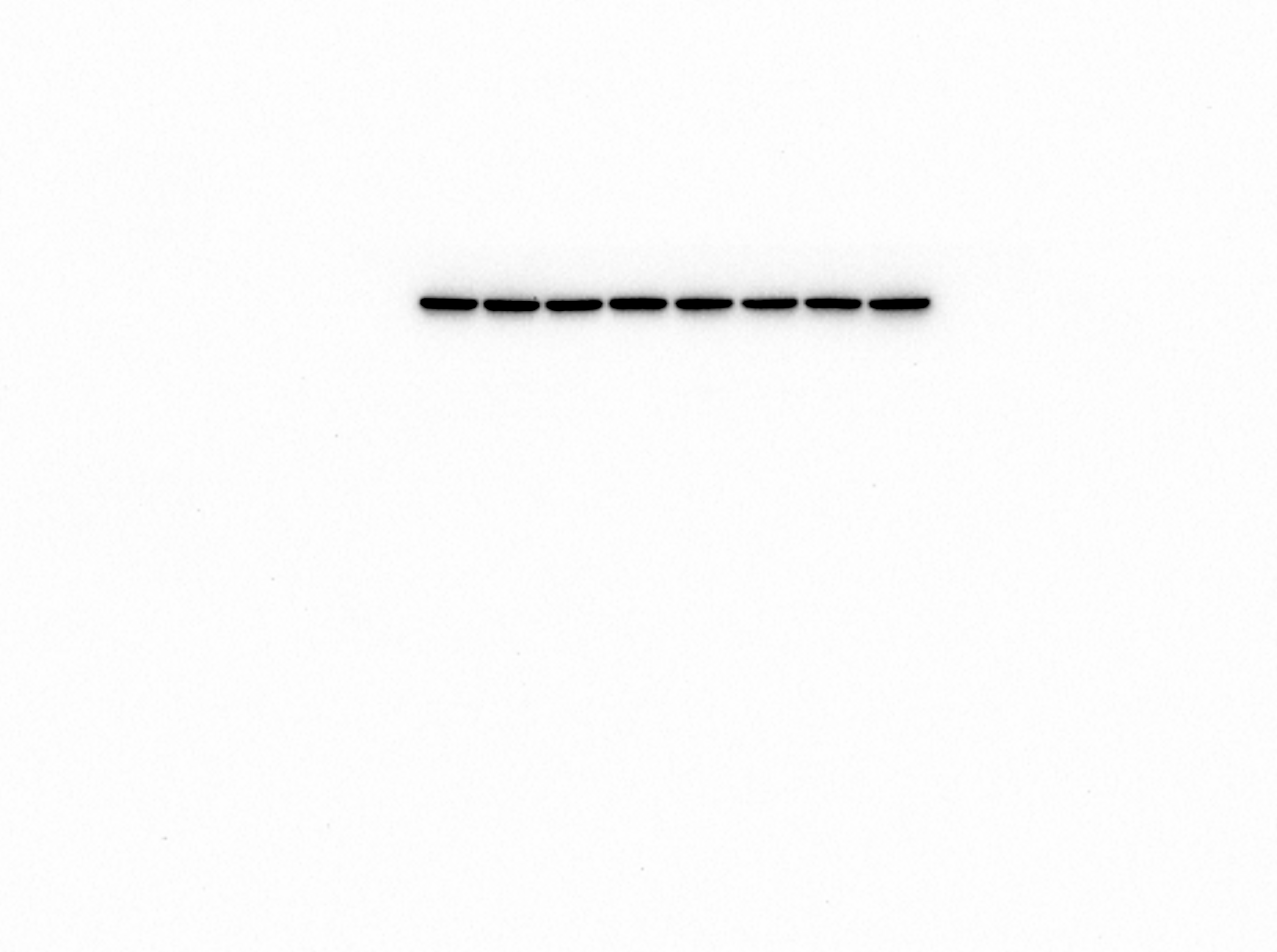

Supplement: Figure 4—figure supplement 1—source data 1. [file elife-85898-fig4-figsupp1-data1.zip › Figure 4-figure supplement 1-source data 1 - Copy/CAMA actin.tif]

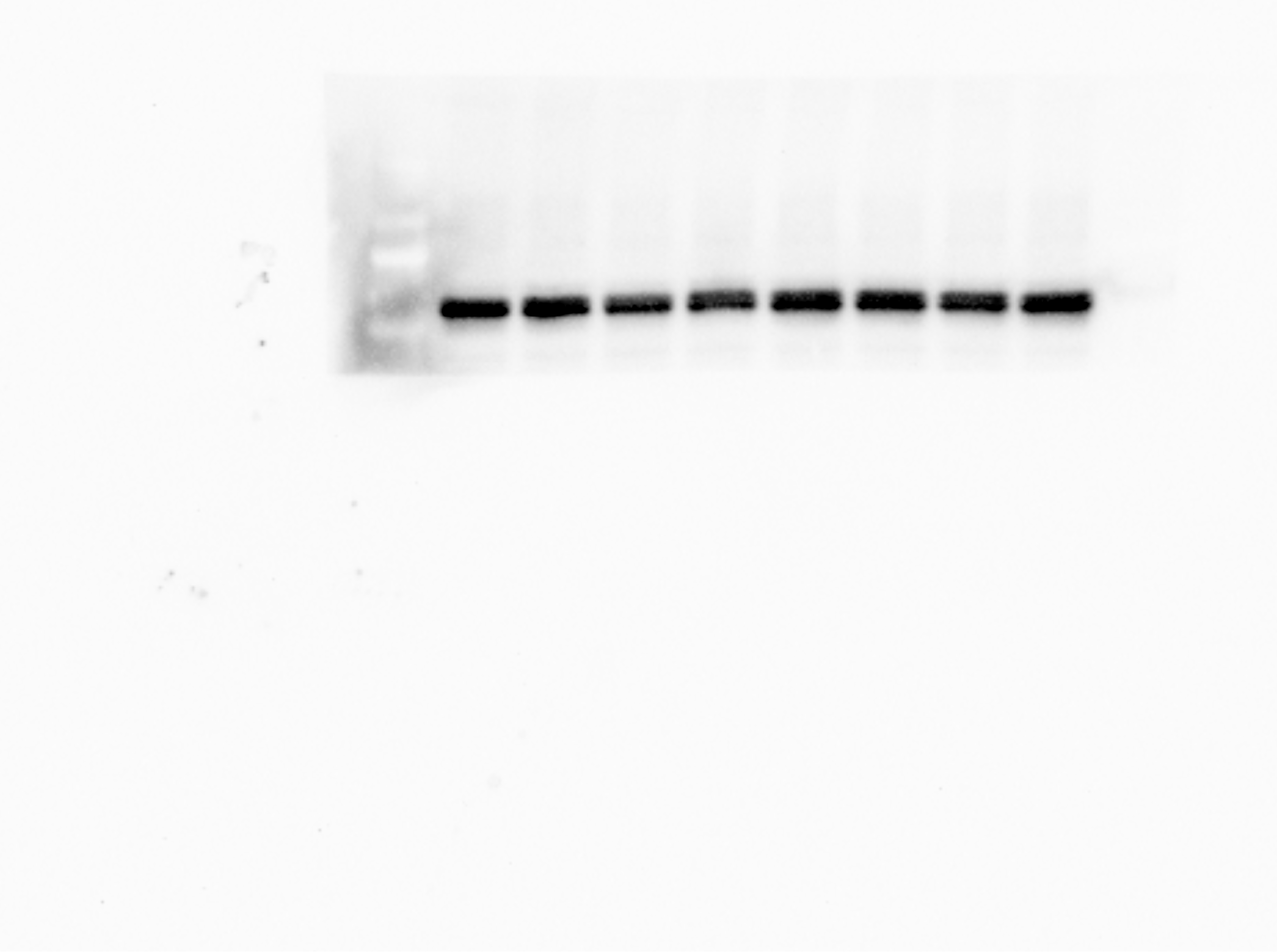

Supplement: Figure 4—figure supplement 1—source data 1. [file elife-85898-fig4-figsupp1-data1.zip › Figure 4-figure supplement 1-source data 1 - Copy/CAMA AKT.tif]

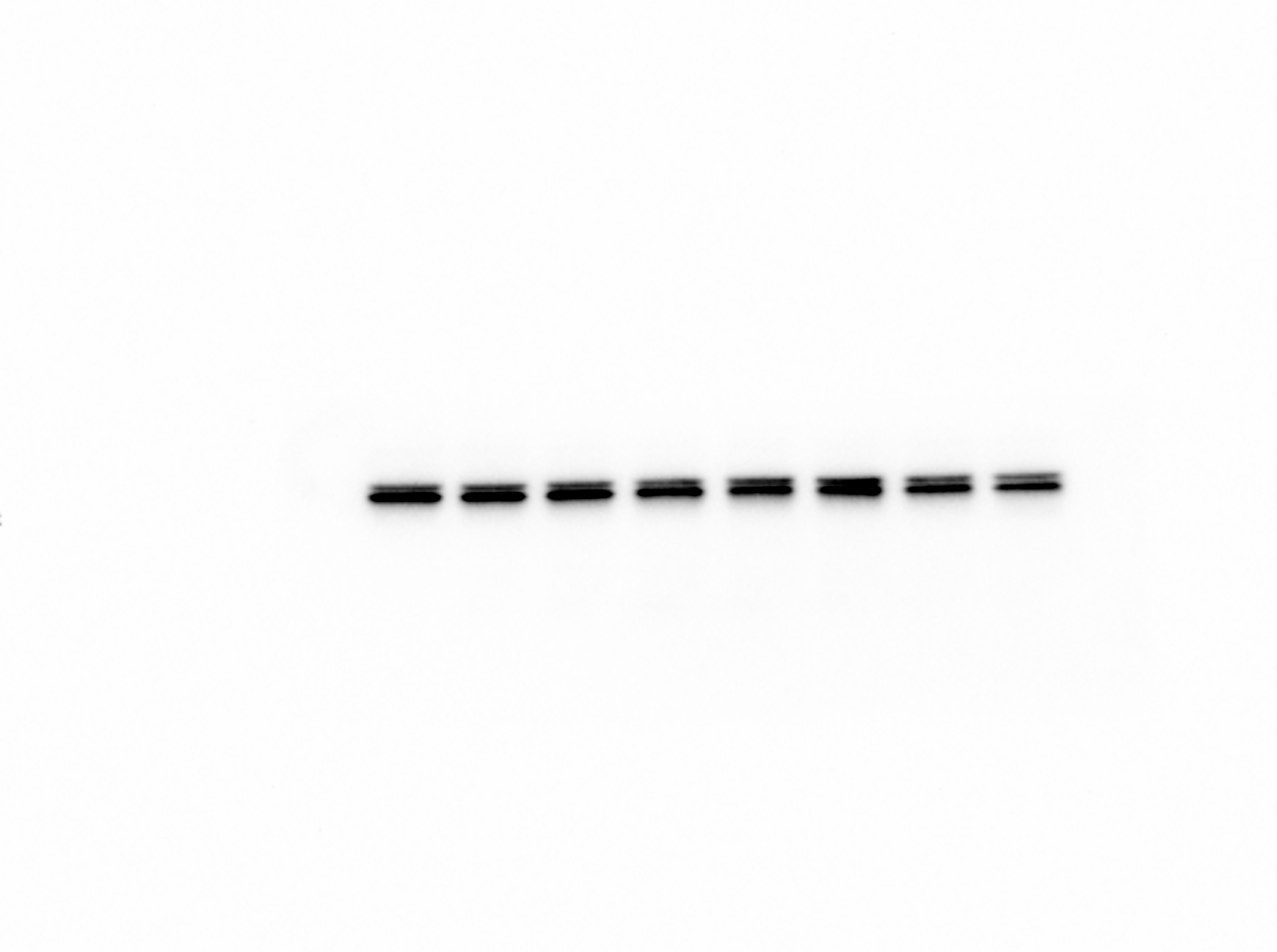

Supplement: Figure 4—figure supplement 1—source data 1. [file elife-85898-fig4-figsupp1-data1.zip › Figure 4-figure supplement 1-source data 1 - Copy/CAMA ERK.tif]

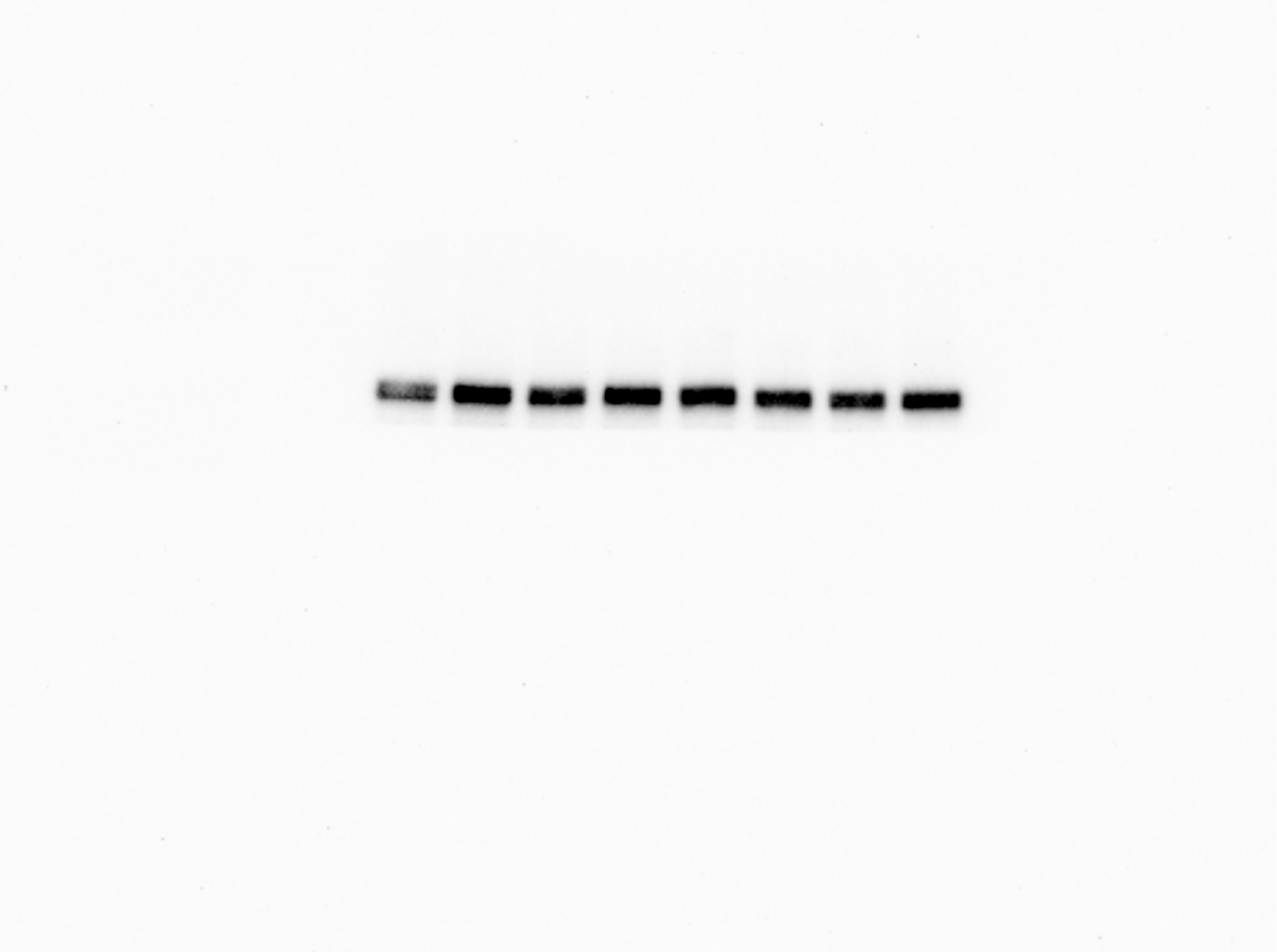

Supplement: Figure 4—figure supplement 1—source data 1. [file elife-85898-fig4-figsupp1-data1.zip › Figure 4-figure supplement 1-source data 1 - Copy/CAMA FOXA3A.tif]

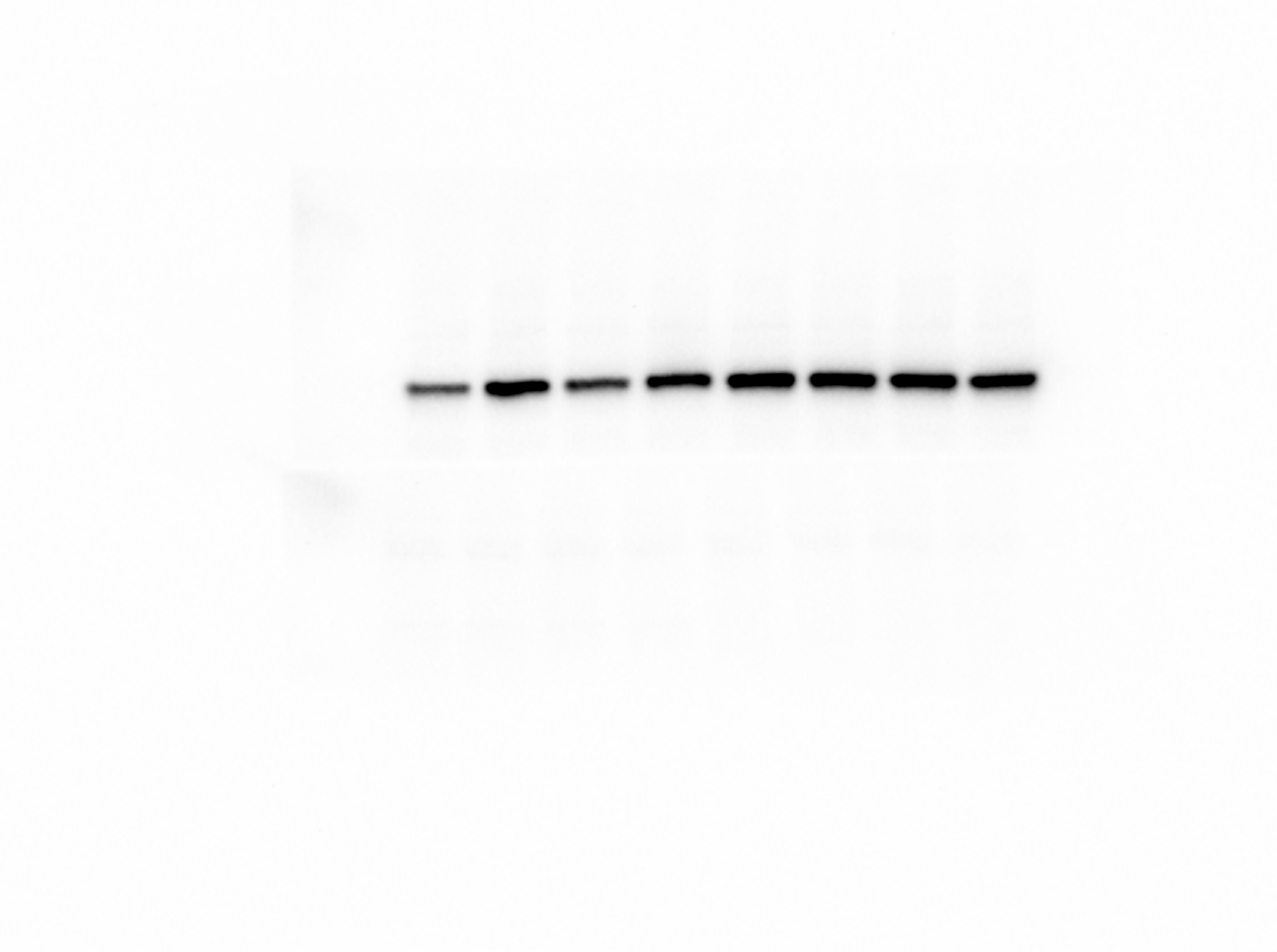

Supplement: Figure 4—figure supplement 1—source data 1. [file elife-85898-fig4-figsupp1-data1.zip › Figure 4-figure supplement 1-source data 1 - Copy/CAMA pAKT.tif]

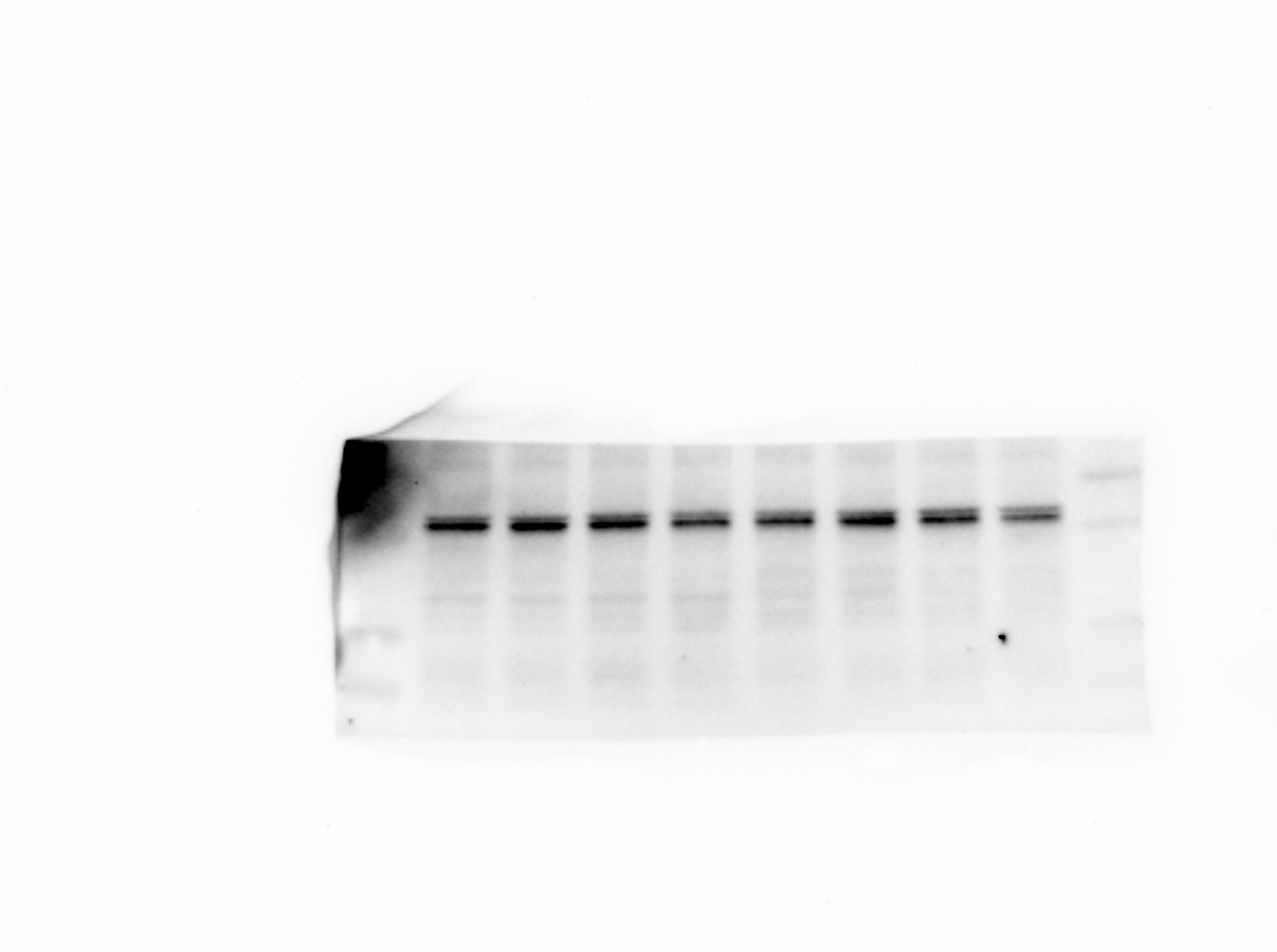

Supplement: Figure 4—figure supplement 1—source data 1. [file elife-85898-fig4-figsupp1-data1.zip › Figure 4-figure supplement 1-source data 1 - Copy/CAMA pERK.tif]

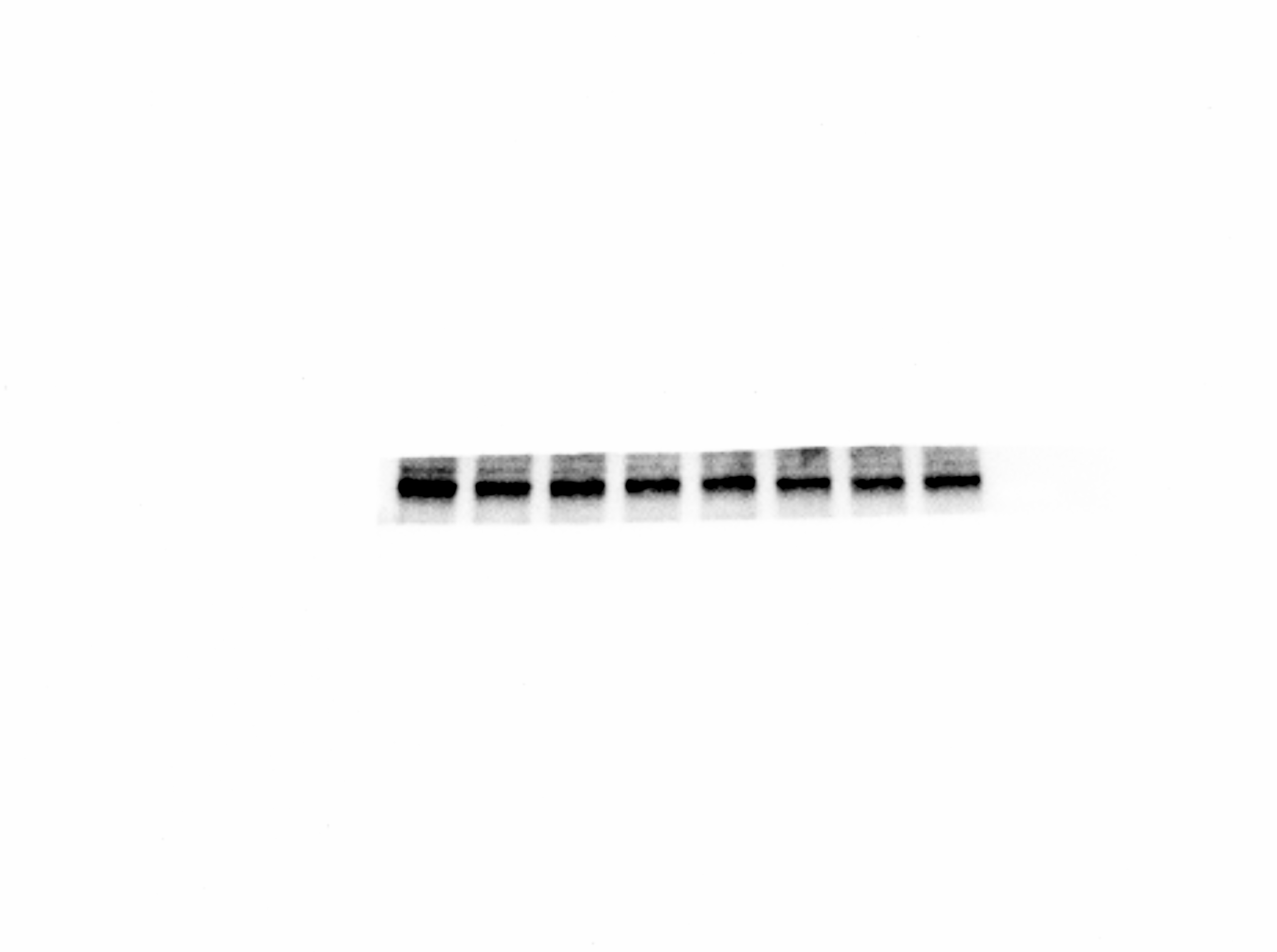

Supplement: Figure 4—figure supplement 1—source data 1. [file elife-85898-fig4-figsupp1-data1.zip › Figure 4-figure supplement 1-source data 1 - Copy/CAMA pFOXA3A.tif]

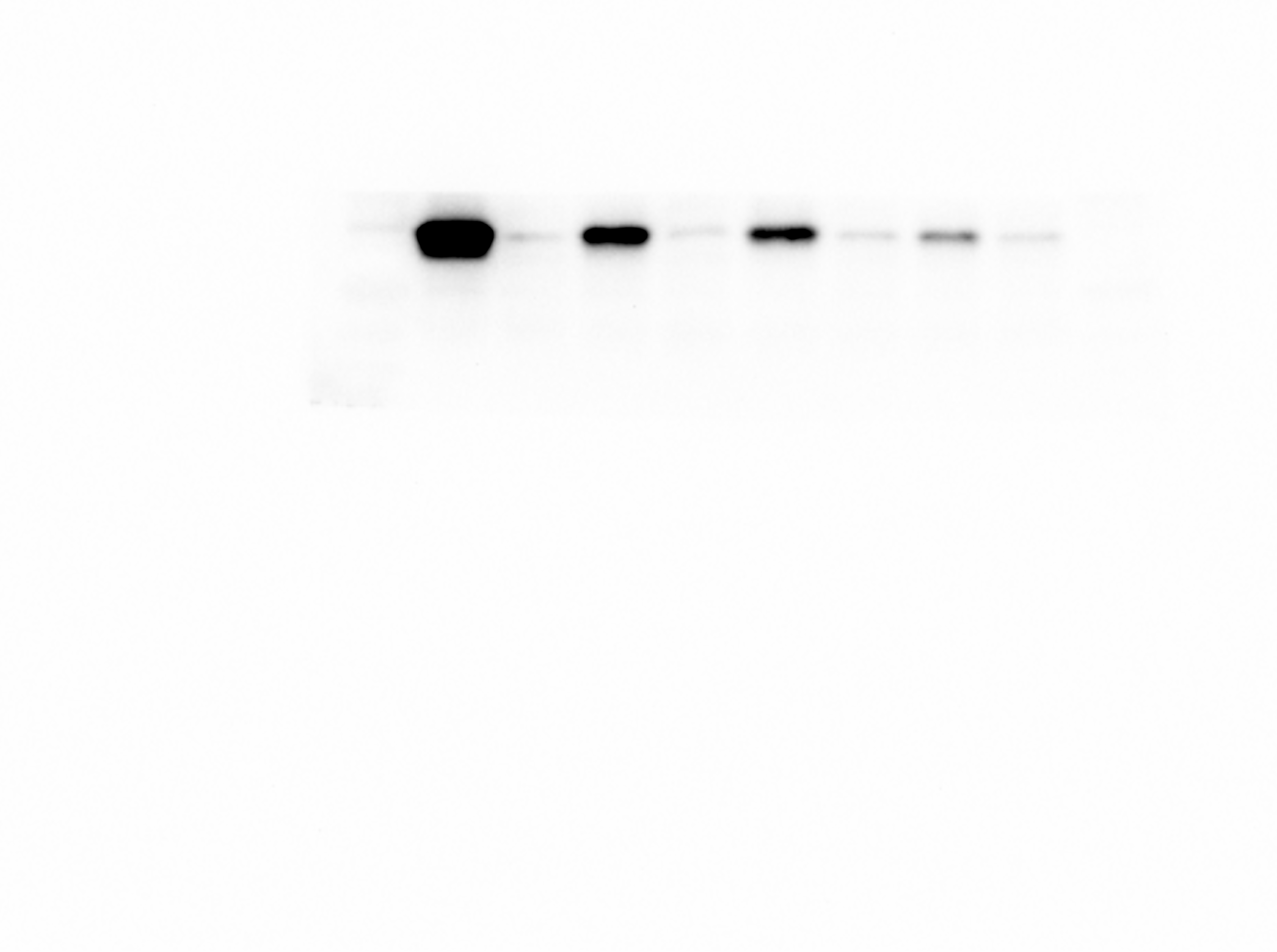

Supplement: Figure 4—figure supplement 1—source data 1. [file elife-85898-fig4-figsupp1-data1.zip › Figure 4-figure supplement 1-source data 1 - Copy/CAMA pS6.tif]

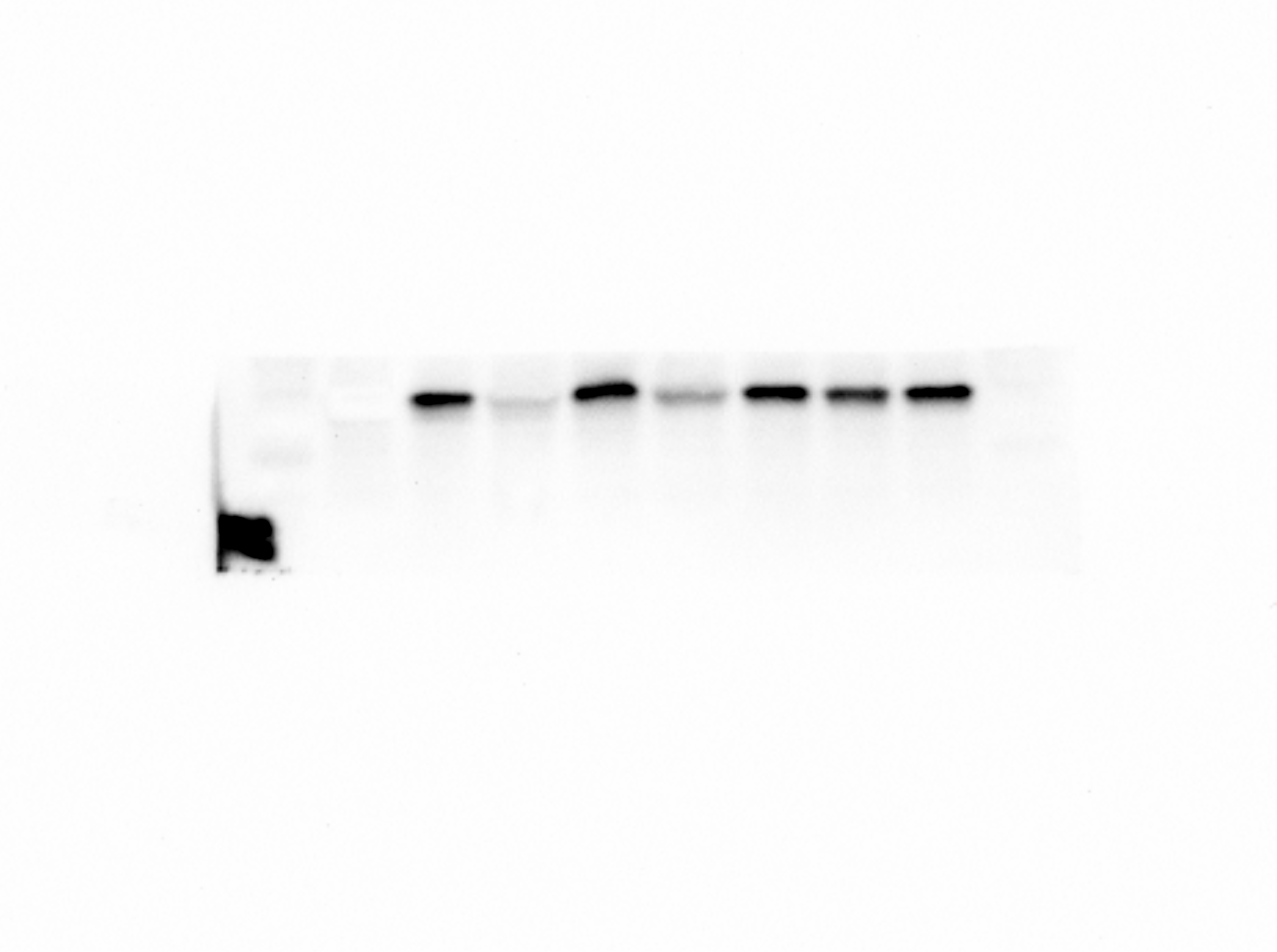

Supplement: Figure 4—figure supplement 1—source data 1. [file elife-85898-fig4-figsupp1-data1.zip › Figure 4-figure supplement 1-source data 1 - Copy/CAMA S6.tif]

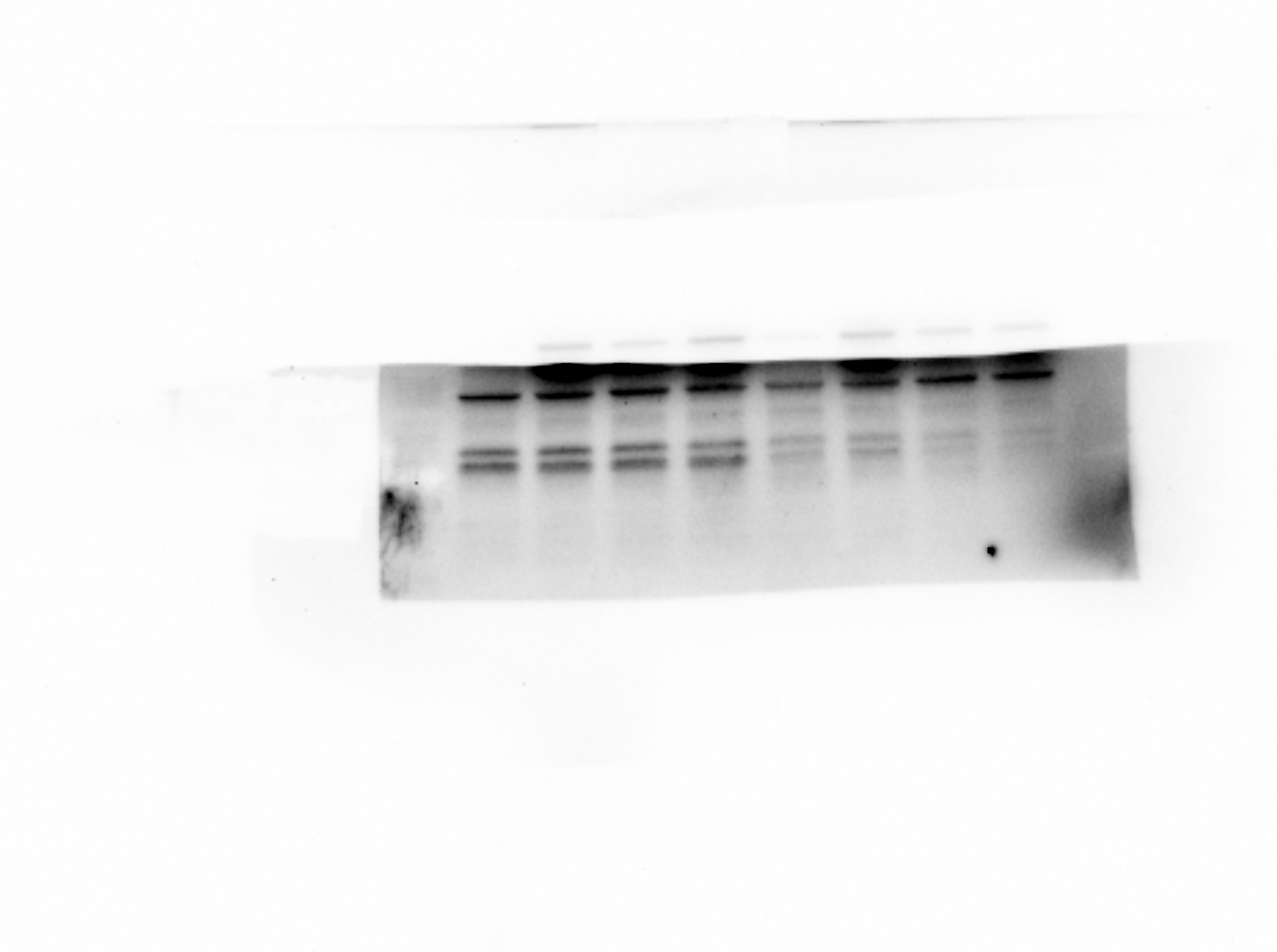

Supplement: Figure 4—figure supplement 1—source data 1. [file elife-85898-fig4-figsupp1-data1.zip › Figure 4-figure supplement 1-source data 1 - Copy/CAMA TRAIL.tif]

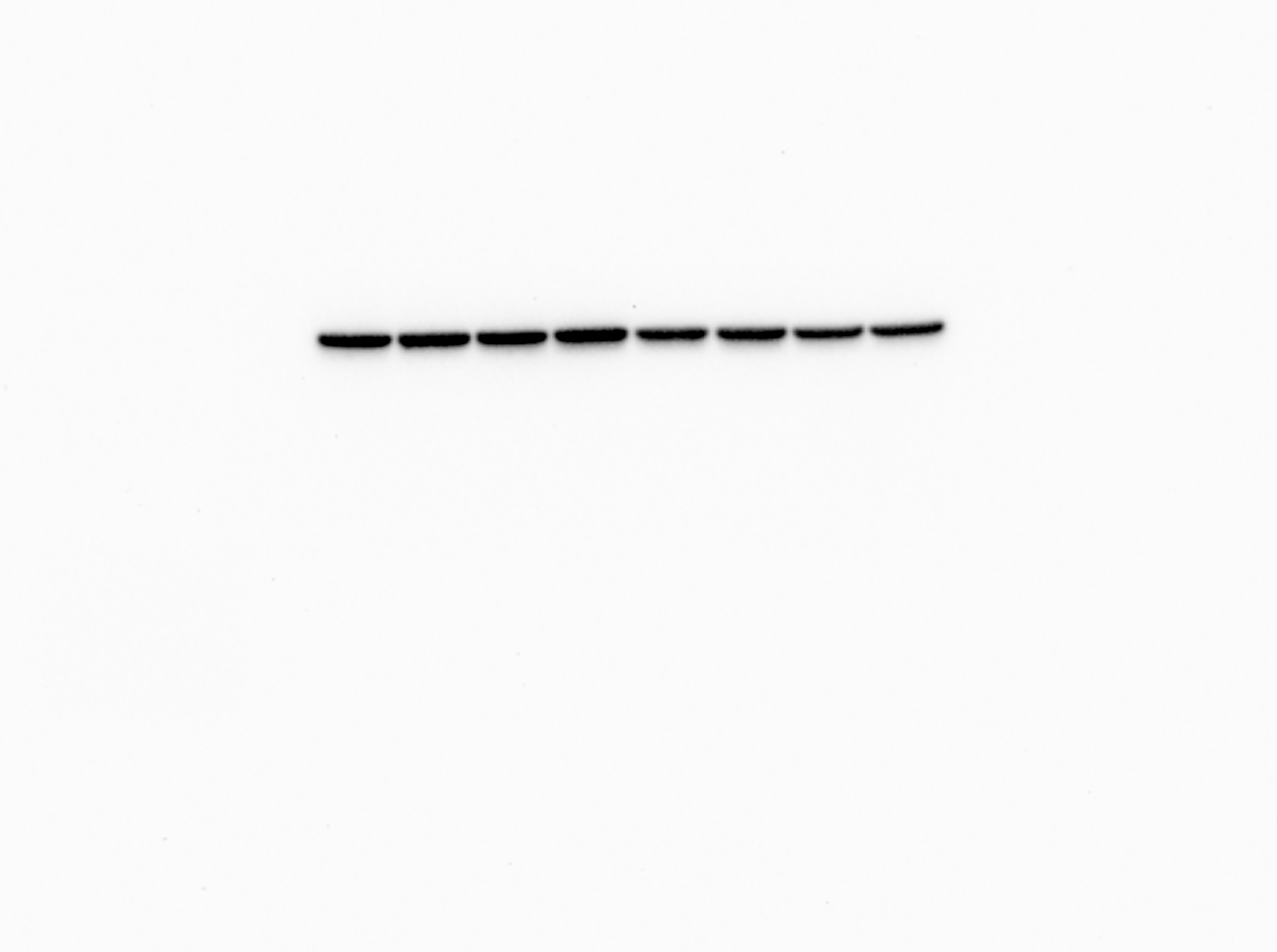

Supplement: Figure 4—figure supplement 1—source data 1. [file elife-85898-fig4-figsupp1-data1.zip › Figure 4-figure supplement 1-source data 1 - Copy/MCF7 actin.tif]

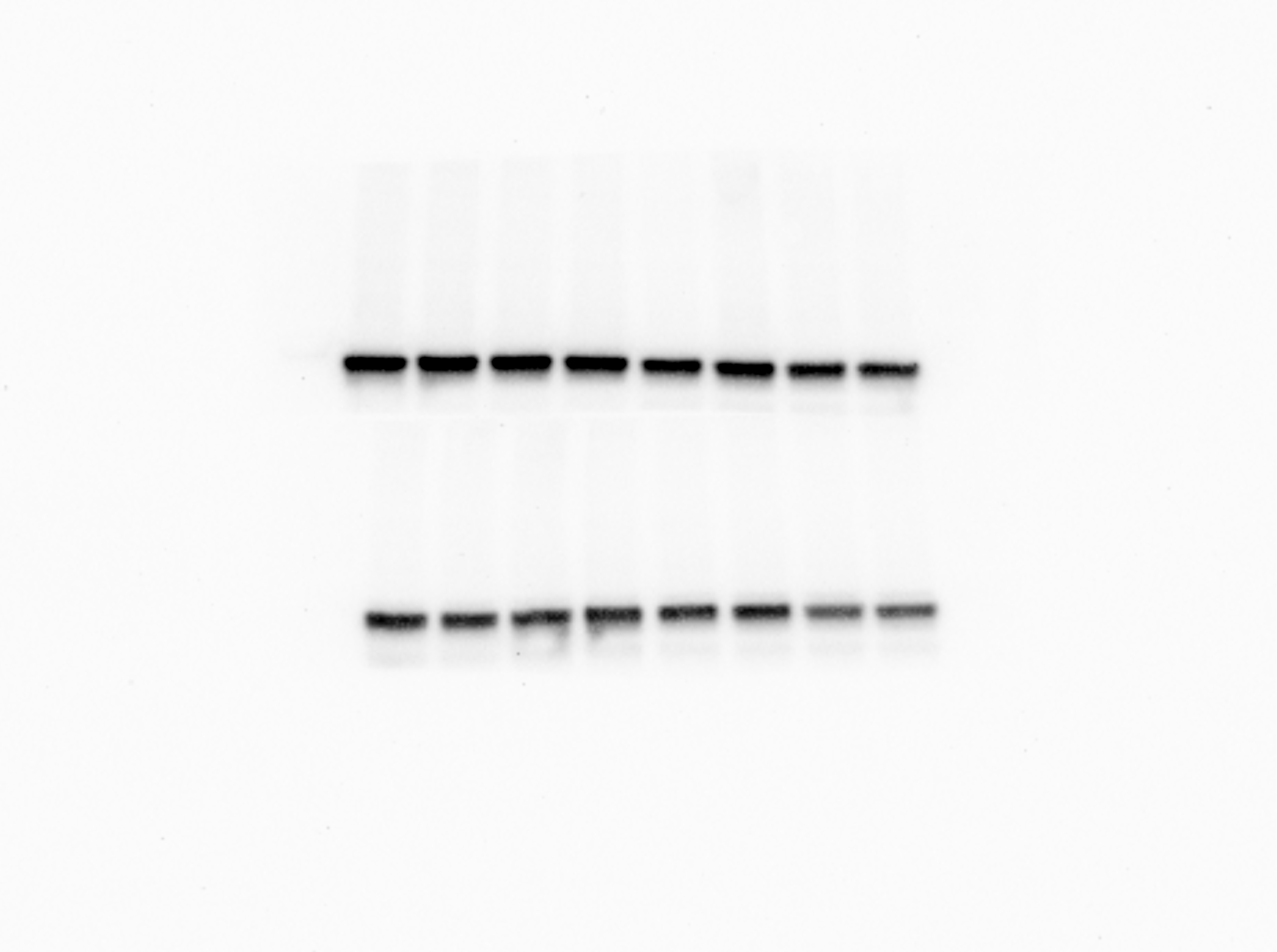

Supplement: Figure 4—figure supplement 1—source data 1. [file elife-85898-fig4-figsupp1-data1.zip › Figure 4-figure supplement 1-source data 1 - Copy/MCF7 AKT upper part.tif]

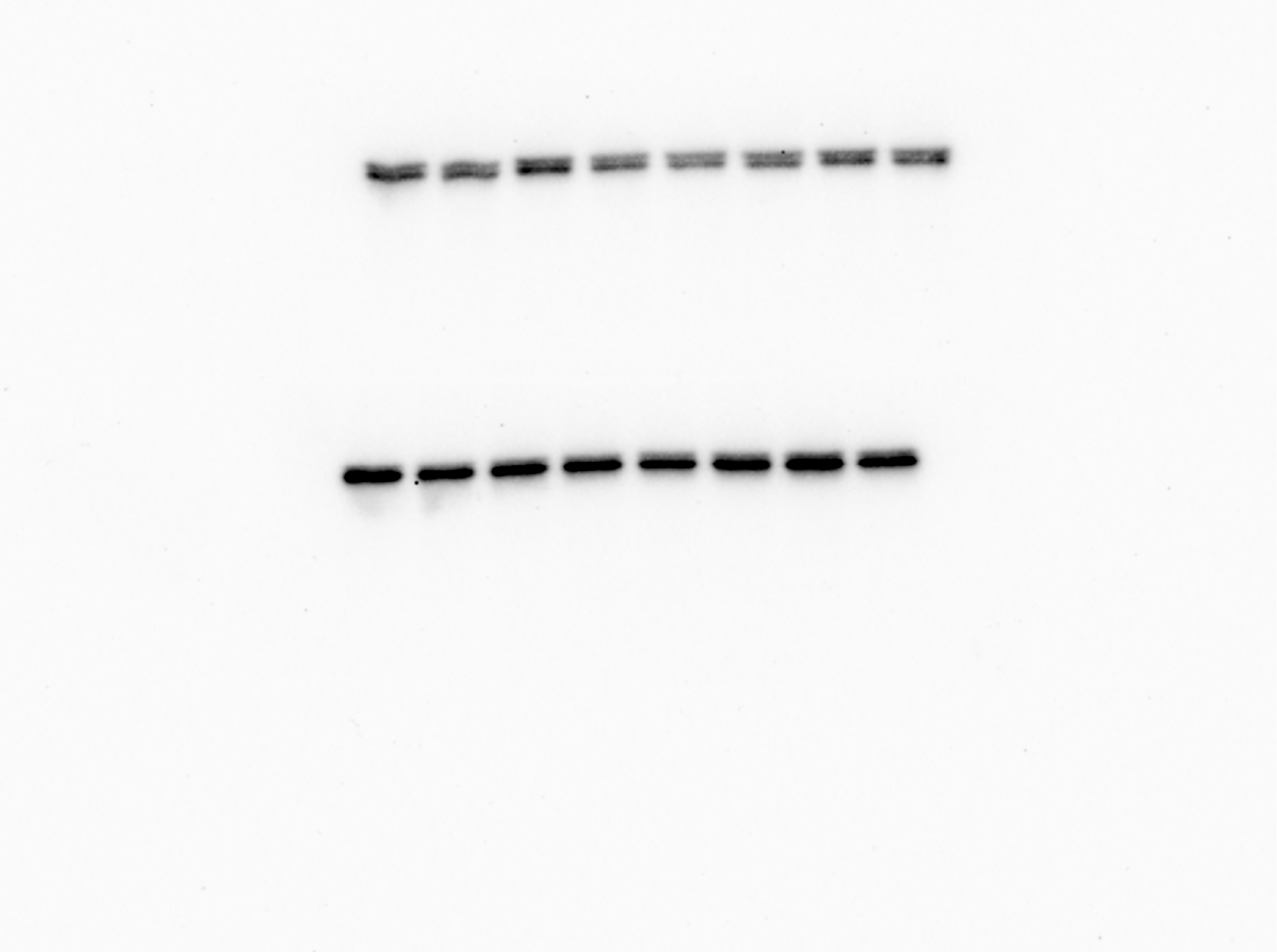

Supplement: Figure 4—figure supplement 1—source data 1. [file elife-85898-fig4-figsupp1-data1.zip › Figure 4-figure supplement 1-source data 1 - Copy/MCF7 ERK upper part.tif]

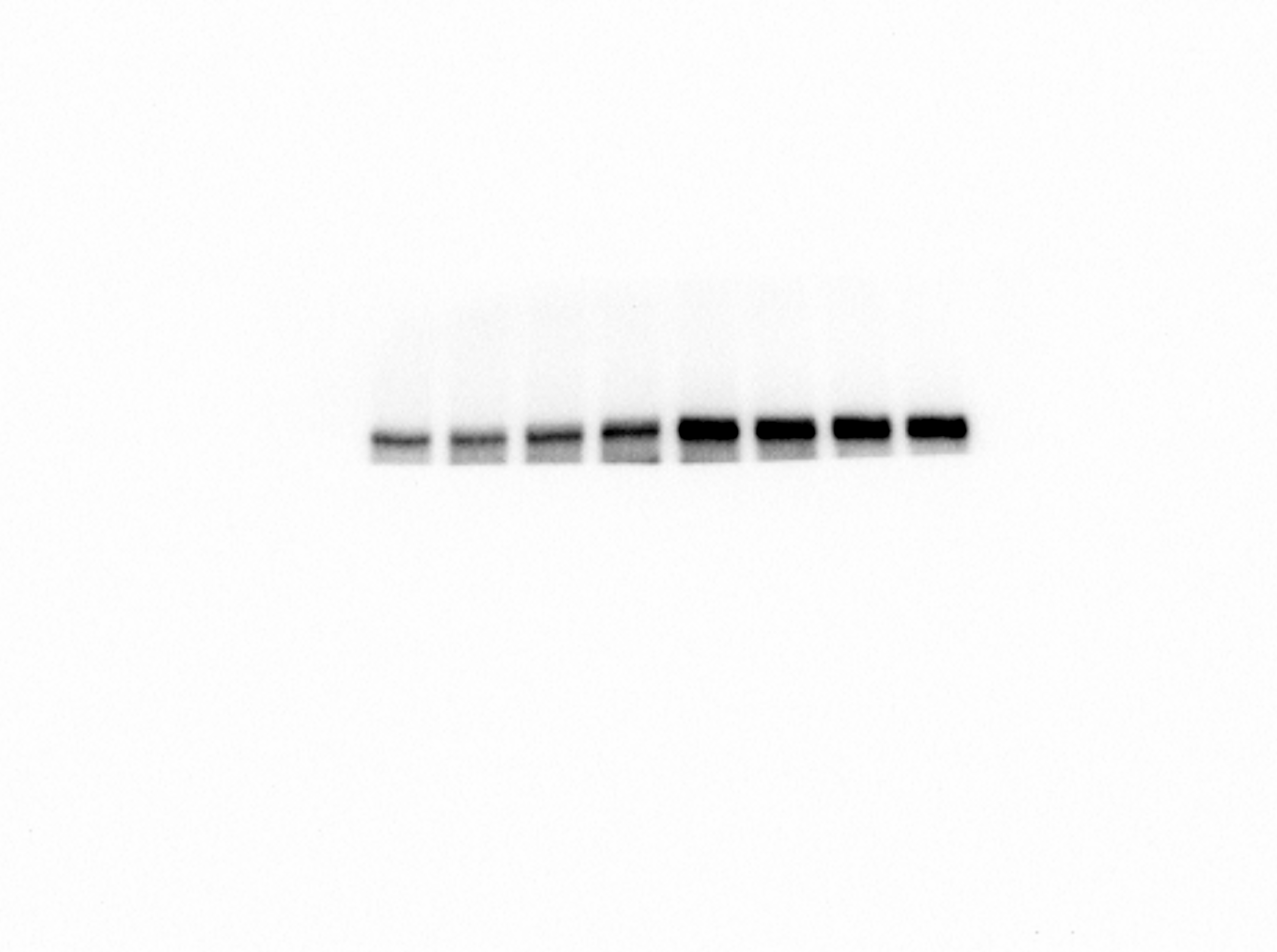

Supplement: Figure 4—figure supplement 1—source data 1. [file elife-85898-fig4-figsupp1-data1.zip › Figure 4-figure supplement 1-source data 1 - Copy/MCF7 FOXA3a.tif]

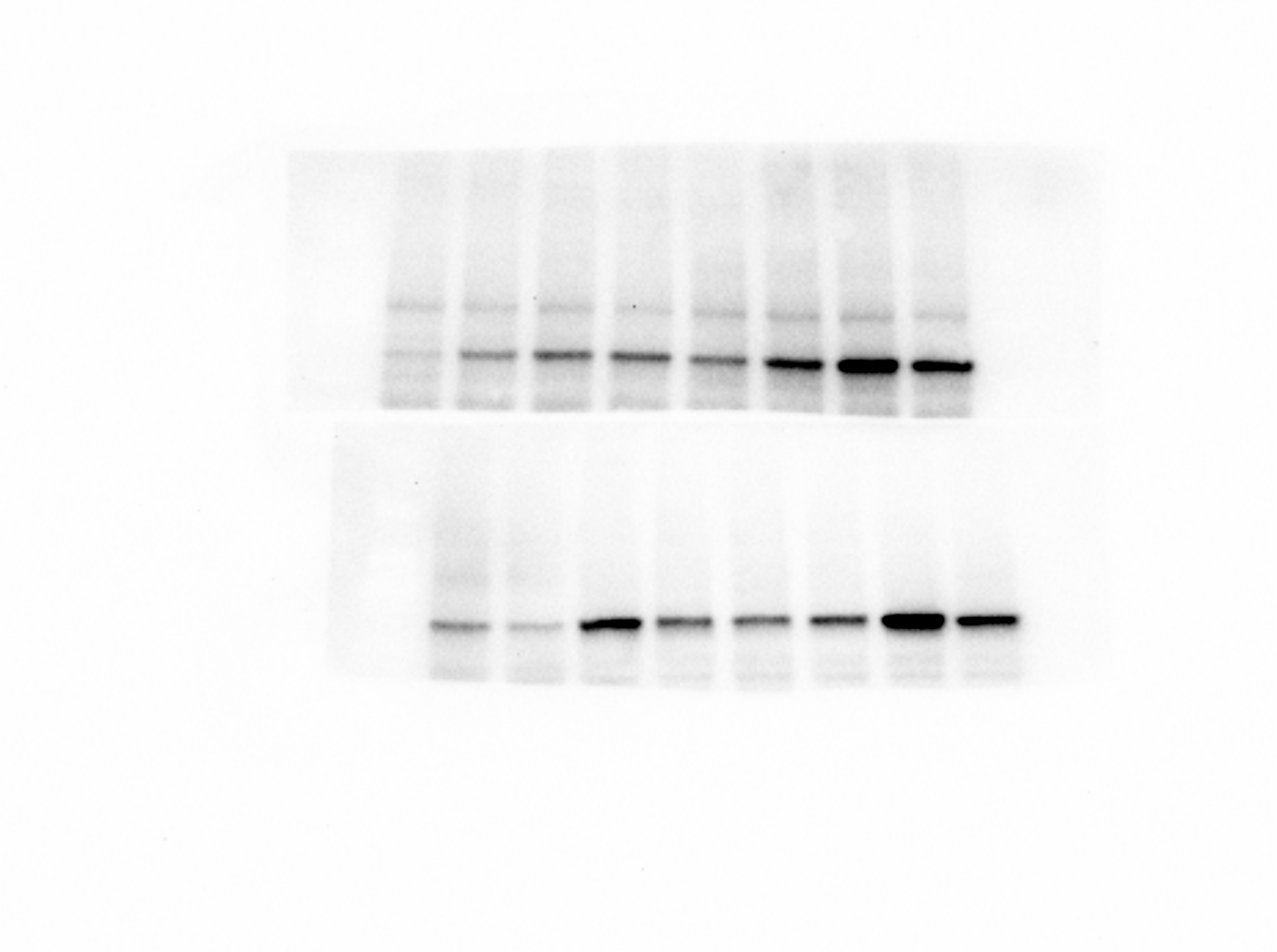

Supplement: Figure 4—figure supplement 1—source data 1. [file elife-85898-fig4-figsupp1-data1.zip › Figure 4-figure supplement 1-source data 1 - Copy/MCF7 pAKT upper part.tif]

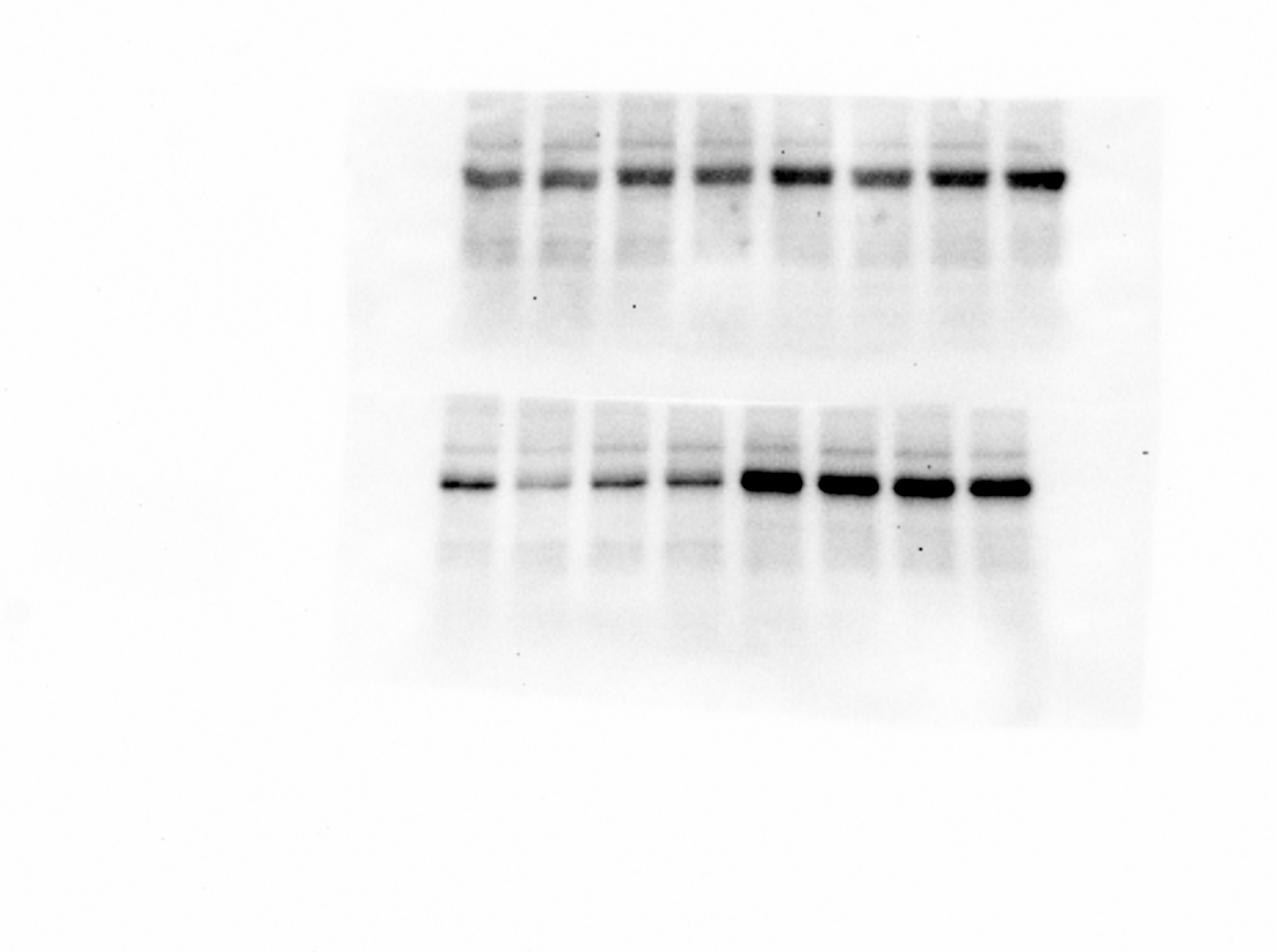

Supplement: Figure 4—figure supplement 1—source data 1. [file elife-85898-fig4-figsupp1-data1.zip › Figure 4-figure supplement 1-source data 1 - Copy/MCF7 pERK upper part.tif]

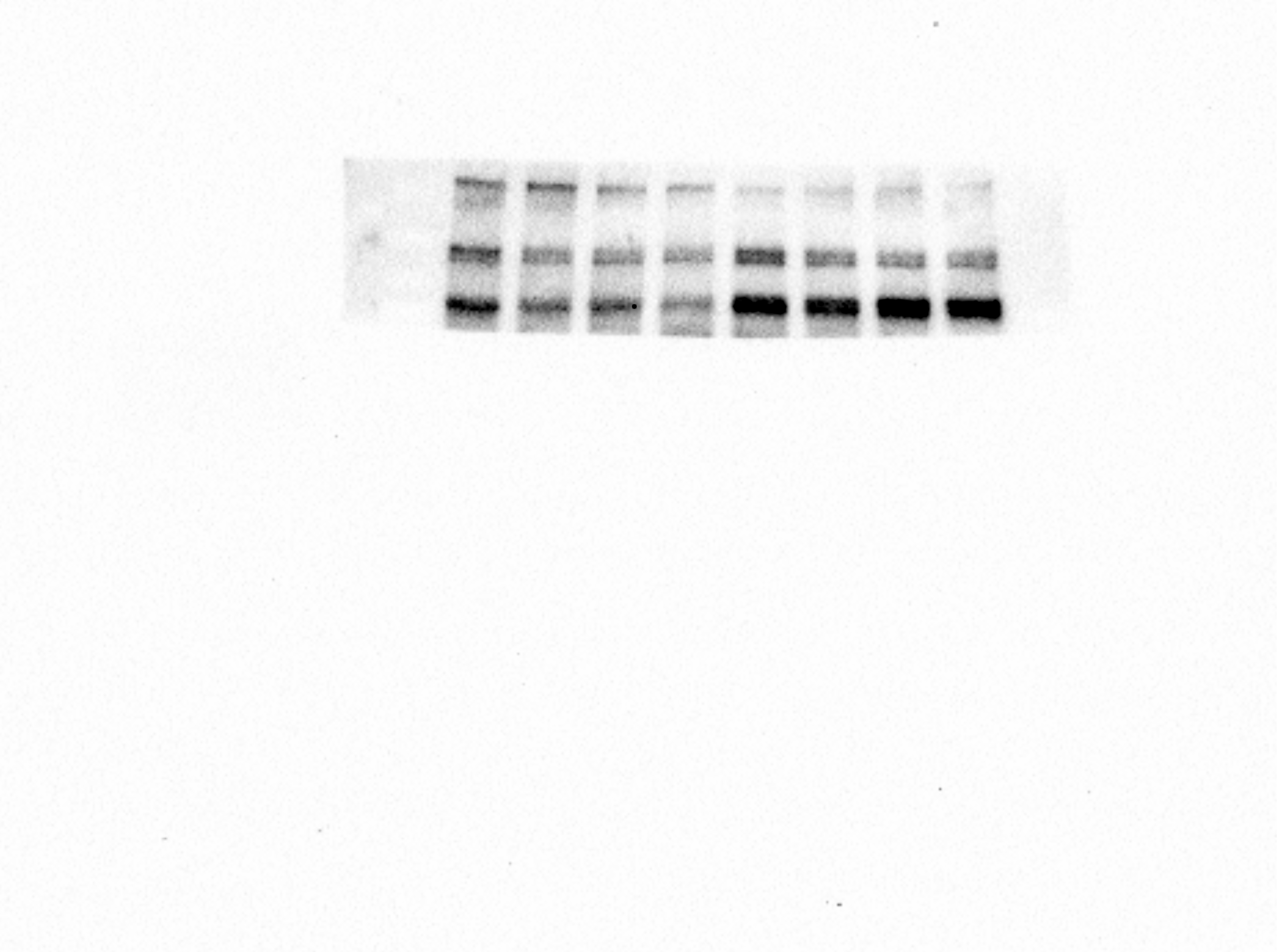

Supplement: Figure 4—figure supplement 1—source data 1. [file elife-85898-fig4-figsupp1-data1.zip › Figure 4-figure supplement 1-source data 1 - Copy/MCF7 pFOXA3a.tif]

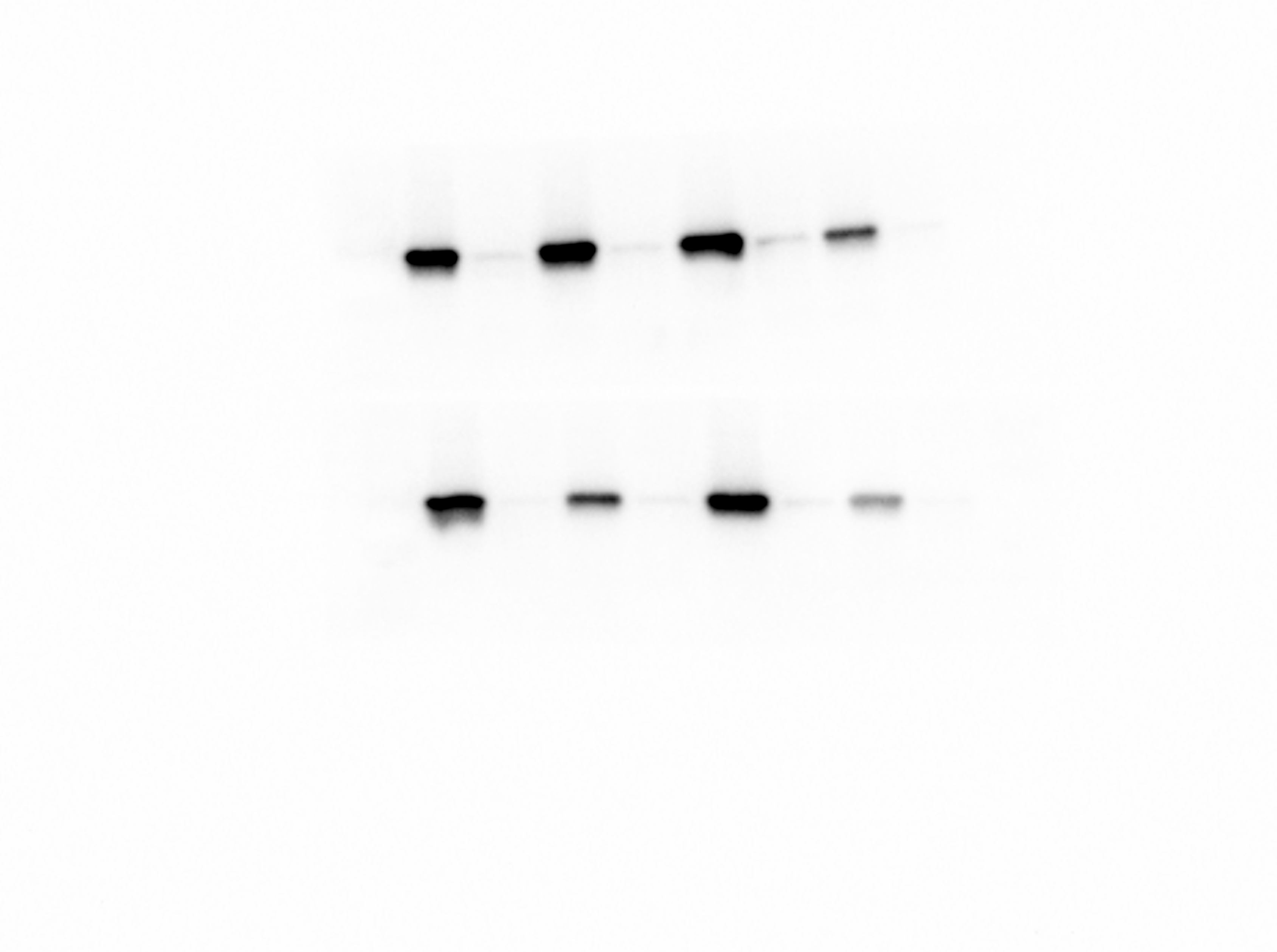

Supplement: Figure 4—figure supplement 1—source data 1. [file elife-85898-fig4-figsupp1-data1.zip › Figure 4-figure supplement 1-source data 1 - Copy/MCF7 pS6 upper part.tif]

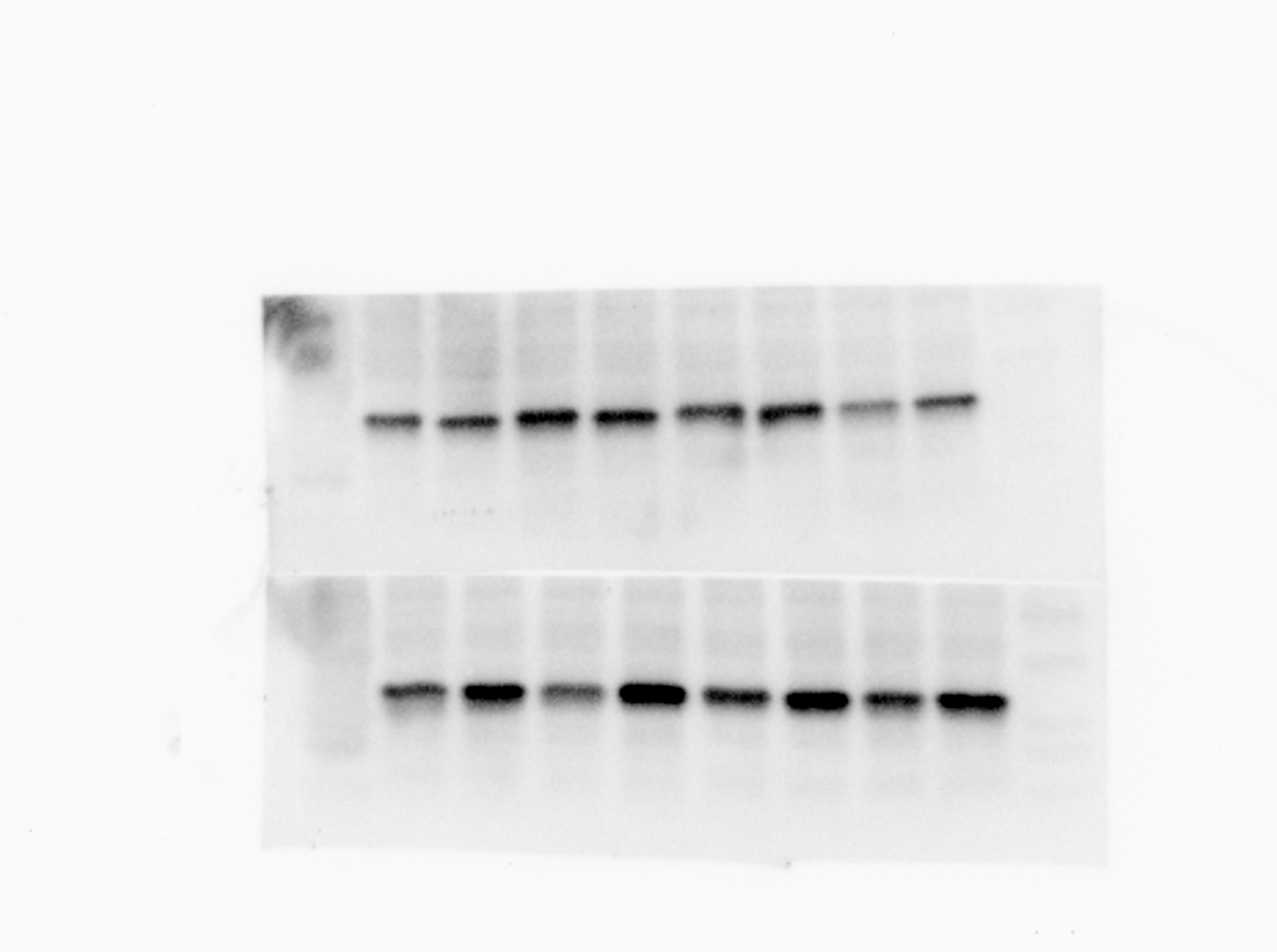

Supplement: Figure 4—figure supplement 1—source data 1. [file elife-85898-fig4-figsupp1-data1.zip › Figure 4-figure supplement 1-source data 1 - Copy/MCF7 S6 upper part.tif]

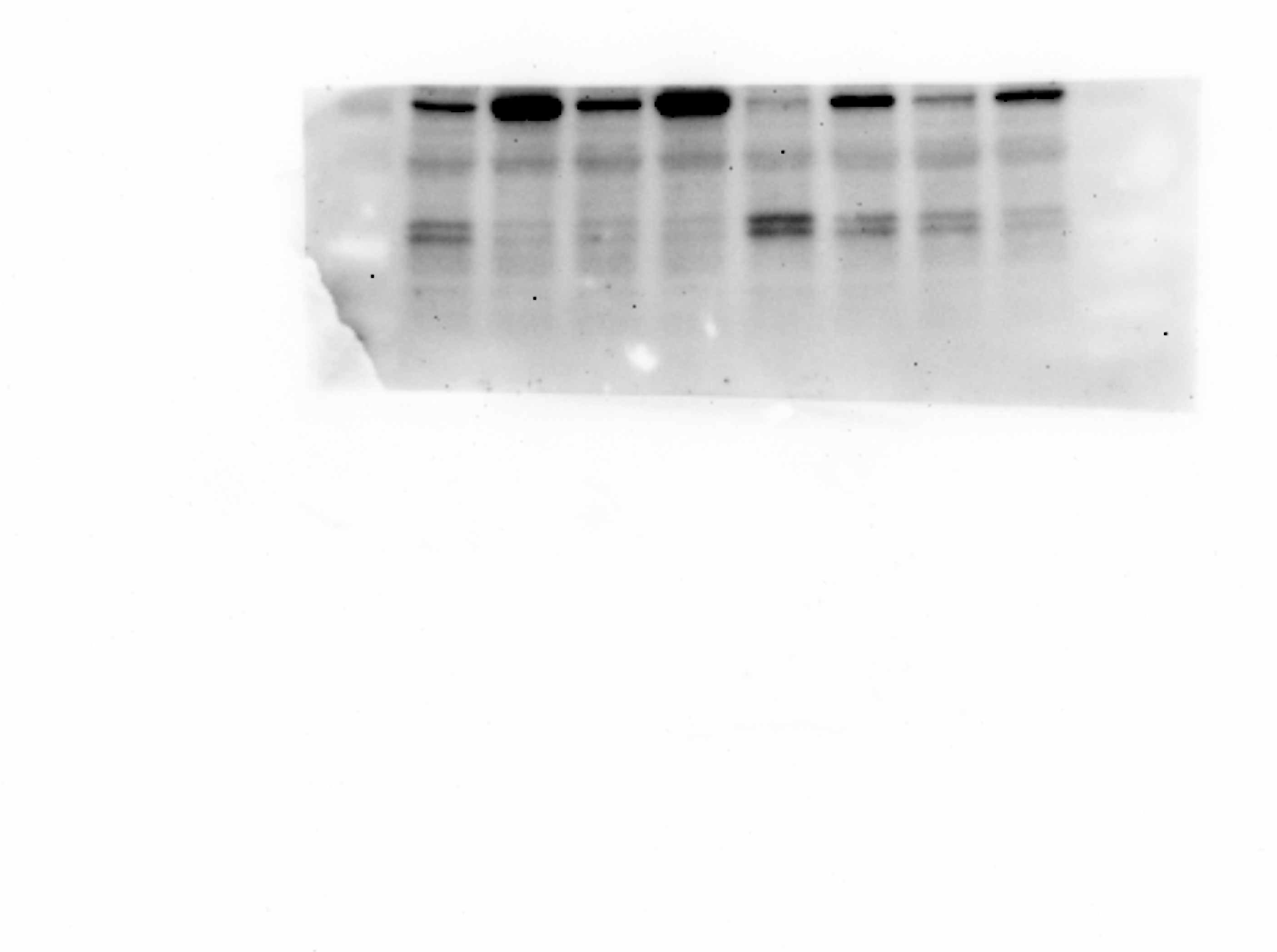

Supplement: Figure 4—figure supplement 1—source data 1. [file elife-85898-fig4-figsupp1-data1.zip › Figure 4-figure supplement 1-source data 1 - Copy/MCF7 TRAIL.tif]

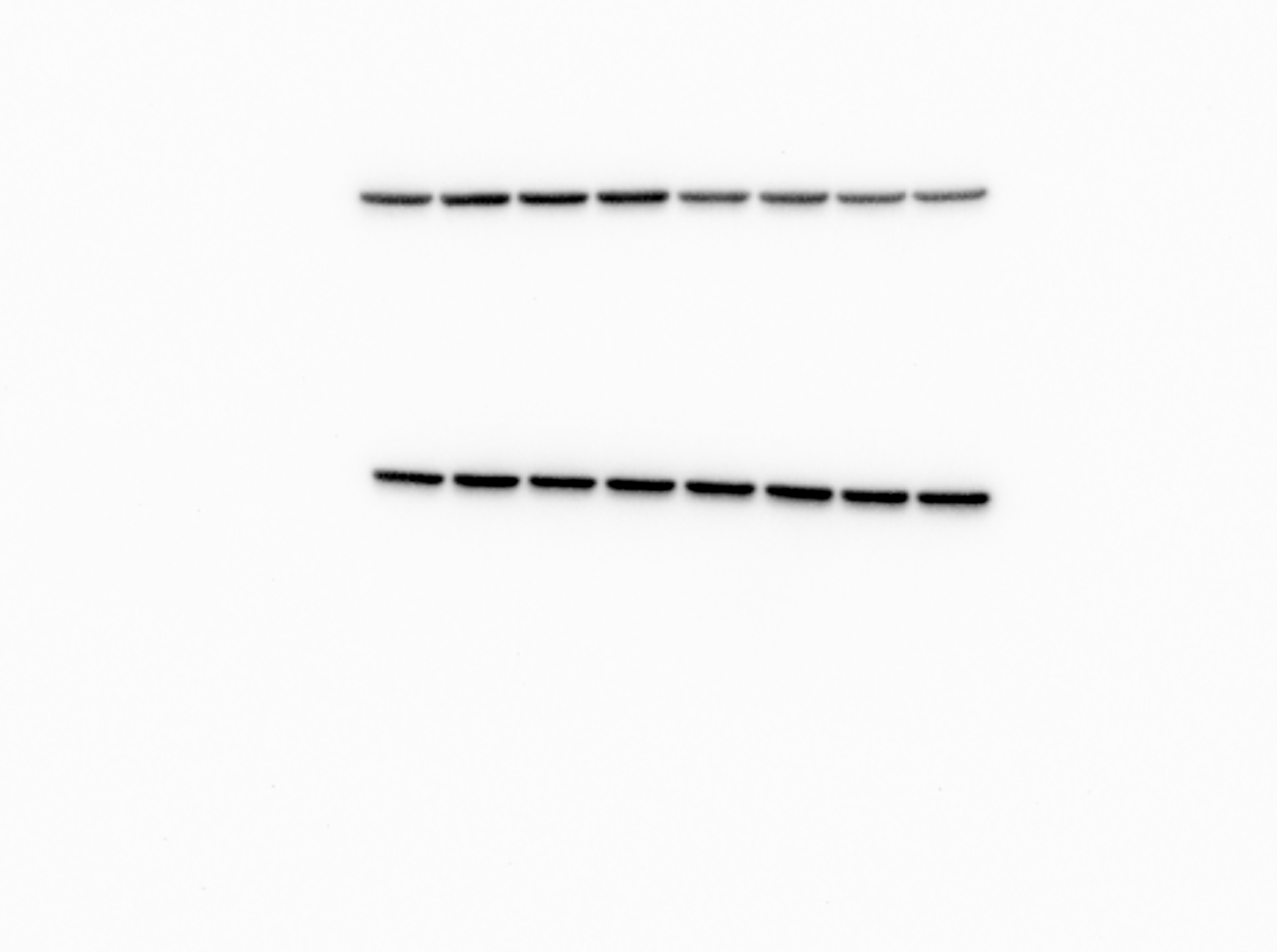

Supplement: Figure 4—figure supplement 1—source data 1. [file elife-85898-fig4-figsupp1-data1.zip › Figure 4-figure supplement 1-source data 1 - Copy/T47D actin lower part.tif]

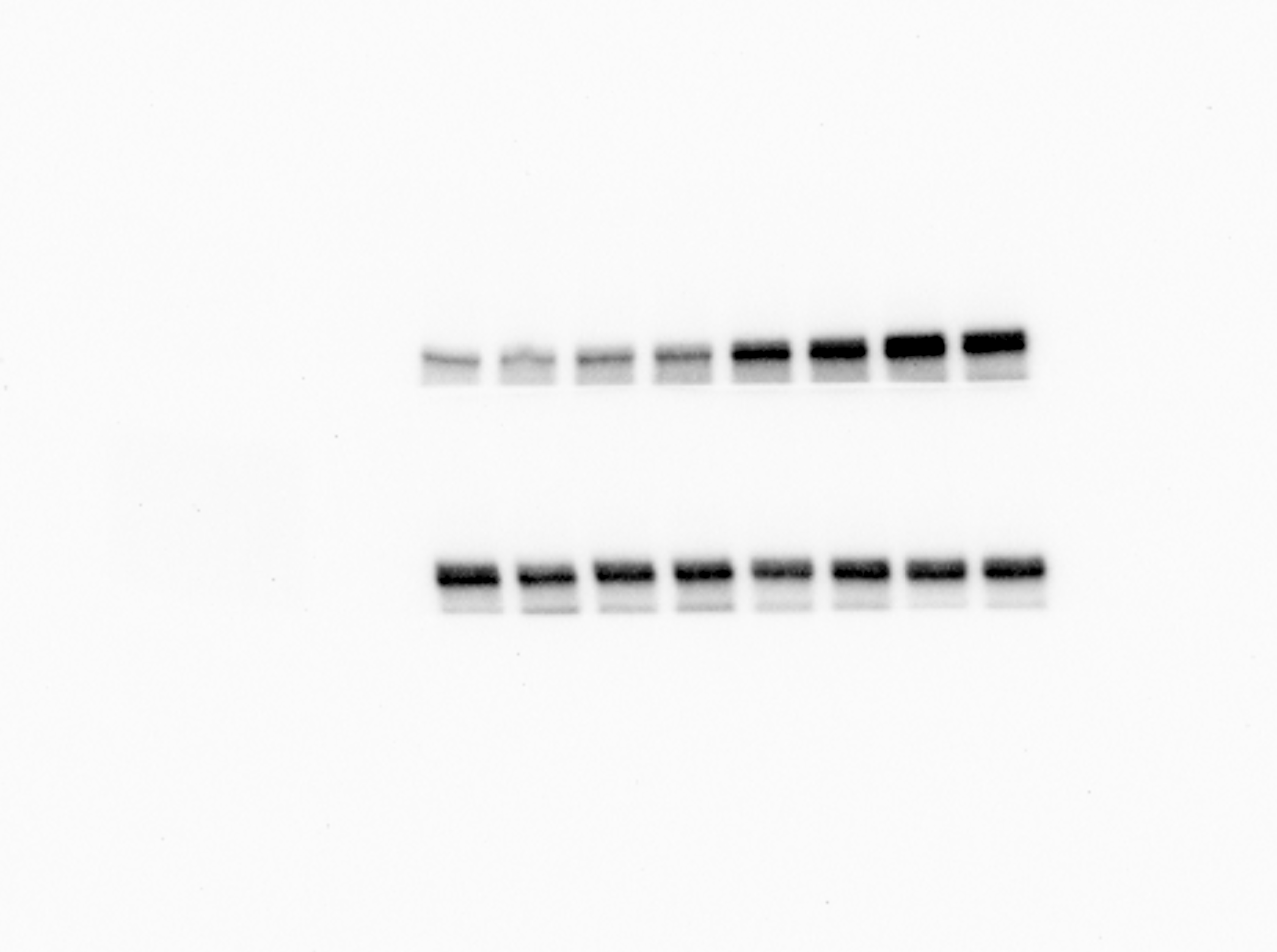

Supplement: Figure 4—figure supplement 1—source data 1. [file elife-85898-fig4-figsupp1-data1.zip › Figure 4-figure supplement 1-source data 1 - Copy/T47D FOXA3a lower part.tif]

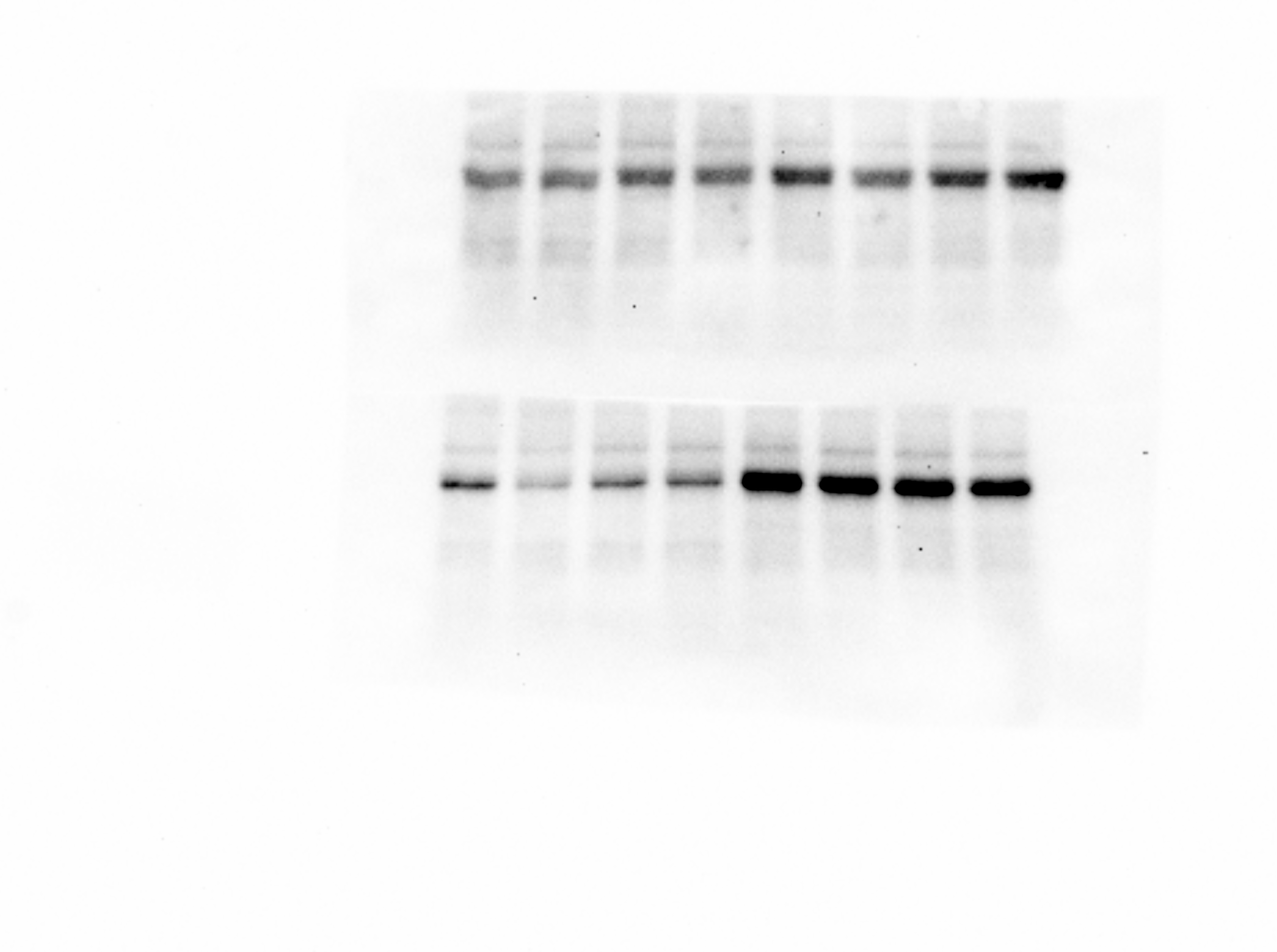

Supplement: Figure 4—figure supplement 1—source data 1. [file elife-85898-fig4-figsupp1-data1.zip › Figure 4-figure supplement 1-source data 1 - Copy/T47D pERK lower part.tif]

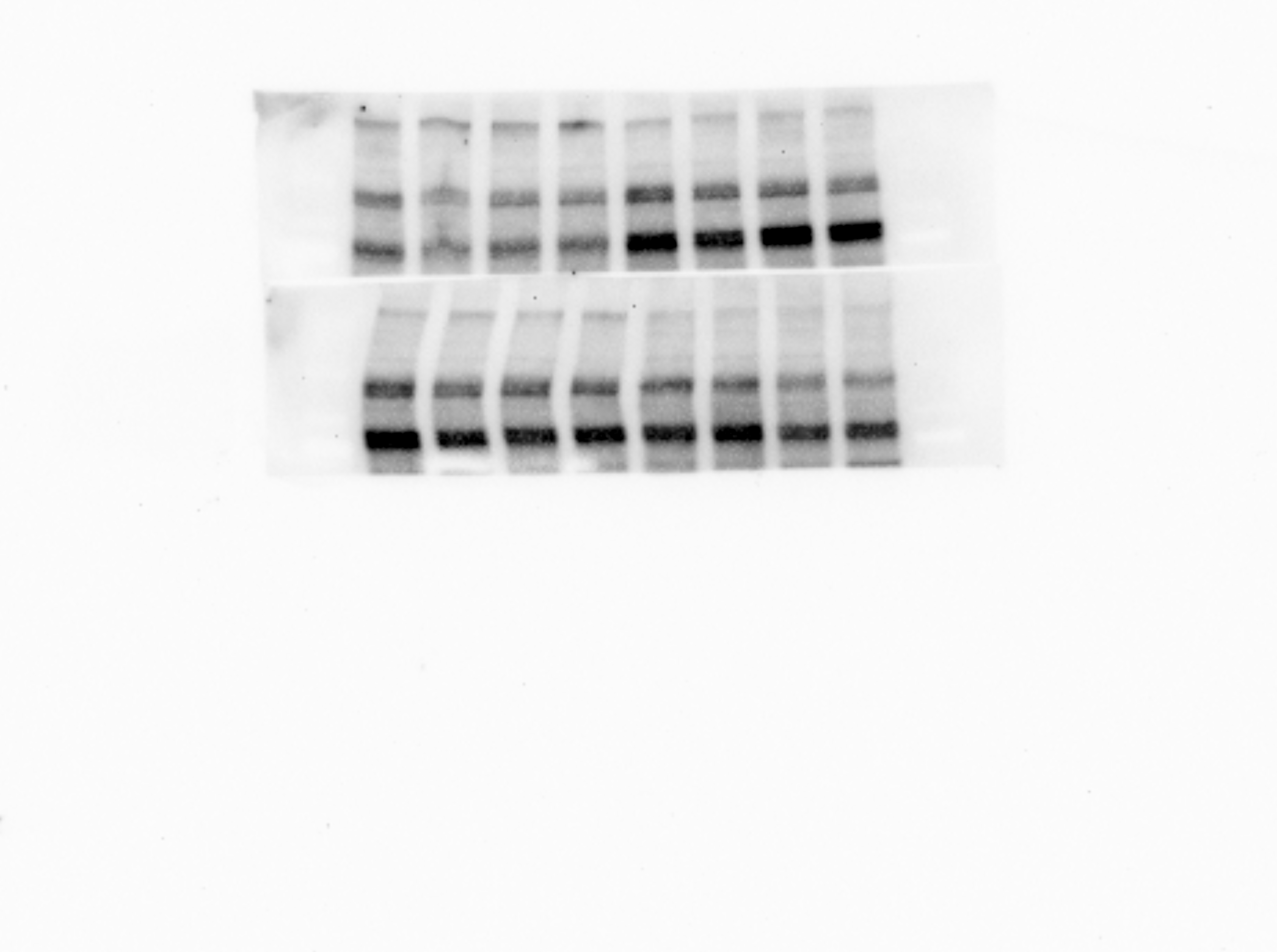

Supplement: Figure 4—figure supplement 1—source data 1. [file elife-85898-fig4-figsupp1-data1.zip › Figure 4-figure supplement 1-source data 1 - Copy/T47D pFOXA3a lower part.tif]

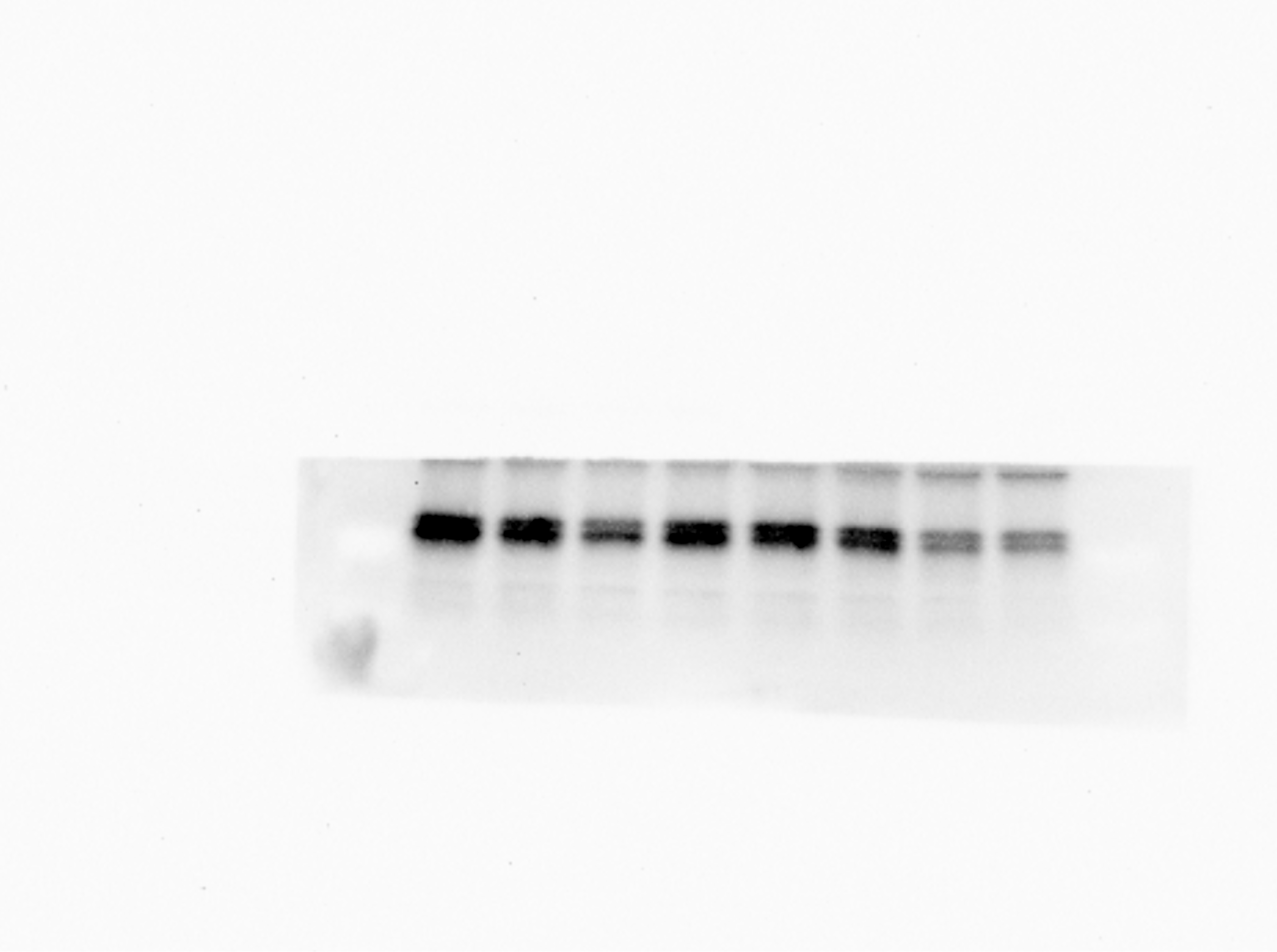

Supplement: Figure 4—figure supplement 1—source data 1. [file elife-85898-fig4-figsupp1-data1.zip › Figure 4-figure supplement 1-source data 1 - Copy/T47D TRAIL.tif]

CAMA-1

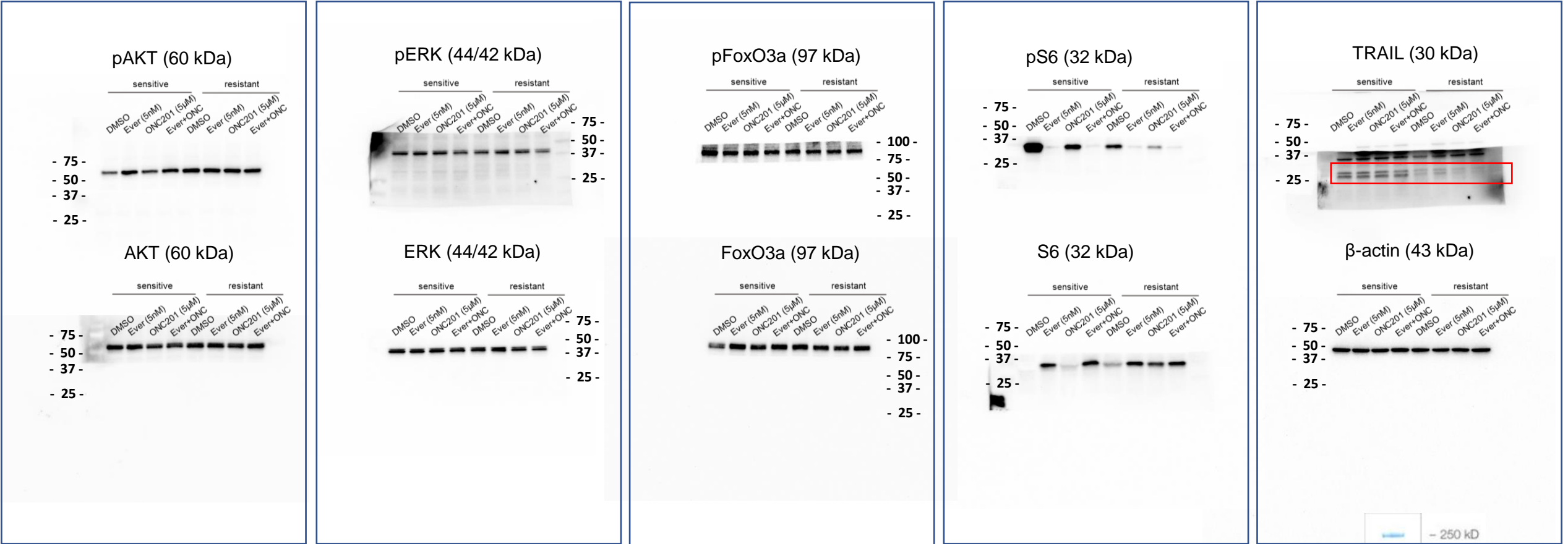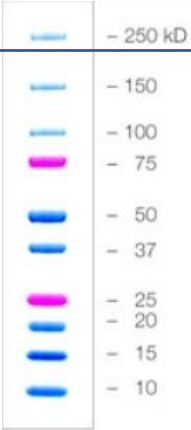

# MCF7

pAKT (60 kDa)

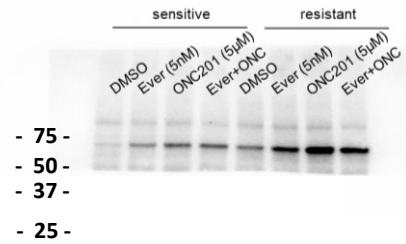

AKT (60 kDa)

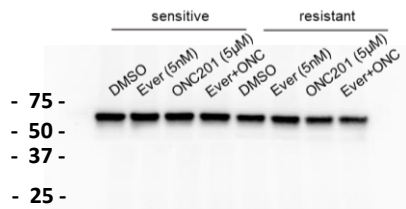

pERK (44/42 kDa)

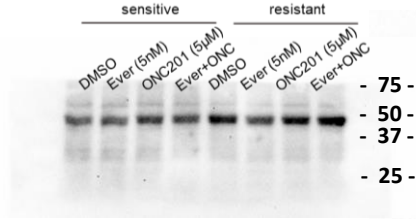

ERK (44/42 kDa)

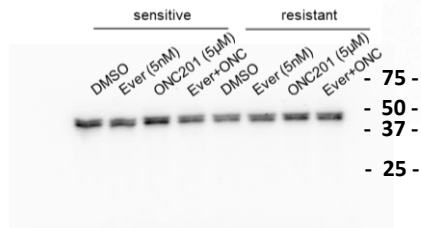

pFoxO3a (97 kDa)

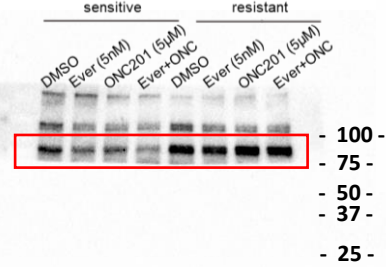

FoxO3a (97 kDa)

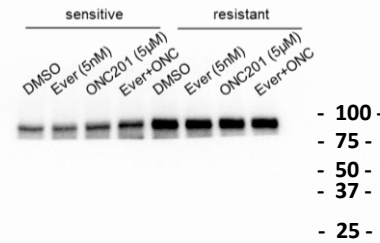

pS6 (32 kDa)

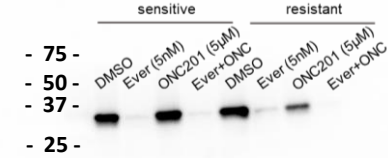

S6 (32 kDa)

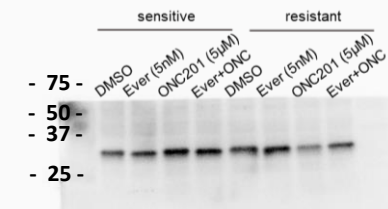

TRAIL (30 kDa)

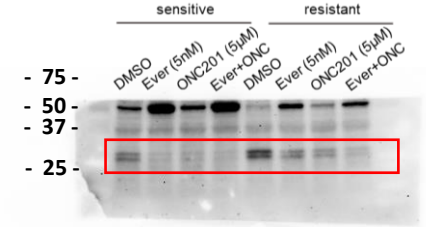

β-actin (43 kDa)

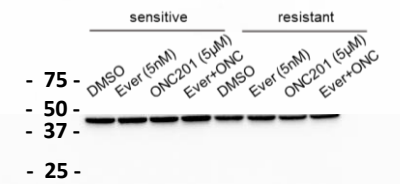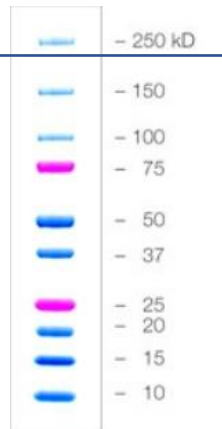

# T47D

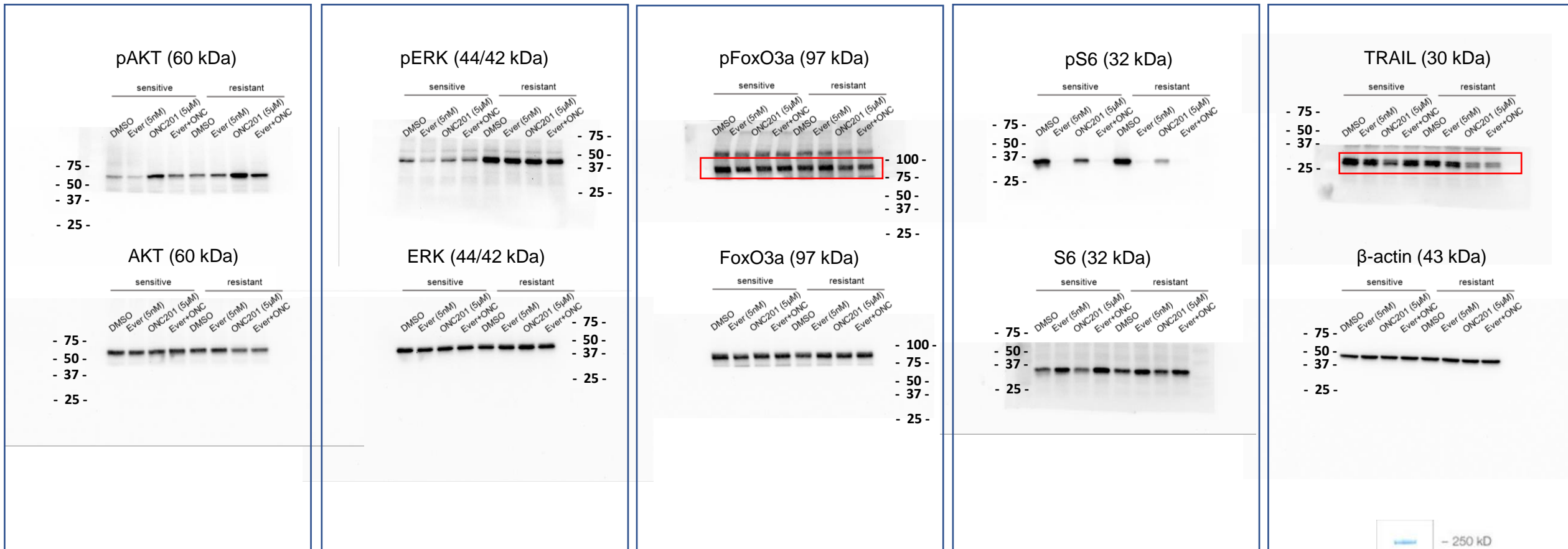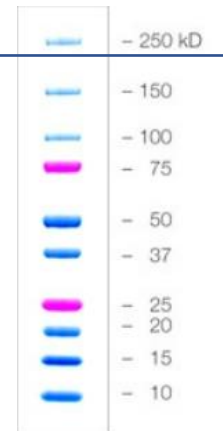

Supplement: Figure 4—figure supplement 1—source data 2. [file elife-85898-fig4-figsupp1-data2.pdf]
