# Supplementary material for: ONC201/TIC10 enhances durability of mTOR inhibitor everolimus in metastatic ER+ breast cancer
Source: eLife. 2023 Sep 29;12:e85898. doi: 10.7554/eLife.85898 (PMC10541180; doi:10.7554/eLife.85898)
Supplement: Figure 4—source data 3. [file elife-85898-fig4-data3.pdf]

CAMA-1

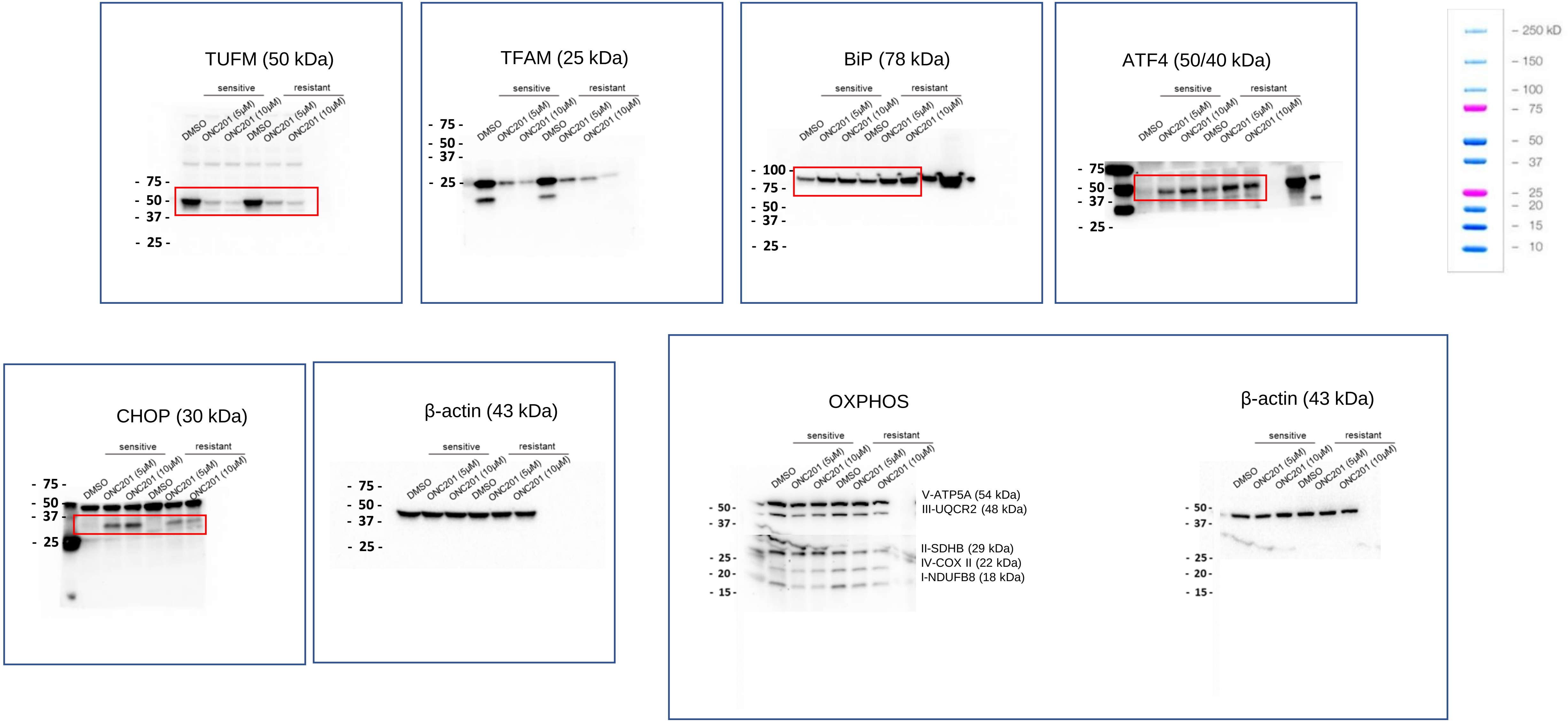
TUFM (50 kDa)
sensitive
resistant
DMSO
ONC201 (5μM)
ONC201 (10μM)
DMSO
ONC201 (5μM)
ONC201 (10μM)
75
50
37
25
TFAM (25 kDa)
BiP (78 kDa)
100
ATF4 (50/40 kDa)
250 kD
150
100
75
50
37
25
20
15
10
CHOP (30 kDa)
β-actin (43 kDa)
OXPHOS
V-ATP5A (54 kDa)
III-UQCR2 (48 kDa)
II-SDHB (29 kDa)
IV-COX II (22 kDa)
I-NDUFB8 (18 kDa)
β-actin (43 kDa)

MCF7

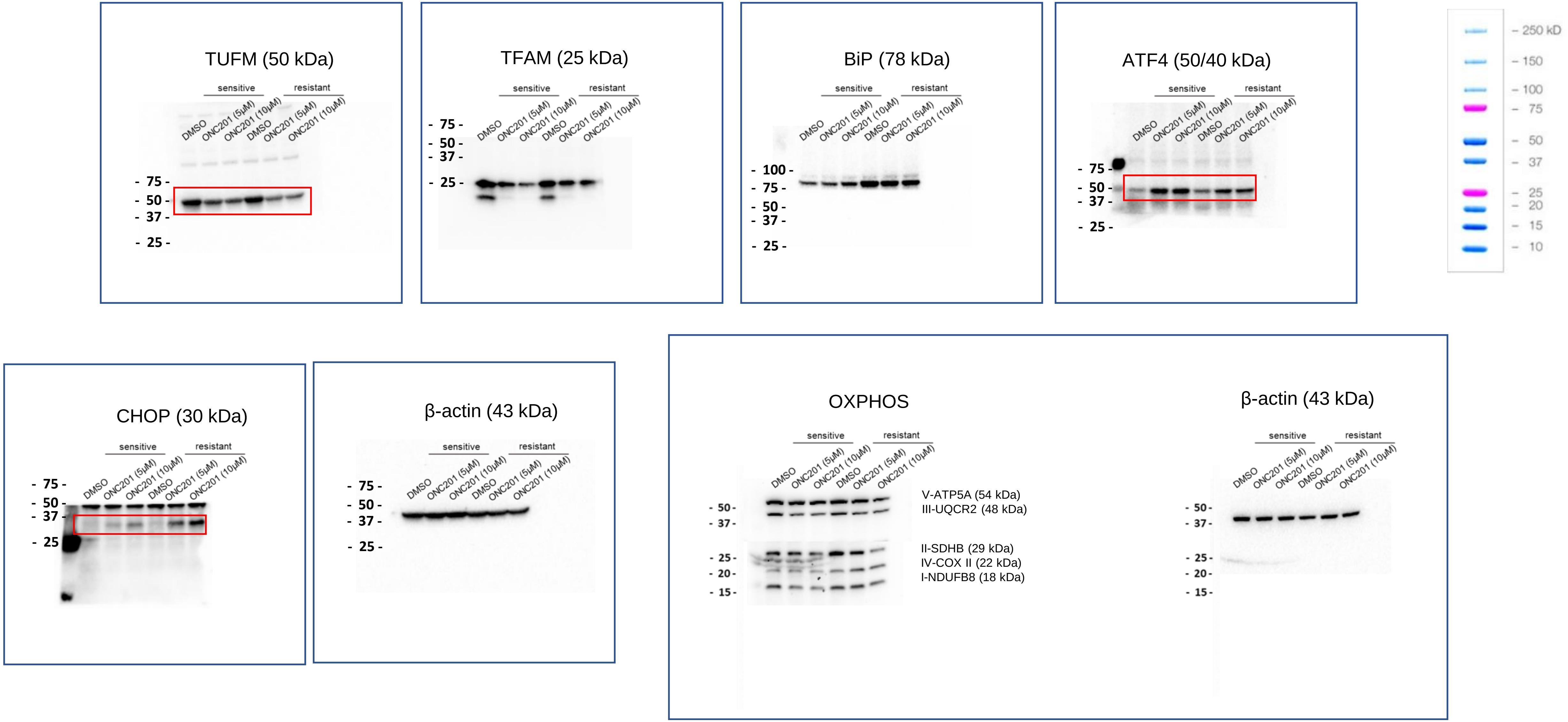

TUFM (50 kDa)
TFAM (25 kDa)
BiP (78 kDa)
ATF4 (50/40 kDa)
CHOP (30 kDa)
β-actin (43 kDa)
OXPHOS
β-actin (43 kDa)
sensitive
resistant
DMSO
ONC201 (5μM)
ONC201 (10μM)
V-ATP5A (54 kDa)
III-UQCR2 (48 kDa)
II-SDHB (29 kDa)
IV-COX II (22 kDa)
I-NDUFB8 (18 kDa)
250 kD
150
100
75
50
37
25
20
15
10

T47D

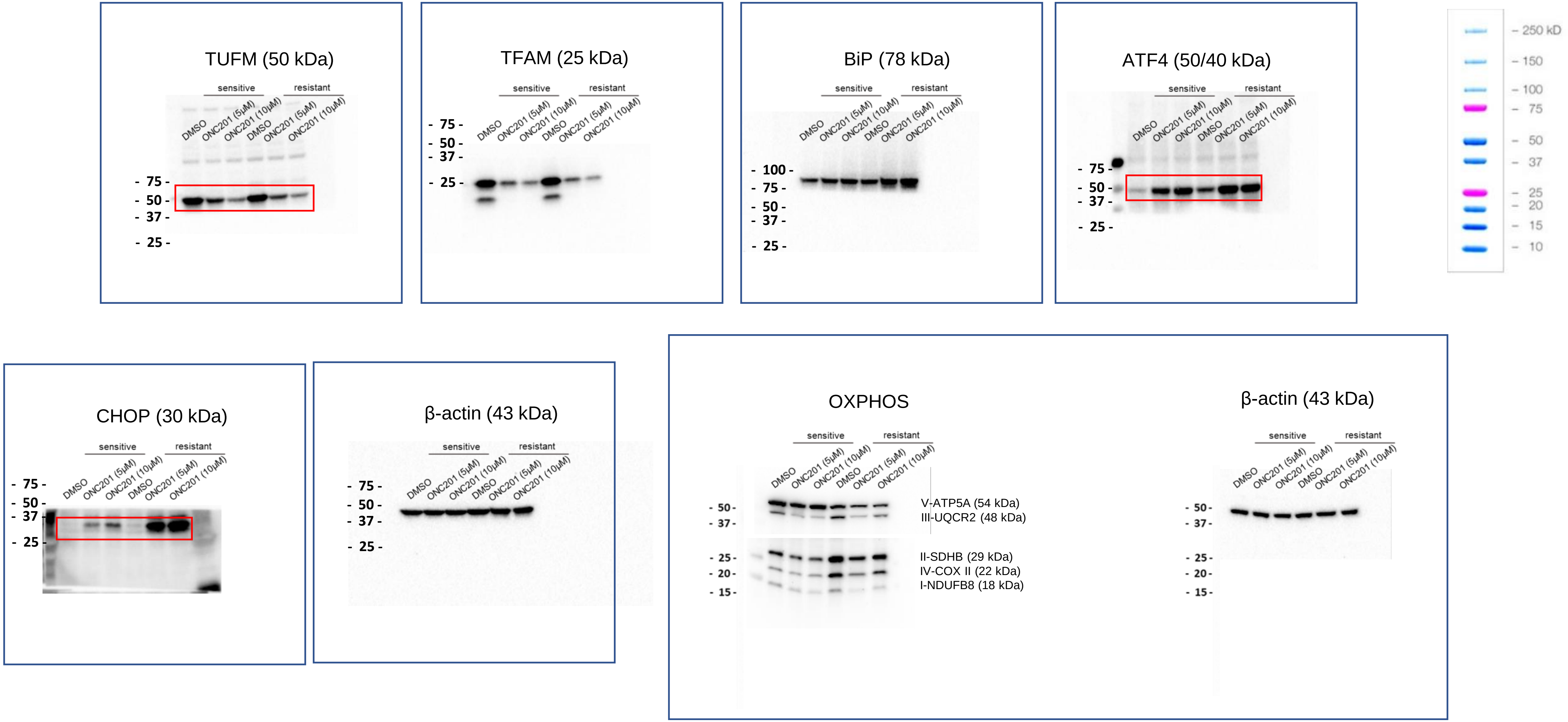
TUFM (50 kDa)
TFAM (25 kDa)
BiP (78 kDa)
ATF4 (50/40 kDa)
CHOP (30 kDa)
β-actin (43 kDa)
OXPHOS
β-actin (43 kDa)
sensitive
resistant
DMSO
ONC201 (5μM)
ONC201 (10μM)
DMSO
ONC201 (5μM)
ONC201 (10μM)
V-ATP5A (54 kDa)
III-UQCR2 (48 kDa)
II-SDHB (29 kDa)
IV-COX II (22 kDa)
I-NDUFB8 (18 kDa)
250 kD
150
100
75
50
37
25
20
15
10

CAMA-1

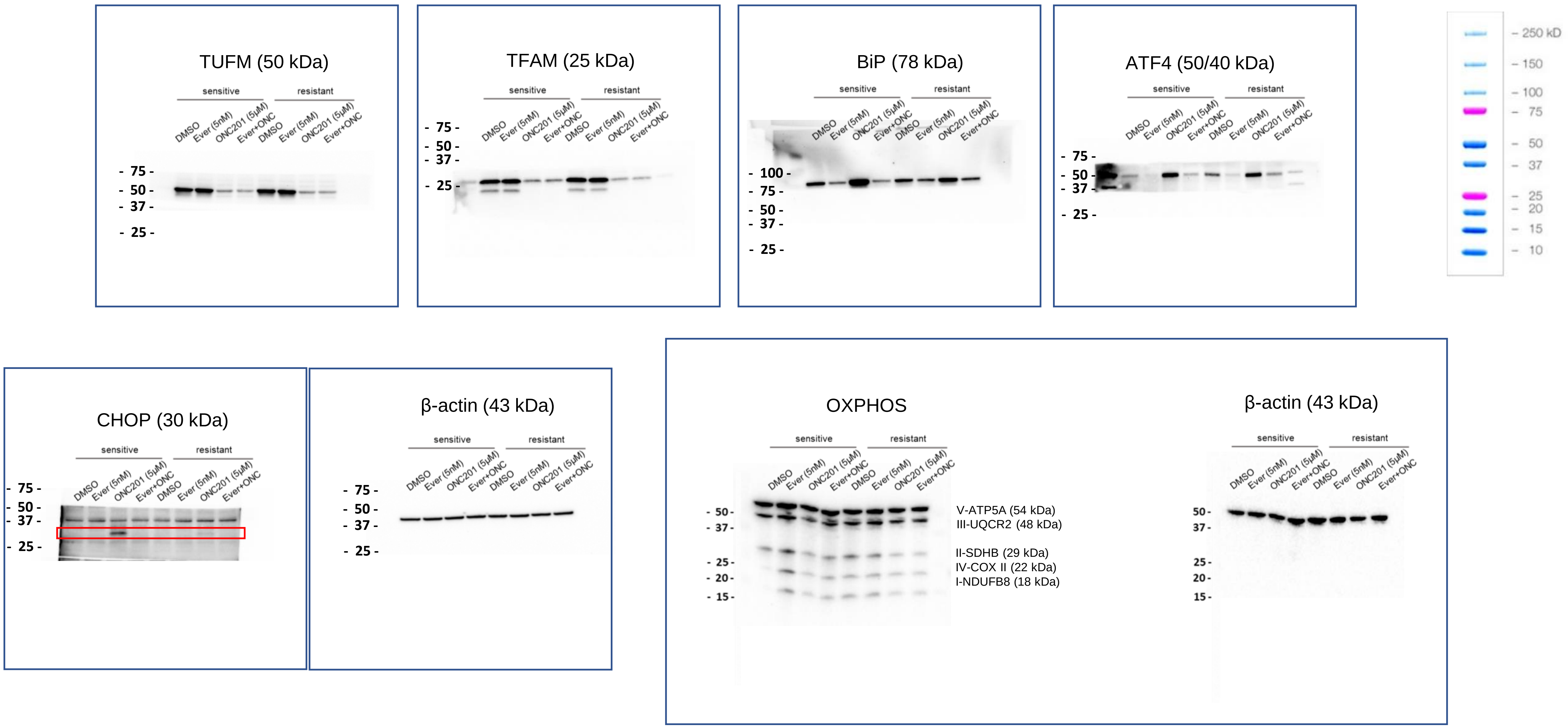

TUFM (50 kDa)
TFAM (25 kDa)
BiP (78 kDa)
ATF4 (50/40 kDa)
CHOP (30 kDa)
β-actin (43 kDa)
OXPHOS
β-actin (43 kDa)
sensitive
resistant
DMSO
Ever (5nM)
ONC201 (5μM)
Ever+ONC
V-ATP5A (54 kDa)
III-UQCR2 (48 kDa)
II-SDHB (29 kDa)
IV-COX II (22 kDa)
I-NDUFB8 (18 kDa)
250 kD
150
100
75
50
37
25
20
15
10

MCF7

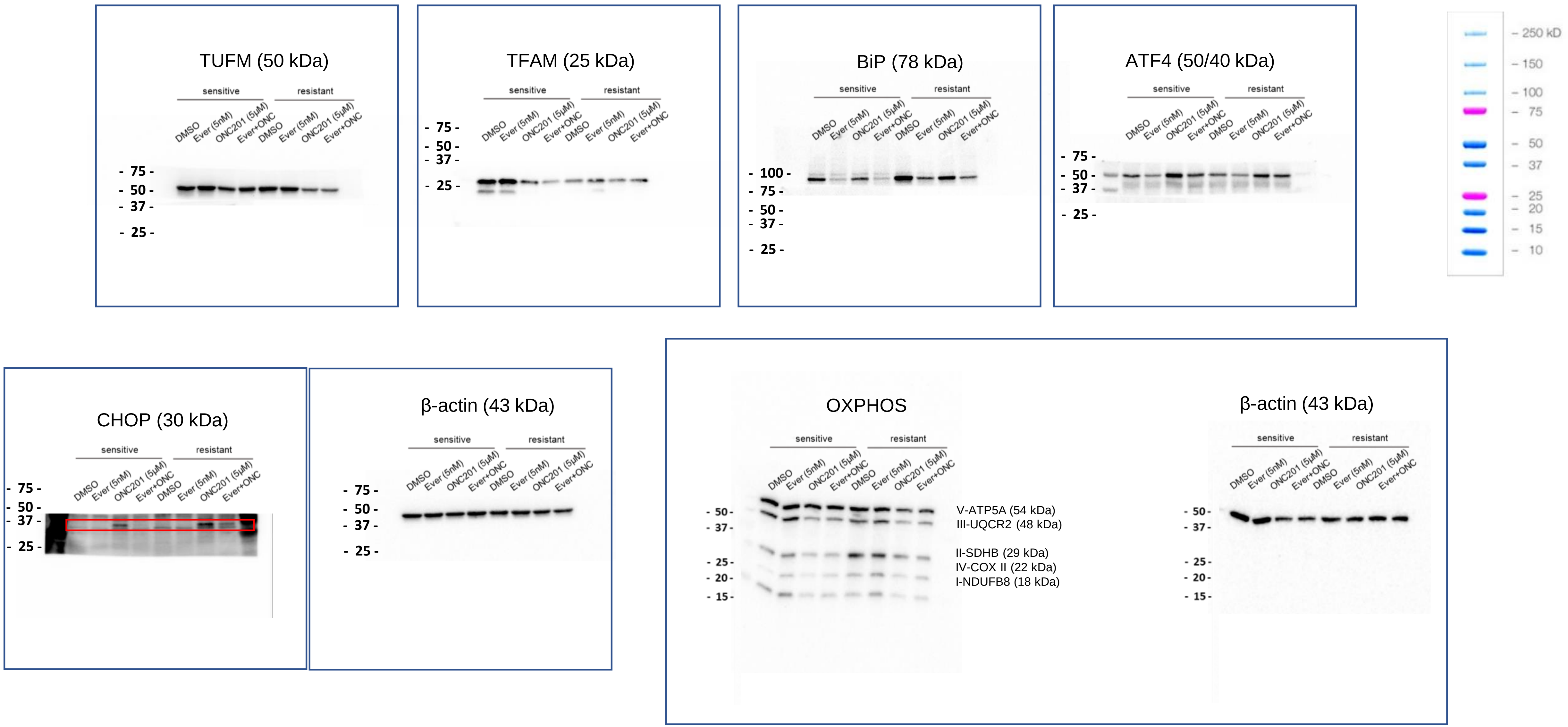
TUFM (50 kDa)
TFAM (25 kDa)
BiP (78 kDa)
ATF4 (50/40 kDa)
CHOP (30 kDa)
β-actin (43 kDa)
OXPHOS
β-actin (43 kDa)
sensitive
resistant
DMSO
Ever (5nM)
ONC201 (5μM)
Ever+ONC
V-ATP5A (54 kDa)
III-UQCR2 (48 kDa)
II-SDHB (29 kDa)
IV-COX II (22 kDa)
I-NDUFB8 (18 kDa)
250 kD
150
100
75
50
37
25
20
15
10

T47D

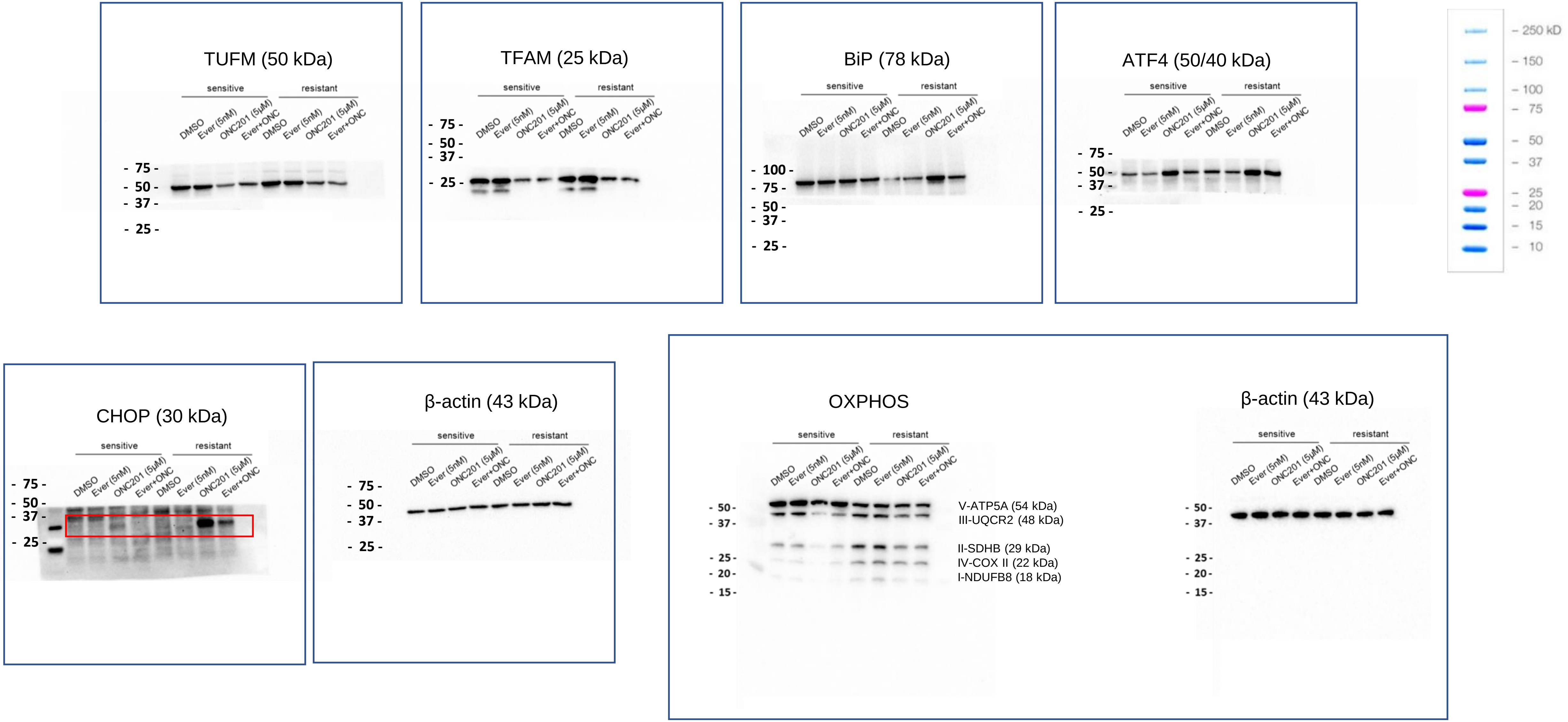
TUFM (50 kDa)
TFAM (25 kDa)
BiP (78 kDa)
ATF4 (50/40 kDa)
CHOP (30 kDa)
β-actin (43 kDa)
OXPHOS
β-actin (43 kDa)
sensitive
resistant
DMSO
Ever (5nM)
ONC201 (5µM)
Ever+ONC
V-ATP5A (54 kDa)
III-UQCR2 (48 kDa)
II-SDHB (29 kDa)
IV-COX II (22 kDa)
I-NDUFB8 (18 kDa)
250 kD
150
100
75
50
37
25
20
15
10
